# Low-cost electrochemical detection of arsenic in the groundwater of Guanajuato state, central Mexico using an open-source potentiostat

**DOI:** 10.1371/journal.pone.0262124

**Published:** 2022-01-19

**Authors:** Jay C. Bullen, Lawrence N. Dworsky, Martijn Eikelboom, Matthieu Carriere, Alexandra Alvarez, Pascal Salaün

**Affiliations:** 1 Department of Earth Science and Engineering, Faculty of Engineering, Imperial College London, London, United Kingdom; 2 Caminos de Agua, San Miguel de Allende, Guanajuato, Mexico; 3 Department of Earth, Ocean and Ecological Sciences, School of Environmental Sciences, University of Liverpool, Liverpool, United Kingdom; Hamadan University of Medical Sciences, ISLAMIC REPUBLIC OF IRAN

## Abstract

Arsenic is a carcinogenic groundwater contaminant that is toxic even at the parts-per-billion (ppb) level and its on-site determination remains challenging. Colorimetric test strips, though cheap and widely used, often fail to give reliable quantitative data. On the other hand, electrochemical detection is sensitive and accurate but considerably more expensive at the onset. Here, we present a study on arsenic detection in groundwater using a low-cost, open-source potentiostat based on Arduino technology. We tested different types of gold electrodes (screen-printed and microwire) with anodic stripping voltammetry (ASV), achieving low detection limits (0.7 μg L^-1^). In a study of arsenic contaminated groundwaters in Mexico, the microwire technique provides greater accuracy than test strips (reducing the median error from -50% to +2.9%) and greater precision (reducing uncertainties from ±25% to ±4.9%). Most importantly, the rate of false negatives versus the World Health Organisation’s 10 μg L^-1^ limit was reduced from 50% to 0% (N = 13 samples). Arsenic determination using open-source potentiostats may offer a low-cost option for research groups and NGOs wishing to perform arsenic analysis in-house, yielding superior quantitative data than the more widely used colorimetric test strips.

## Introduction

Arsenic detection in surface water or groundwater using cheap and portable methods remains challenging, despite the urgent need to ensure access to arsenic-free water for exposed communities and to monitor drinking water treatments better [1–5]. Arsenic is a potent carcinogen and has been associated with skin lesions such as keratoses [6], fatal internal cancers [6], miscarriage [7], and damage to the gastrointestinal, cardiovascular, neurological, genitourinary, and respiratory systems [8]. Previous studies have linked drinking arsenic-contaminated water to increased mortality, for example 220 and 140 deaths per 100,000 people per year due to bladder cancer and liver cancer respectively in the age 50–69 demographic when exposed to drinking water with >60 μg L^-1^ As [9], and 60 deaths per 100,000 people per year due to lung cancer when exposed to >35 μg L^-1^ As [10]. Worldwide, 150 million people are believed to be impacted by arsenic-contaminated drinking water [11] and it is often poor and marginalised communities who are most at risk [12].

Arsenic contamination of groundwater is a major threat to public health in Mexico [13] with an estimated 9 million people drinking from sources that exceed the World Health Organisation’s 10 μg L^-1^ guideline limit for arsenic [14]. Groundwater concentrations as high as 400–700 μg L^-1^ have been reported (and up to 74 mg L^-1^ in geothermal wells) [15]. Studies have proposed both that arsenic is released into Mexico’s aquifers through the oxidation of pyrite and dissolution of scorodite [16] and through the geothermally-induced dissolution of silicate minerals, with increasing pH leading to the desorption of adsorbed arsenic [17]. Furthermore, arsenic is often found concurrently with harmful levels of fluoride (exceeding the WHO limit of 1.5 mg L^-1^) [14]. There is an urgent need to detect contaminated water sources and provide safe alternatives, given that in many aquifers arsenic concentrations are rapidly increasing due to exploitation and over-abstraction of aquifers for irrigation [17]. A recent study of the Upper Río Laja Watershed in the state of Guanajuato, Mexico, found that the number of wells exceeding the 10 μg L^-1^ limit recommended by the WHO doubled between 1999 and 2016 [17]. Similarly, in the city of Durango, arsenic concentrations increased by around 17% between 2012 and 2016 in the North East of the city, where industrial activity and population density create high demand for groundwater resources [18]. The Mexican federal limit for arsenic in drinking water is expected to decrease from 25 ug L^-1^ to 10 ug L^-1^ in the coming years (in line with the current Mexican standard for commercial bottled water), which will require more frequent water quality monitoring with improved quantification to detect water sources that fail to meet standards [14].

Whilst the WHO currently recommends a limit of 10 μg L^-1^ (or 0.13 micromolar) arsenic [19], local standards vary significantly across the world and are in part set according to the costs and accessibility of analytical techniques with low detection limits. For instance, the Bangladesh and Mexican governments have set national standards of 50 μg L^-1^ [20] and 25 μg L^-1^ [21] respectively, whilst water utility companies in the Netherlands now aim for just 1 μg L^-1^ [22]. Many countries are likely to reduce their legislated maximum contaminant levels (MCL) for arsenic in the coming years, given that even exposure to levels of arsenic between 0 and 10 μg L^-1^ may add 4.5 lung cancer cases per 100,000 people [23].

The accurate and reliable detection of arsenic contamination is an essential part of arsenic mitigation programmes worldwide: to identify contaminated sources and safer alternatives, and to monitor the effectiveness of arsenic treatment processes [24–28]. In the case of increasing arsenic contamination in Mexico and potentially more stringent guidelines in the future, analytical techniques are needed to detect and quantify arsenic with low detection limits at a low-cost. However, the options available currently fail to meet these requirements.

State-of-the art techniques such as inductively coupled plasma mass spectrometry (ICP-MS) provide accurate and precise data, with sub-ppb detection limits (*i*.*e*. being able to detect less than one part per billion, or 1 μg L^-1^). However, these techniques have two major drawbacks. Firstly, the instrumentation is very expensive ($10 000s for atomic absorption spectroscopy (AAS) and $100 000 for ICP-MS), preventing in-house analysis by small organisations such as NGOs working in the water security sector. Such organisations often rely upon commercial analysis in private laboratories, paying high costs per sample (and potentially waiting weeks for the results). Secondly, the equipment is not portable: This is a limitation given that information on the groundwater arsenic speciation (*i*.*e*. the distribution between As(III) and As(V)) can be rapidly lost on the minutes timescale owing to oxidation and precipitation once anoxic samples are pumped to the surface [29]. On-site analysis using portable techniques is also preferable for providing quick feedback, *e*.*g*. quickly identifying arsenic-contaminated sites and safer alternatives, troubleshooting problems in arsenic treatment systems, and facilitating community engagement and education.

In contrast to ICP-MS and AAS, colorimetric test strips, typically based on the Gutzeit reaction, offer on-site arsenic detection at low-cost [30]. Here, the application of reducing agents converts As(III) and As(V) to arsine (AsH_3_) gas. Paper test strips coated with mercury bromide (HgBr) develop a yellow colour when exposed to this gas [31]. The intensity of the colour is normally compared against a calibration chart visually, providing quantitative results [27]. These field tests do not require much instrumentation (only the reaction vessel) and the test strips cost as little as half a dollar each [27]. Unfortunately, however, the results provided by these tests kits often correlates poorly with laboratory analysis, *e*.*g*. AAS [27, 28, 32, 33]. Problems include slow As(V) reduction kinetics when using zinc a the reducing agent (a negative bias in the results) and the interference of hydrogen sulphide in reducing waters (a positive bias, with test strips developing a dark grey colour) [31]. Inaccurate results can lead to harmful conclusions: the term ‘false negative’ indicates where the concentration is incorrectly reported as being below the guideline limit, and previous studies in Bangladesh have found false negative rates as high as 68% when using commercial products to assign water samples to the 50–100 μg L^-1^ range [28], and false negative rates of 5% [24] and 11% [33] versus the WHO 10 μg L^-1^ limit. Consequently, several studies have suggested a need to reconsider the application of these commercial test kits for monitoring drinking water [27], with some organisations working in Mexico now reluctant to use these products [34].

Intermediate techniques exist, providing accurate and precise data with lower instrumentation costs than AAS and ICP-MS: namely colorimetry using a digital detector, and electrochemistry. Unlike AAS and ICP-MS, both these methods can be made portable for on-site analysis, by using battery-powered or USB-powered instrumentation [1, 29, 35]. The molybdenum blue method is an aqueous-phase only colorimetric method for the determination (and speciation) of arsenic [36–38] and portable methods have been established [35]. The major limitation of this technique is a phosphate interference, which is only removed through multiple pre-treatment steps (As(V) reduction followed by PO_4_^3-^ removal through solid phase extraction and then As(III) oxidation) [36, 38]. These complications are a likely reason why the molybdenum blue method has not been established as a standard technique for measuring arsenic in natural samples [39].

We previously demonstrated on-site electrochemical detection of arsenic using anodic stripping voltammetry (ASV) and a gold microwire electrode [1, 29]. Unlike the colorimetric molybdenum blue method, this technique does not require extensive sample pre-treatments, only acidification to pH 1 for total As analysis [29]. Speciation can be achieved by varying experimental conditions (using milder deposition potentials and adding anti-oxidants) to detect As(III) only and not As(V), which is useful for understanding groundwater geochemistry [1] and monitoring advanced oxidation processes (AOPs) for water treatment [40]. The hardware (a potentiostat) is used to set the electrical potential at the working electrode, causing oxidation or reduction reactions, and to measure the current produced as a result. As with the molybdenum blue method, the consumables cost is low. However, the potentiostat is usually costly ($5 000+), and a high capital expenditure before any data is collected coupled with a lack of off-the-shelf ‘standard procedures’ [41] may be factors preventing the uptake of electrochemistry for the routine monitoring of arsenic [41].

With modern electrical components increasing in performance and decreasing in price, new hardware options exist. For instance, in 2011 Rowe et al. presented the *Cheapstat*: an open-source potentiostat for under $80 [42]. The authors demonstrated the detection of multiple analytes including ascorbic acid, ferricyanide and arsenic. However, in the case of arsenic detection, a method for the quantitative determination of arsenic concentrations was neither developed nor established. Other open-source potentiostat projects include the *PSoC-Stat* ($10) [43], *DStat* [44] ($120 [43]), and *Rodeostat* ($250), the last of which has been recommended as an option for small laboratories with limited resources to run electrochemical analysis [45]. An analytical method for determining arsenic concentrations using a low-cost, open-source potentiostat should enable smaller organisations to perform economic, in-house measurements with greater accuracy and quantitation versus the conventional colorimetric test strips.

The electrochemical detection of arsenic is most commonly achieved using ASV with a gold working electrode [46]. During the deposition step, arsenate As(V) or arsenite As(III) (depending on the deposition potential) are reduced at the working electrode surface and deposited as semi-metallic arsenic(0) [47]. By holding the deposition potential for longer times, more arsenic is concentrated at the electrode surface [41]. During the analytical stripping step, the working electrode potential is swept from negative to positive potentials, and consequently As(0) is oxidised and returns to the solution as aqueous arsenite As(III). This is anodic stripping. The amount of current generated by process is directly proportional to the concentration of arsenic originally in solution, which can be determined using the method of standard additions [47]. The technique is selective towards different metals since each metal has a specific reoxidation potential. For instance, in 0.1 M HCl the peak potential for As is at *ca*. +0.1 V whilst that of Cu is *ca*. +0.35 V. Furthermore, the technique is resistant to interference by other groundwater species including anions such as phosphate, nitrate or sulphate [1, 29]. Dissolved organic matter (DOM) can interfere with the measurement by adsorbing on the gold surface, but this matrix effect is largely removed from arsenic quantification when using the method of standard additions [1, 29]. In this work, the total inorganic arsenic concentration was quantified by detection under acidic conditions (0.1 M HCl) with a relatively low deposition potential (below -1.0 V). Such conditions immediately oxidise any As(III) present in the solution to As(V) within seconds (due to electrochemical production of the oxidant at the counter electrode) [48]. Arsenic is therefore deposited from the As(V) state and the concentration determined represents total inorganic arsenic ([total As] = [As(III)]+[As(V)]).

This work aimed to develop the first low-cost method for the electrochemical detection of arsenic using an open-source potentiostat, validated using real arsenic contaminated groundwaters in Mexico. Whilst several open-source potentiostats are now available, we chose the Rodeostat on the basis of the online support offered through IO Rodeo’s web forum. The Rodeostat is an Arduino shield, using the Teensy 3.2 development platform. The firmware supplied by the Rodeostat’s manufacturer, IO Rodeo, can generate several basic voltage: time profiles and also process set-voltage and measure-current requests. The manufacturer provides both a web-based interface and an open-source Python language interface.

We first tested the application of commercially available, cheap and mass-produced screen-printed electrodes (where working, reference and auxiliary electrodes are integrated into a single chip) given their low-cost, ease of assembly, and commercial availability worldwide. We later investigated the application of fabricated gold microwire electrodes, offering improved detection limits, shorter analysis times and greater stability. In this study, we considered total As detection rather than arsenic speciation, since for the time-being, risk assessments are generally considered on the basis of total inorganic arsenic (the sum of As(III) and As(V)). A popular commercial brand of colorimetric test strips (based on the Gutzeit reaction and using zinc as a reducing agent) were used as the benchmark low-cost technique [33]. Atomic absorption spectroscopy (AAS) analysis performed by a certified commercial analytical laboratory was used as the benchmark technique for cross-calibration [24]. Measurements were also obtained using the gold microwire electrodes and a commercial potentiostat (PalmSens2) as an additional benchmark against which to compare the performance of the Rodeostat [29].

## Materials and methods

### Chemical reagents

Distilled water from Tecnología y Control Ambiental (>1.0 MΩ-cm) was used to prepare all solutions and dilute samples. A solution of 1 M HCl was prepared from concentrated hydrochloric acid (38%, Ecolaboratorios/Karal, ACS reagent grade, CAS: 7647-01-0). A solution of 0.5 M H_2_SO_4_ was prepared from concentrated sulphuric acid (98%, Ecolaboratorios/Karal, ACS reagent grade, CAS: 7664-93-9). A stock solution of As(V) (1000 mg L^-1^) was prepared by dissolving sodium arsenate dibasic hydrate (Na_2_HAsO4.7H_2_O, Sigma, ≥98.0% purity, CAS: 10048-95-0) in distilled water, with the pH adjusted to 7.5 with the addition of a small volume of 1 M HCl. A stock solution of As(III) (1000 mg L^-1^) was prepared by dissolving arsenic trioxide (As_2_O_3_, Sigma-Aldrich, ReagentPlus®, ≥99.0% purity, CAS: 1327-53-3) in a small volume of 1.0 N NaOH (Sigma, BioReagent grade, CAS: 1310-73-2) and making up to the desired volume with distilled water. The stock solution was adjusted to pH 5 with the addition of a small volume of 1 M HCl. Arsenic standard solutions (1000 μg L^-1^) were prepared from the 1000 mg L^-1^ stock solutions by diluting with distilled water. All arsenic standard solutions were stored in the dark at 3°C to prevent changes in the speciation.

### Arsenic-contaminated water samples

Water samples were collected from the Upper Rio Laja Watershed in the state of Guanajuato, central Mexico. Thirteen samples were collected, all within 52 km of San Miguel de Allende: untreated groundwater wells (N = 8), water delivered by pipa trucks (N = 2), household water (N = 2), and groundwater that had been passed through a sediment filter followed by a carbon block filter (N = 1). A map of the sampling locations is presented in S1, S13 Figs in S1 File. No permits were required for this work, as access to the sampling sites was provided freely by the landowners (private premises). The samples were characterised using a pH meter (calibrated using buffer solutions at pH 6.86 and 9.18) and a Health Metric TDS&EC meter. Fluoride, sulphate and phosphate (orthophosphate) concentrations were determined using a Hach DR900 colorimeter and standard methods (methods 8029, 8051 and 8048). Total arsenic was determined externally using atomic absorption spectroscopy (AAS) at an accredited analytical laboratory. Samples were stored without acidification in the dark at 3°C and were warmed to room temperature prior to analysis. No precipitation was observed in the samples.

### Colorimetric detection of arsenic

Samples were analysed for arsenic using the Quick™ Arsenic Econo II test kit according to the instruction manual supplied. The sample solution (50 mL) was added to the supplied reaction bottles. The Quick™ II First Reagent was added (L-tartaric acid with iron and nickel salts) and the mixture shaken for 15 seconds. The Quick™ II Second Reagent (potassium peroxymonosulphate, potassium bisulphate, potassium sulphate, potassium peroxydisulphate and magnesium carbonate) was added and the mixture was shaken for 15 seconds. The mixture was left to rest for 2 minutes. The Quick™ II Third Reagent (powdered zinc) was then added and the bottle was immediately sealed with the plastic turret cap containing a HgBr test strip. After leaving for ten minutes, the colour on the test strip developed from the reaction of AsH_3_ with HgBr was compared against the colour chart provided. The colour bars were defined with the following concentrations: <2, 3, 5, 7, 8, 9, 10, 12, 16, 20, 25, 30, 40, 50, 80, >80, >90, >100 μg L^-1^.

### Electrochemical detection of arsenic

#### System 1: Screen-printed electrodes

Electrochemical measurements were performed using anodic stripping voltammetry (ASV) with both a Rodeostat potentiostat (from IO Rodeo) and a PalmSens 2 potentiostat (PalmSens, NL). The Rodeostat was used in two systems or configurations. In *System 1*, the Rodeostat was equipped with an integrated BVT Technologies AC1.W1.R1 screen-printed electrode (with a 0.79 mm^2^ geometric area gold working electrode, gold counter electrodes, and an Ag/AgCl solid state reference electrode). Measurements were performed using home-built software with a maximum sampling rate of 58 Hz. Total As measurements were performed at pH 1 (0.1 M HCl) according to the following procedure, optimised for these screen-printed electrodes: electrode cleaning at +0.4 V (5 seconds), deposition of As at -1.6 V (60 or 240 s depending upon the detection limit required), equilibrium/holding at -0.7 V (10 s), stripping via linear sweep voltammetry (LSV, -0.7 to +0.4 V, 0.22 V s^-1^, 5 mV potential step, 43.5 Hz sampling rate), electrode cleaning at +0.4 V (5 s). Solutions were stirred magnetically during the cleaning and deposition steps and left to settle during the holding and stripping step. After identifying an interference from hydrogen bubbles formed on the working electrode surface (see Results and discussion), the electrode was lifted from solution once every ten seconds during deposition, and once during the hold step, to remove hydrogen bubbles formed at the gold working electrode surface. Changes in the surface morphology of the working electrode component of the screen-printed electrodes were assessed using scanning electron microscopy (SEM) using a Zeiss GeminiSEM 450 with a Gemini 2 optical column.

#### System 2: Microwire electrodes, revised software and improved shielding

To improve detection limits and electrode stability, the configuration of electrodes was changed (from screen-printed to microwire electrodes) and a shielded ethernet cable was introduced to reduce electrical noise. The homemade software was also updated, increasing the maximum sampling frequency from 52.7 Hz to 1000 Hz.

In this configuration, the Rodeostat was equipped with a 30 μm diameter, 6 mm long, gold microwire electrode (geometric area 0.57 mm^2^, Informatic Component Technology, UK), iridium wire counter electrode (homemade, 150 μm diameter, ~2 cm long), and a ItalSens IS-AG/AGCL.AQ.RE.1 (Ag/AgCl/KCl (3M)) reference electrode. In contrast to the home-made gold wire electrodes used in our previous works [29, 49–51], the microwire electrodes in the present study are manufactured and all have the same length. The shielded ethernet cable reduced the electrical noise by a factor of approximately five. The software was improved, increasing the maximum sampling rate of 1000 Hz by implementing a firmware linear sweep (ramp) function from IO Rodeo. Solutions were stirred magnetically, and the working electrode was vibrated mechanically during the cleaning and deposition steps (using a 150 Hz encapsulated vibration motor (JinLong Machinery, China)) [52]. Total As measurements were performed according to the following procedure: electrode cleaning at +0.7 V (5 seconds), deposition of As at -1.3 V (20 s), holding at -0.4 V (5 s with stirring and vibration, then 2 s without), stripping via linear sweep voltammetry (LSV, -0.4 to +0.7 V, 1.2 V s^-1^, 5 mV potential step, 240 Hz sampling rate), electrode cleaning at +0.7 V (5 s). Solutions were stirred magnetically during the cleaning and deposition steps in an attempt to increase the mass transfer of arsenic towards the electrode. Total As determination using the PalmSens 2 was performed similarly, with the same 1.2 V s^-1^ sweep rate, however a 20 mV potential step and 60 Hz sampling rate was used during the linear sweep.

**Fig 1:**
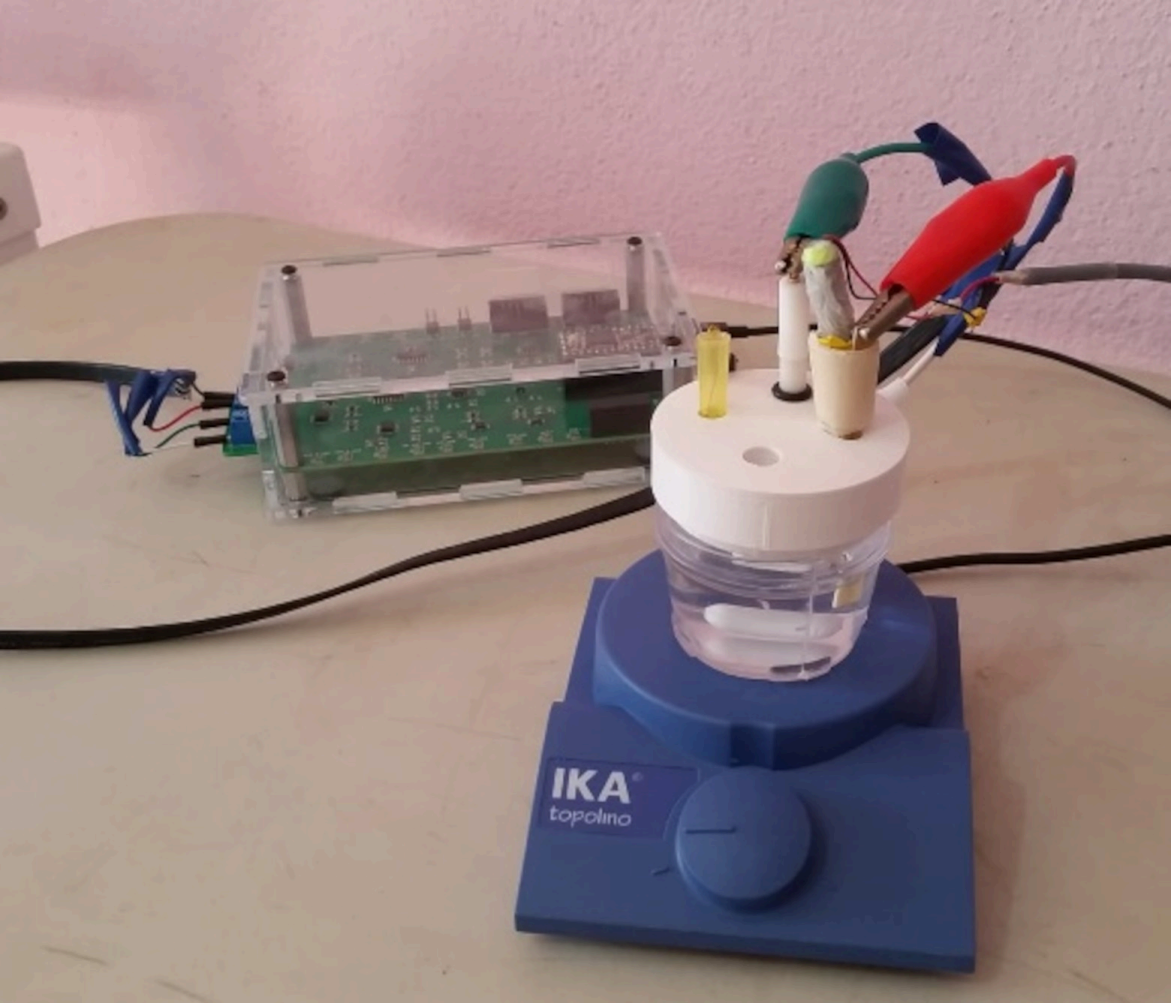
Low-cost electrochemical instrumentation for the detection of arsenic used in this work. In this case, the gold microwire set-up is presented. The Rodeostat potentiostat (in the transparent plastic housing at the back of the image) is connected to the electrodes via a shielded ethernet cable. The electrodes are mounted in a 3D printed plastic lid with an extra opening port for addition of the arsenic standard solution via pipette (to avoid knocking the electrode connections). The electrodes are (left) iridium wire counter electrode, (centre) Ag/AgCl/KCl (3M) reference electrode, and (right) gold microwire working electrode assembled with a mechanical vibrator. The 40 mL sample cell is positioned on top of a magnetic stirrer plate for mixing after addition of the sample and As(V) standard solution and possibly during the deposition stage (to increase mass transfer).

The geometric surface area of the screen-printed electrodes was 39% larger than the geometric area of the microwire electrodes (0.79 mm^2^ and 0.57 mm^2^ respectively), and the screen-printed electrodes likely have greater surface roughness than the microwire electrodes even when new (SI section 5). A photograph of the System 2 set-up is presented in Fig 1.

#### Detection of total As and determination of detection limits

The sample cell volume was 40 mL and electrochemical detection of arsenic was performed under ambient conditions, *i*.*e*. without nitrogen purging, given that many laboratories do not have access to compressed nitrogen gas, and that compressed gas is unsuitable for portable analysis.

A background scan was performed after each arsenic analytical scan, using identical voltammetric conditions to the arsenic scan, except that the deposition time was decreased to one second only. Under such short deposition times, the amount of arsenic detected is insignificant. The background scan was then subtracted from the arsenic scan prior to peak analysis to reduce the influence of charging current on the recorded voltammograms. The peak height (with a linear baseline) was used for quantification.

The limit of detection (LoD) was calculated as three times the standard deviation (σ) in the height of the arsenic stripping peak across 10 repeat scans in the presence of low levels of As(V) (LoD = 3σ). The LoD was calculated in 0.1 M HCl, using 50 and 20 μg L^-1^ As(V) for the Rodeostat with printed electrodes (60 and 240 s deposition time respectively) and using 1 μg L^-1^ As(V) for the Rodeostat with microwire electrodes (20 s deposition time).

Gold electrodes (both screen-printed and wire) were electrochemically cleaned at the start of each day and their electroactive surface areas monitored to check for changes in surface roughness. This conditioning was performed in 0.5 M H_2_SO_4_, holding the potential at -2.0 V for 30 seconds and then performing 5 repeat cyclic voltammetry (CV) scans from -0.2 to +1.5 V and back to -0.2 V (0.36 V s^-1^ and 35.7 Hz sampling frequency with the Rodeostat, and 1 V s^-1^ and 100 Hz with the PalmSens). During the positive scan (from -0.2 to 1.5 V), a gold oxide is formed at positive values; this gold oxide is reduced during the negative going scan (from 1.5 V to -0.2 V). The charge of each of those processes is directly correlated to the electroactive surface area (See S1 Fig and section 3 of the S1 File). When using the Rodeostat, peak charge (Q) in the cyclic voltammetry (CV) scans was calculated via the expression:

Q(μC)=A(μA)∙t(s) (1)

where A is the average current across the peak and t is the time taken to scan the peak, with $t\;(s)=\frac{peak\;width\;(V)}{scan\;rate\;(V\;s^{-1})}$. When using the PalmSens, the peak charge was calculated using the software.

#### Analysis of real samples using the method of standard additions

The concentration of total As in real samples was determined using the method of standard additions, to account for potential matrix effects on electrode sensitivity (with an example provided in the in S1 File). The sample was diluted to within the linear range (if necessary) and acidified to pH 1 using 1 M HCl. The sample was scanned a minimum of three times (and up to 6 times) to ensure reproducibility of the As peak. Two or three standard additions were then made using a 1000 μg L^-1^ stock solution of As(V), aiming to at least double (and at most triple) the original As peak height by the end of the experiment. A single scan was recorded after each addition using the screen-printed electrodes, due to electrode instability and long deposition times (to minimise calibration errors and improve sample throughput), whilst three repeat scans were recorded after each addition using the microwire electrodes. For each sample analysis, the original As concentration was obtained from the regression line fit to the graph of As peak height as a function of the concentration of standard added, and the associated uncertainty was calculated using Eq 2:

$$\frac{s_{y}}{\left|m\right|}\sqrt{\frac{1}{n}+\frac{{ȳ}^{2}}{m^{2}\sum {\left(x_{i}-\bar{x}\right)}^{2}}}$$ (2)

where s_y_ is the standard deviation in peak height across all data points, m is the slope, n is the number of data points, ȳ is the average peak height across all data points, x_i_ is the concentration of arsenic for data point *i*, and $\bar{x}$ is the average concentration of arsenic across all data points [53].

## Results and discussion

### Electrode stability: Screen-printed electrodes versus microwire electrodes

Cyclic voltammetry (CV) in 0.5 M H_2_SO_4_ was used to condition the electrode and also to monitor stability of the electrochemical system. Gold oxidation and reduction peaks were detected at *ca*. +1.0 V and +0.6 V respectively for the printed electrodes, versus *ca*. +1.3 and +0.9 V for the microwire system. The different peak potentials result from the different reference electrodes: the screen-printed electrodes used an integrated Ag/AgCl solid state reference electrode, whilst the microwire system was equipped with an Ag/AgCl/KCl (3M) reference electrode.

**Fig 2:**
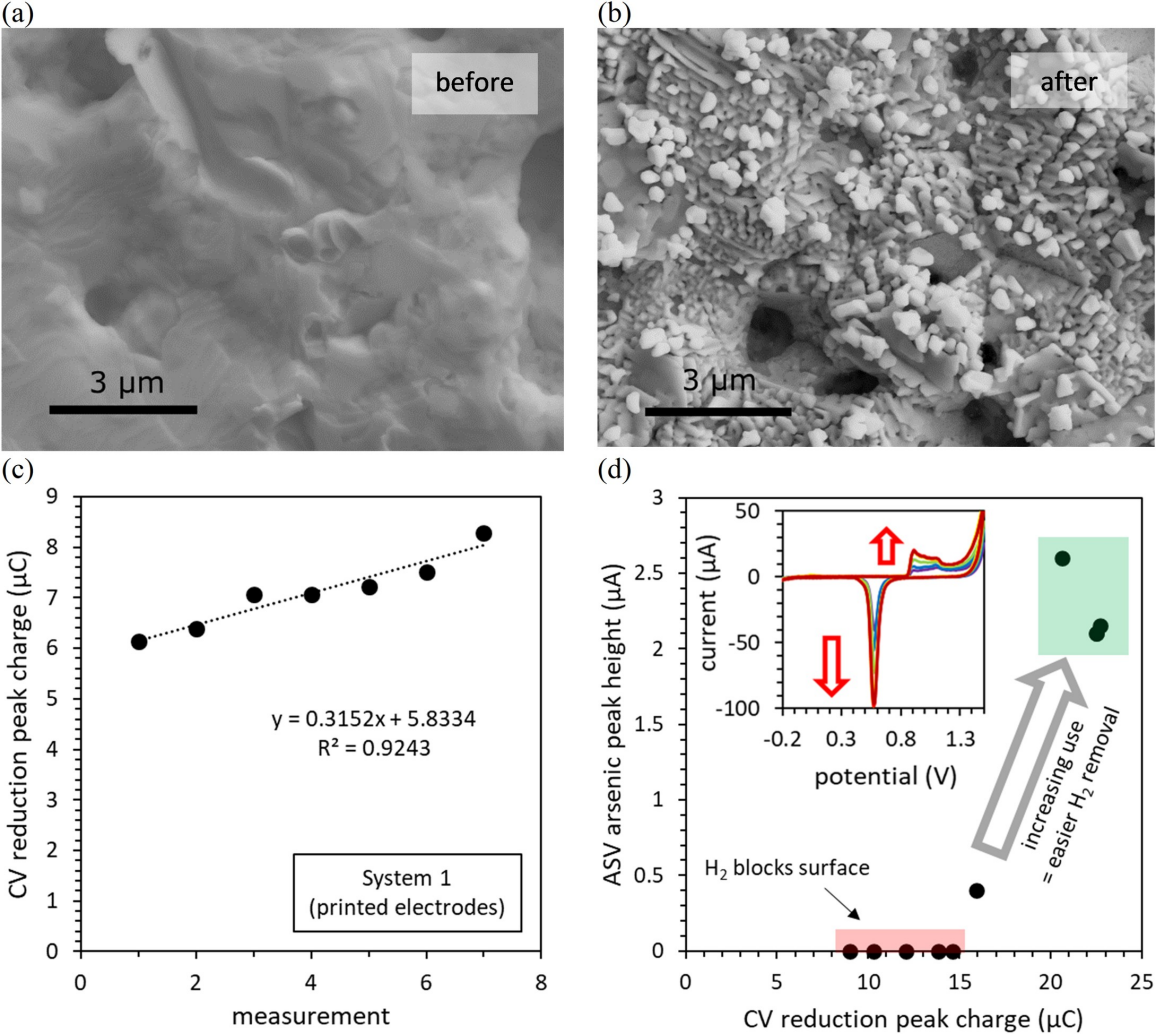
The effect of continued use on the condition and sensitivity of screen-printed electrodes in the detection of total As under acidic conditions. Increasing surface roughness of the working electrode due to the deposition of nanoparticles (a) before and (b) after continued use under acidic conditions, whereupon the charge of the reduction peak increased from 3.49 μC to 23.9 μC. (c) The increase in the intensity of the reduction peak with repeat CV measurements (including deposition at -2 V). (d) The relationship between the intensity of the reduction peak and the sensitivity of the electrode towards the detection of total As. The CV scans in clean 0.5 M H_2_SO_4_ and total As detection (167 μg L^-1^ As(V), 0.1 M HCl, 60 s deposition) were performed consecutively, repeatedly alternating between the two measurements.

Arsenic detection was first performed using the printed electrodes, where all three electrodes are deposited as thin-films on the same alumina substrate. With continued use under acidic conditions, both working and auxiliary electrodes undergo substantial changes: the colour of the working electrode changed from gold to orange on the minutes timescale whilst the auxiliary electrode dissolved within 5 hours of continued use. Scanning electron microscopy (SEM) shows that with continued use, the smooth surface of the gold working electrode becomes coated by nanoparticles with diameters typically between 200 and 400 nm (Fig 2A and 2B). These observations are consistent with the following: during the deposition step in 0.1 M HCl and/or during the conditioning procedure in 0.5 M H_2_SO_4_, a low potential is applied (-1.3 V and -2 V respectively) resulting in a high current (mainly due to the reduction of protons) at the working electrode. This current induces the oxidation/dissolution of the gold counter electrode to gold cations that are subsequently reduced and deposited as Au(0) nanoparticles at the working electrode, as seen in Fig 2B (resulting in a change of colour). Increases in the electroactive surface area of the working electrode due to these morphological changes were reflected by an increase in the charge of the reduction peak in the CV of the electrode in 0.5 M H_2_SO_4_ (Fig 2C and 2D).

Changes in the surface morphology of the gold working electrode also lead to changes in the evolution of hydrogen gas during deposition. When new, the working electrode evolves single, large hydrogen bubbles, that block arsenic deposition leading to no arsenic peak or very minor arsenic peaks (Fig 2B). These bubbles also interfere with the LSV step, unless the electrode is lifted from the solution during the holding potential. After continued use (*e*.*g*. 30 minutes) and the associated morphological changes of the working electrode surface mentioned above, smaller hydrogen bubbles are produced, which are more easily removed via magnetic stirring. Similar observations were also made at a gold microwire electrode elsewhere [54] showing that changes in the evolution of hydrogen bubbles with electrode use are not unique to the screen-printed electrodes. The easier removal of smaller bubbles from screen-printed electrodes after continued use improves arsenic deposition and thus sensitivity towards the detection of arsenic (Fig 2D). In subsequent experiments, the electrode was manually lifted from solution once every 10 seconds during the deposition step and once during the holding potential, to remove hydrogen bubbles from the working electrode surface.

In contrast, the microwire electrodes maintained consistent reduction peak charge in the CV scans (in 0.5 H_2_SO_4_) with repeat use and offered superior electrode lifetimes (S1, S3 Figs in S1 File).

### System 1: Screen-printed electrodes

**Fig 3:**
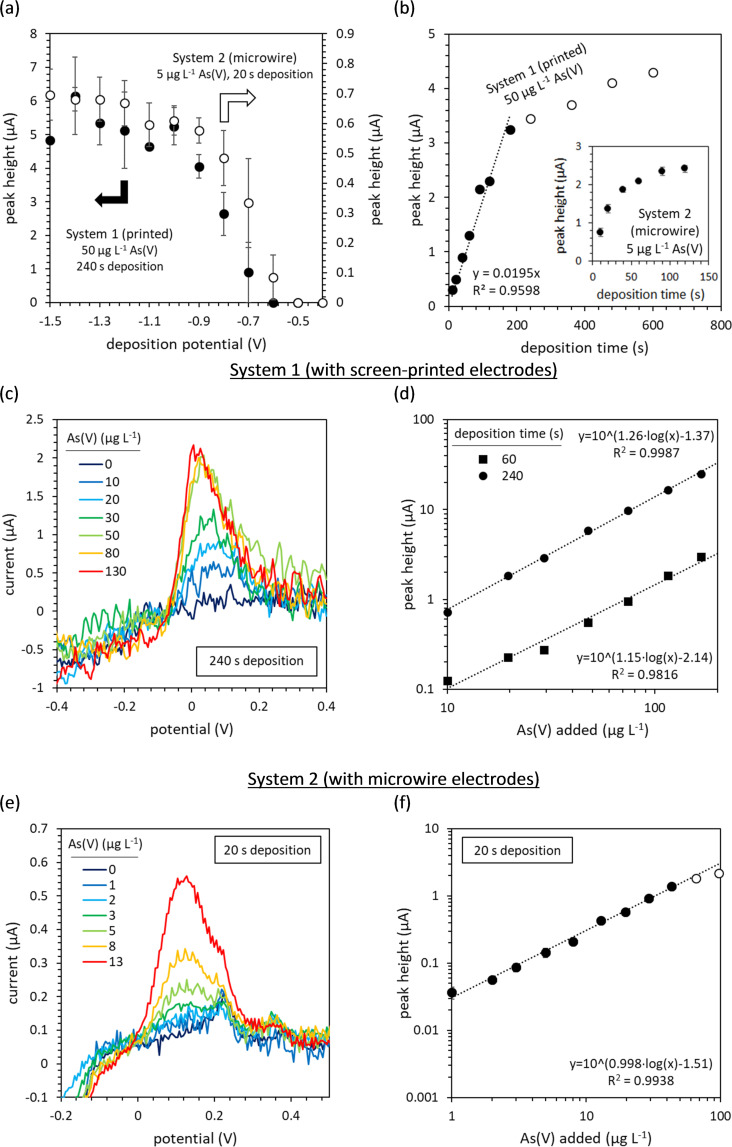
Development of an analytical method for the electrochemical determination of total As using the Rodeostat and gold working electrodes. (a) The influence of deposition potential on the height of the arsenic stripping peak (single scans and the average of two repeat experiments using screen-printed electrodes, and three repeat scans in a single experiment using microwire electrodes). (b) The effect of deposition time on the height of the arsenic stripping peak using printed electrodes (single scans using screen-printed electrodes and triplicate scans using microwire electrodes). (c) Background-subtracted voltammograms with increasing arsenic concentrations obtained using screen-printed electrodes and (d) the calibration curve highlighting the linear range. (e) Background-subtracted voltammograms with increasing arsenic concentrations obtained using microwire electrodes and (f) the calibration curve highlighting the linear range. Open shapes indicate data points outside the linear range. The data presented in (c) and (d) are the results of a single scan only (due to limited electrode stability), whilst the data presented in (e) and (f) is the average of three repeat scans. Error bars indicate the standard deviation between repeat measurements. The microwire electrode results presented in (b) were obtained using the PalmSens 2 potentiostat. All measurements were performed in 0.1 M HCl (pH 1).

Optimal conditions for the determination of total As at pH 1 using the Rodeostat and screen-printed electrodes were determined by investigating the influence of deposition potential and deposition time on the intensity of the arsenic stripping peak (using an As(V) standard solution). The arsenic peak was greatest with deposition potentials of -1.0 V or lower (Fig 3A). Arsenic was not detected with deposition potentials of -0.6 V or greater. A linear response between the deposition time and the peak height was observed as the deposition time was increased between zero and three minutes (Fig 3B). Increasing deposition times further gave diminishing returns. Consequently, 240 s deposition was used to analyse samples with low arsenic concentrations (*i*.*e*. <25 μg L^-1^), whilst 60 s was used to improve analysis times when analysing high arsenic samples (*i*.*e*. >25 μg L^-1^).

Background subtraction was performed using a scan using just 1s deposition (S1, S5 Figs in S1 File). The arsenic stripping peaks obtained on the background-subtracted voltammograms with 240 s deposition at -1.6 V are presented in Fig 3C. The limit of detection was 20 μg L^-1^ with 60s deposition, and 7 μg L^-1^ with 240s deposition. The linear range extended from the LoD up to 70 μg L^-1^ and above 200 μg L^-1^ using 240 and 60s deposition times respectively (Fig 3D).

Both methods provide detection limits below the Mexican standard for arsenic in drinking water (25 μg L^-1^) and the 240 s deposition method provides a detection limit below the WHO’s recommended limit (10 μg L^-1^) indicating that the Rodeostat equipped with low-cost screen-printed electrodes (*ca*. $1 each) can be used for arsenic monitoring.

### System 2: Microwire electrodes

Using the second configuration of the Rodeostat (with microwire working electrodes), the arsenic peak was greatest with deposition potentials of -1.2 V or lower (Fig 3A). Arsenic was not detected with deposition potentials of -0.5 V or greater. Long deposition times (and/or running many consecutive scans in the same solution) typically lead to the build-up of oxidants (*i*.*e*. the formation of chlorine at the auxiliary electrode during deposition, with a noticeable odour but no obvious colour). This was not observed when using the screen-printed electrodes. The build-up of oxidants was often accompanied by an interfering peak at -0.2 V (S1, S9 Figs in S1 File) which negatively affected the sensitivity towards arsenic. The origin of this interference is unknown but could derive from organics released from the encapsulating resin of the microwire electrode. Arsenic sensitivity was also diminished if the current at -0.4 V was less than -5 μA. These interferences were best minimised by increasing stirring rates and by coupling stirring with mechanical vibration of the working electrode (S1, S4 Figs in S1 File). With 5 μg L^-1^ As(V), the rate at which the intensity of the As stripping peak increases with increasing deposition times slowed after 20 s deposition (Fig 3B, inset). Consequently, a deposition time of 20 s was used for all sample analysis, whereby the interference of oxidants was minimal.

Besides a peak at +0.35 V corresponding to copper [49], the As peak often showed a shoulder at +0.22 V (Fig 3E). Despite occasionally being present in the blank electrolyte (0.1 M HCl), this peak typically increased with the addition of arsenic, and increased with longer deposition times. A similar shoulder on the As peak has been observed previously in freshwater [49], seawater [50] and also in high chloride low pH solution at a Au foil electrode [55], and may be due to differences in the stripping of As atoms from the different surface planes of polycrystalline gold.

Nevertheless, the second Rodeostat system with microwire electrodes achieved significantly lower detection limits than System 1 (screen-printed electrodes) with shorter deposition times: the limit of detection obtained in 0.1 M HCl was 0.7 μg L^-1^ with 20 s deposition (Fig 3E). Furthermore, with short deposition times and greater electrode stability, three repeat scans could be made between each standard addition, maintaining good sample throughput, in the subsequent analysis of real samples. The linear range extended from the LoD up to 20–40 μg L^-1^ (Fig 3F).

### Characterisation of drinking water samples

High concentrations of 130 μg L^-1^ arsenic have been detected in the Upper Rio Laja Watershed previously [17]. A partial characterisation of the water samples collected from this arsenic-contaminated region of Mexico in the current study is presented in Table 1 (with a map of sampling locations presented in S1, S13 Figs in S1 File). The pH varied between 7.1 and 8.6. High arsenic concentrations (>25 μg L^-1^) were correlated with alkaline pH (53 and 74 μg L^-1^ As at pH 8.2 and 8.6 respectively) (S1, S15 Figs in S1 File). This is in-line with the arsenic release mechanism proposed in a previous study of the same aquifer in central Mexico, wherein the introduction of geothermal waters promotes the dissolution of silicate minerals, increasing the pH, and thus releasing arsenic oxyanions through the conversion of adsorption surface sites from positive to negative charge [17]. High arsenic concentrations (>25 μg L^-1^) were also associated with high concentrations of sulphate (>50 mg L^-1^), perhaps released through the same mechanism (S1, S15 Figs in S1 File). No strong correlations between arsenic concentrations and fluoride or phosphate were observed (S1, S15 Figs in S1 File). All 8 untreated groundwaters contained more than the WHO’s recommended limit of 10 μg L^-1^ As, and two samples contained more than the 25 μg L^-1^ Mexican standard. The groundwater treated by a sediment filter and then a carbon block filter contained <10 μg L^-1^ As (whilst another sample located from an untreated source ~100 m away contained 12.2 μg L^-1^). Two samples were from drinking water delivered by trucks, to a community whose groundwater well had collapsed, and one of these samples exceeded the WHO 10 μg L^-1^ limit. The kitchen tap sample had been treated with reverse osmosis (RO), showing low total dissolved solids (TDS, 21 mg L^-1^), low fluoride (0.16 mg L^-1^), and low total As (below the limit of detection using AAS).

**Table 1:** Characterisation of real water samples used for validating the Arduino electrochemical method for total As detection.

sample	source	location	pH	TDS (mg L^-1^)	SO_4_^2-^ (mg L^-1^)	F^-^ (mg L^-1^)	PO_4_^3-^ (μg L^-1^)	As (μg L^-1^)				
								AAS	colorimetric test strips	PalmSens/microwire electrodes	Rodeostat/SPE (System 1)	Rodeostat/microwire (System 2)
1	groundwater from well	El Fraile, San Miguel de Allende	7.58	221	12.5	1.98	66	10.0	5±2	10.4±0.9	-	10.2±0.5
2	groundwater from well	Agustín González, San Miguel de Allende	7.68	158	10.8	3.76	<LoD	11.2	5±2	11.0±1.3	7.9±0.8	13.3±0.7
3	groundwater from well	La Palma, San Miguel de Allende	7.28	178	25.2	1.74	30	12.6	7±1.5	14.0±1.9	14.2±2.6	15.8±0.5
4	groundwater from well	Ex-Hacienda de Jesus, San Diego de la Unión	8.20	266	54.3	14.0	<LoD	52.9	57.7±6.4	89.4±13.6	79.5±8.0	69.9±1.7
5	groundwater from well	Terreros de la Concepción, San Luis de la Paz	8.58	282	54.0	12.0	108	73.6	32±8	74.9±6.5	51.0±10.0	70.9±8.6
6	groundwater from well	Atotonilco site #1, San Miguel de Allende	7.53	194	29.5	1.83	46	12.2	8.6±3.4	18.0±1.5	23.8±4.1	12.0±1.2
7	groundwater from well	Atotonilco site #2, San Miguel de Allende	-	-	12.8	*	<LoD	12.2	12±3	14.6±1.8	15.6±1	14.2±0.7
8	groundwater from well	Atotonilco site #3, San Miguel de Allende	7.19	228	27.3	1.66	40	12.2	4.9±2.5	13.9±1.3	9.9±1.1	11.4±0.6
9	groundwater with carbon filter	Atotonilco site #4, San Miguel de Allende	7.54	422	63.6	2.54	166	8.8	3±1.5	5.8±1.1	8.5±2.2	6.7±1.4
11	bathroom tap	Colonia Olimpo, San Miguel de Allende	7.95	185	23.5	2.16	54	18.2	9±1	17.4±2.1	17.9±2.8	20.6±1.1
10	kitchen tap	Colonia Olimpo, San Miguel de Allende	7.3	21	<LoD	0.16	<LoD	<LoD	1±1	0.7±0.2	<LoD	1.0±0.1
12	water delivery truck (pipa)	El Fraile pipa #1, San Miguel de Allende	7.55	184	20.0	3.42	20	6.4	8±1	6.2±0.4	9.4±1.5	5.1±0.3
13	water delivery truck (pipa)	El Fraile pipa #2, San Miguel de Allende	7.14	170	20.8	1.08	76	14.0	7±1.5	15.0±1.8	17.7±3.6	14.5±0.7

All samples were collected within 52 km of San Miguel de Allende. Four separate wells were sampled in Atotonilco whilst the two samples from Colonia Olimpo were collected from the same house. Samples were collected from two pipas (water delivery trucks) in El Fraile, however the original locations of these water samples are unknown. A map of all sampling locations is presented in S1 File (S1, S13 Figs in S1 File). Acronyms are SPE (screen-printed electrode), TDS (total dissolved solids), AAS (atomic absorption spectroscopy) and LoD (limit of detection). Detection limits were 0.02 mg L^-1^ for fluoride, 6.7 mg L^-1^ for SO_4_^2-^ and 26 μg L^-1^ for PO4^3-^ using the Hach colorimeter. Total As detection limits were 1 μg L^-1^ using AAS, 0.34 μg L^-1^ using the PalmSens, 7 μg L^-1^ using Rodeostat System 1, and 0.79 μg L^-1^ using Rodeostat System 2. Decimal places in the colorimetric test strip results correspond to samples that were diluted with deionised water prior to analysis.

* Fluoride was not measured for this sample; however historical data gives fluoride concentrations of 1.5–2.0 mg L^-1^ for this well. The uncertainties given for colorimetric measurements correspond to the width of the colour band of the calibration chart, whilst the uncertainties given for electrochemical measurements correspond to the error in the linear regression applied to the standard addition calibration curve, calculated using Eq (2).

### Superior quantification of arsenic contents in real samples using Arduino electrochemistry

The two methods developed for the Rodeostat were used to analyse these real samples (System 1 with screen-printed electrodes and System 2 with microwire electrodes). Samples were also analysed using colorimetric test strips (based on the Gutzeit reaction) as the benchmark low-cost technique. Electrochemical analysis made using the PalmSens 2 potentiostat equipped with the microwire electrodes was used to assess the potential of the low-cost open-source potentiostat versus low-noise commercial hardware. The results obtained using these different analytical methods were compared to the results of AAS (Table 1).

**Fig 4:**
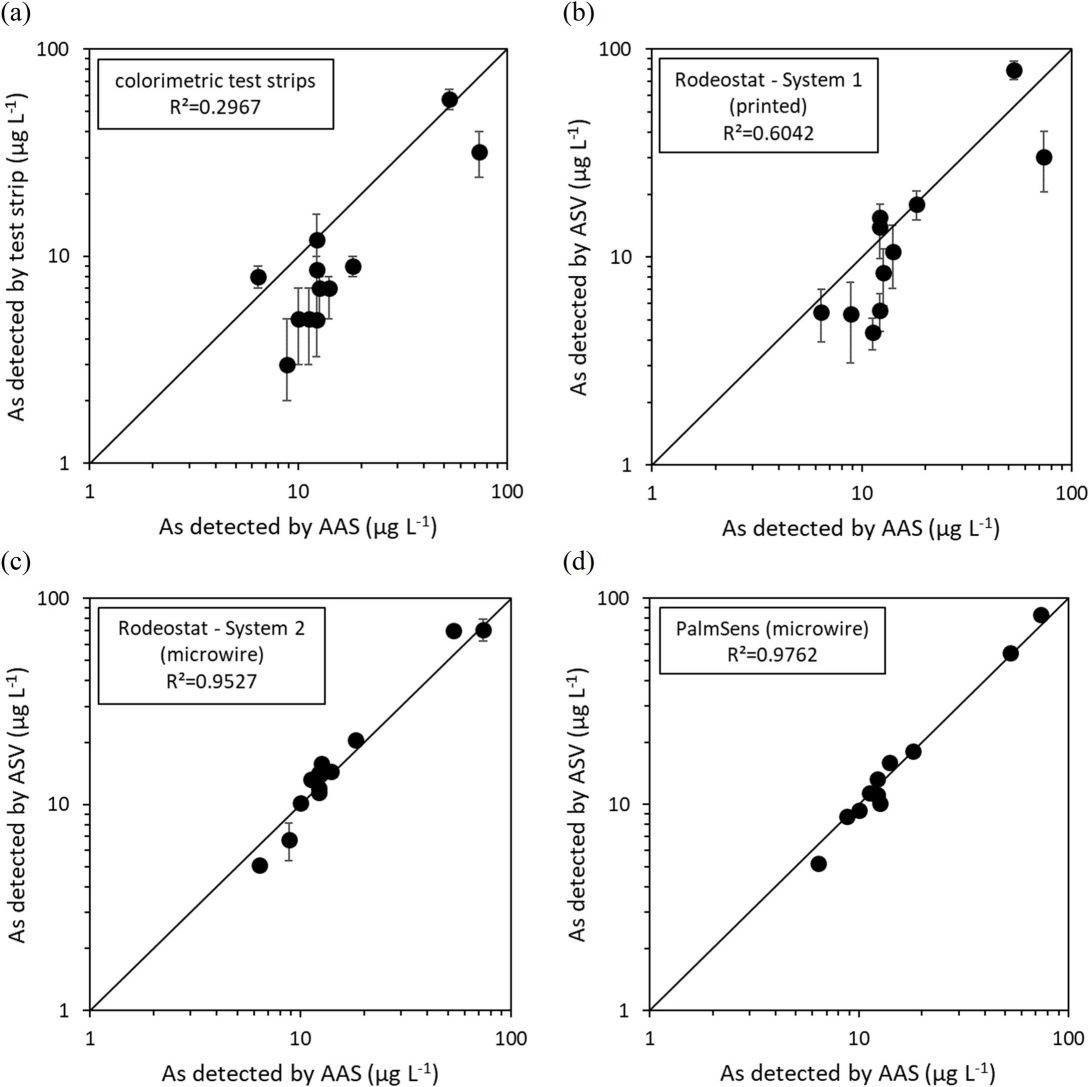
Accuracy of [total As] determination in naturally arsenic contaminated water samples, cross-calibrated against atomic absorption spectroscopy (AAS) as the benchmark technique. Goodness of fit (R^2^) values were calculated using the formula $R^{2}=1-\left(\frac{\sum_{i=1}^{n}{\left({log\;([total\;As])}_{detected,i}-{log\;([total\;As])}_{AAS,i}\right)}^{2}}{\frac{1}{n}\sum_{i=1}^{n}{\left[total\;As\right]}_{detected,i}}\right)$. Error bars indicate the uncertainty of each measurement. For the field test measurements, uncertainties were taken from the colour chart, whilst for electrochemical measurements, uncertainties were determined from the standard error in the slope and y-intercept of the linear regression obtained from the internal calibration made using the method of standard additions.

The results obtained with the colorimetric test strips typically underestimated the concentration of arsenic determined by AAS with a median error of -50% (Fig 4A). This significant underestimation was confirmed using a Wilcoxon signed rank test (p<0.05). Consequently, the goodness of fit to the ideal one-to-one line is poor, with R^2^ = 0.2967 (Fig 4A). Similar results were observed in a previous study of water samples collected from this river basin, with a typical error of around -75% [34]. As an indicator of precision, the median uncertainty in the total As measurement was ±25%. Whilst the Gutzeit method is known to suffer from a sulphide interference, arsenic contaminated groundwaters in this region of Mexico are typically oxic [17], making this interference unlikely. Furthermore, this interference would create a positive bias to the results, rather than the negative bias observed. Another possible cause for the systematic error is reaction kinetics, as the reduction of As(V) to arsenic gas (AsH_3_) using zinc (as per this test kit) is slower than the reduction of As(III) to AsH_3_. One literature source suggests that the reduction of arsenic to arsine is catalysed by the presence of chloride (Cl^-^) [31], however the groundwaters of the Rio Laja river basin contain carbonate (HCO_3_^-^) as the primary anion (4.0 mM HCO_3_^-^ versus 0.27 mM Cl^-^) [17]. Consequently, the slow kinetics of As(V) reduction in the Mexico groundwater matrix (versus the groundwater matrices used to prepare the colour chart supplied by the manufacturer for calibration) most likely explain the systematic underestimation in total As concentrations.

The Rodeostat System 1 configuration provided a much greater agreement with the AAS results, with a smaller median error of +5.5% and R^2^ = 0.6042, indicating superior accuracy (Fig 4B). No statistically significant difference between the two sets of data was found (using the Wilcoxon signed rank two-tailed test, p>0.05). The median uncertainty is similar to that of the colorimetric test strips (±28% versus ±25%). The System 2 configuration further improves the agreement with AAS, giving a median error of +2.9%, R^2^ = 0.9527 and no statistically significant difference versus AAS at p<0.05 (Fig 4C). The precision was also improved, with the median uncertainty decreasing to ±4.9%. These improvements are due to the lower detection limits, improved electrode stability, and faster ASV measurements allowing for repeat scans when using the microwire electrode. Finally, the Rodeostat was compared against a commercial potentiostat (PalmSens 2) using a 100 Hz sampling frequency, potential step size of 20 mV and a 1.2 V s^-1^ scan rate. Measurements taken using the PalmSens 2 gave the greatest agreement with AAS (a median error of -0.3%, R^2^ = 0.9762, and no statistically significant difference versus AAS at p<0.05, Fig 4D). This method gave the lowest uncertainty, with a median value of 2.7%.

**Fig 5:**
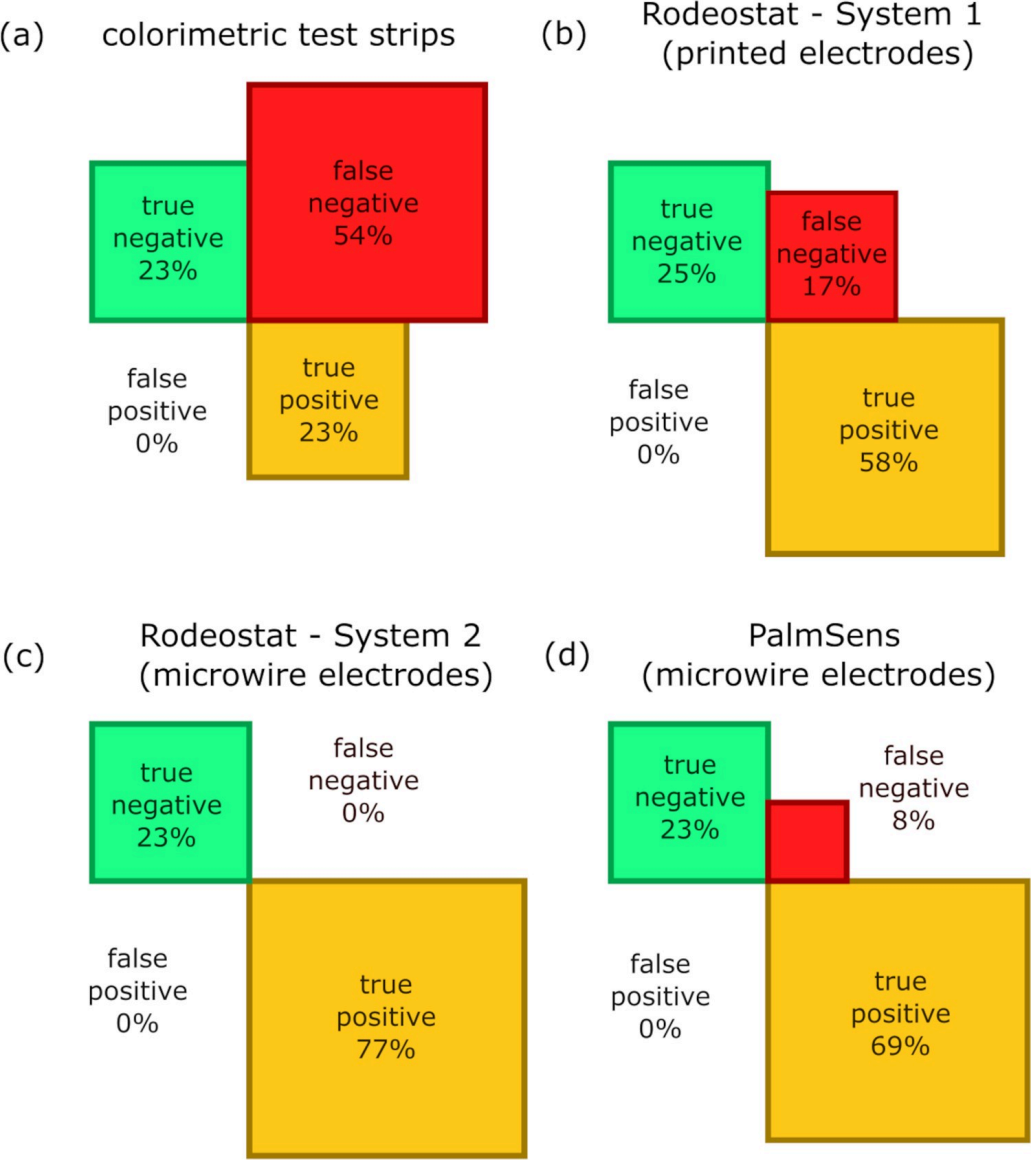
True negative, true positive and false negative rates calculated for each analytical technique (N = 13). ‘Contaminated’ water samples were defined as those samples where >10 μg L^-1^ total As was detected using atomic absorption spectroscopy (AAS). No false positive results were detected using any of the analytical techniques.

Considering water to be ‘safe’ when arsenic concentrations are below the WHO’s recommended 10 μg L^-1^ limit, and ‘unsafe’ when above this limit, the colorimetric test strips gave a very high false negative rate of 53.9% (N = 13, Fig 5A). Unlike the colorimetric test strips, the System 1 configuration of the Rodeostat with screen-printed electrodes does not show a systematic underestimation of arsenic concentrations, and thus has a lower false negative rate of 16.7% (N = 12, Fig 5B). The increased precision of the System 2 configuration further reduced the false negative rate to 0% within these 13 samples (Fig 5C). The high selectivity of testing samples against the 10 μg L^-1^ limit using the Rodeostat equipped with microwire electrodes compares favourably against the PalmSens benchmark system (Fig 5D).

## Discussion

A comparison of the analytical methods used in this study is provided in Table 2. The Rodeostat provided superior quantitation than the colorimetric test strips in these water samples, with improved accuracy, a lower rate of false negatives, and smaller uncertainties, at a similar initial cost. Importantly however, most of the costs for electrochemical detection using the Rodeostat were for instrumentation, and this method would have lower running costs in the long term versus this particular commercial test strip product. (It is worth noting that the price of other test strip products can be as low as $0.60 per sample [27]).

**Table 2:** Comparison of analytical methods used in this study.

	Atomic absorption spectroscopy (AAS)	Colorimetric test strips	Commercial potentiostat and microwire electrodes	Rodeostat/SPE (System 1)	Rodeostat/microwire (System 2)
Hardware cost ($)	$10 000’s	0	~2 000–10 000 ^a^	250	~300
Running costs ($ sample^-1^)	~$0.5–7 ^b^	3 ^c^	~1.4 ^d^	~0.4 ^e^	~1.4 ^d^
Detection Limit (LoD, μg L^-1^)	1	2	0.34 (20 s deposition)	7 (240 s deposition)	0.79 (20 s deposition)
				20 (60 s deposition)	
Linear range	-	<50 ^f^	<20	<70 (240 s deposition)	<20–40 (20 s deposition)
				>200 (60 s deposition)	
Operator time (min sample^-1^)	-	15	25 ^g^	60 (240 s deposition) ^h^	30 ^g^
				45 (60 s deposition) ^h^	
Median error versus AAS (%) (N = 12)	- ^i^	-50±30	-0±11	-29±43	3±17
R^2^ versus AAS (linear, not log)	- ^i^	0.2514	0.9809	0.4302	0.9436
Median uncertainty (%) (N = 12)	- ^i^	25±14	2.7±1.7	28±11	4.9±5.2
False negative rate at 10 μg L^-1^ (%) (N = 13)	- ^i^	54	8	25	0

We assume that the screen-printed electrodes can measure 5 samples before malfunction, whilst the microwire electrodes can measure 50 samples before malfunction. Values of the median error and median uncertainty were calculated using N = 12 data points, discounting the sample that was below the detection limit when analysed using AAS. The false negative rate was calculated using all N = 13 samples. Improvements in the detection limit when changing from the Rodeostat System 1 to the Rodeostat System 2 were not only due to the different As deposition rates, but also due to improvements in the software offering faster sampling rates, and improved electrical shielding, as discussed elsewhere in this work. The operator time includes acidification of samples and the spiking of samples with standards, but not initial preparation time (e.g., the preparation of standard solutions).

^a^ The PalmSens 2 used in this work was >10 years old and newer products achieve faster sampling rates. Up to $10,000 for commercial potentiostats [56].

^b^ approximate range from the literature [57].

^c^ $300 USD for 100 test strips at the time of writing, however cheaper test strip products are available, starting at around $0.60 per sample [27].

^d^ The microwire electrodes used in this study cost approximately $70 to fabricate and can measure approximately 50 samples under the acidic and oltametric conditions used in this study.

^e^ The screen-printed electrodes used in this study are priced at 0.76 euro cents per unit, and last approximately five sample measurements before the counter electrode is compromised by dissolution under the acidic and oltametric conditions used in this study.

^f^ The manufacturer’s calibration chart states that the test strips are quantitative up to this concentration.

^g^ Triplicate measurements after each addition of sample or standard.

^h^ Duplicate measurements after each addition of sample or standard.

^I^ Assumed to give the ‘true’ result with perfect accuracy.

The original homemade software was limited to 52.7 Hz sampling rates, due to the time needed to send instructions to change the potential and receive data on the current. The use of a buffering function was needed to obtain the maximum 1000 Hz sampling rate of the Rodeostat. Given the significant noise in the Rodeostat voltammograms (S1, S5 Figs in S1 File), high sampling rates with a small potential step to generate a large number of data points covering the As peak is desirable. This will likely be true for other low-cost, open-source potentiostats. The second Rodeostat system, with gold microwire electrodes, achieved a detection limit in 0.1 M HCl of 0.7 μg L^-1^ with 20 s deposition (Fig 3B). Although this is 10 times higher than previously reported for similar deposition times [49], these other studies have used more expensive, low-noise potentiostats.

The main disadvantage of electrochemical detection as performed in this study is the time required from the operator. With the microwire electrodes, approximately 30 minutes were required to measure each sample (*i*.*e*., six repeat scans of the sample plus three standard additions with three repeat scans for a total of 15 measurements). Sample throughput decreases further when using the first Rodeostat system with screen-printed electrodes due to the long deposition times necessary to produce a peak above the detection limit. The sample analysis time could be decreased by a factor of three (*i*.*e*., 10 minutes per sample using microwire electrodes) by using a pre-determined external calibration curve, thus removing the need for standard additions. However, this approach is only valid where the electrode sensitivity is sufficiently stable for different samples with different matrix compositions. Currently, this cannot be achieved using screen-printed electrodes under acidic conditions due to the significant changes in electrode sensitivity as the surface morphology of the electrode changes with continued use. The slope of the standard addition calibration curve (μA (μg L^-1^)^-1^) varied considerably during analysis of the 14 natural samples (standard deviation, σ = 39% with 240 s deposition, S1, S16 Figs in S1 File). The sensitivity of the microwire electrodes also varied considerably during the analysis of the 13 samples (standard deviation, σ = 18–27%, S1, S16 Figs in S1 File), potentially both due to matrix effects, such as the adsorption of organics, and memory effects. This variation in electrode sensitivity shows that the method of standard additions is needed to calibrate results when assessing different groundwaters. Decreasing the detection limit further to allow for extensive dilution of the sample in a well-known matrix would possibly minimise interferences enough for an external calibration procedure to be used. This needs to be tested.

Adapting the technique to neutral pH may also improve stability, avoiding dissolution of the screen-printed electrodes and the potential leaching of the insulating resin of the microwire electrodes. Arsenate detection at neutral pH is possible if carried out in presence of manganese [51]. Neutral pH detection is especially desirable for the screen-printed electrodes, as this would remove the interference of hydrogen bubbles formed on the working electrode during deposition. Neutral pH detection is also likely to reduce operating costs by improving electrode lifetimes and minimise exposure of the operator to chlorine gas.

Unlike AAS, both colorimetric test strips and electrochemistry can be used for portable/on-site analysis. The Rodeostat is powered through a USB connection to a laptop computer. For analysis in the field, our current instrumentation would ideally be modified to also use USB or battery power for the stirring or vibration. On-site analysis is especially important for determining arsenic speciation, given the time-limited stability of As(III) (found in reducing groundwaters) once exposed to air [29]. Whilst the aim of the current study was to develop a method for total As determination using the Rodeostat, arsenic speciation with selective determination of As(III) should also be achievable, using methods similar to those previously reported [1, 29].

## Conclusions

This study developed a low-cost method for the determination of total arsenic using electrochemistry and a potentiostat based upon Arduino technology. This method provides superior quantitation of arsenic versus the much more widely used colorimetric test strips, at a similar cost. When the potentiostat is equipped with microwire electrodes, a detection limit of 0.7 μg L^-1^ is achieved for a short 20 s deposition time. This sub-ppb detection limit is comparable to commercial analysis using atomic absorption spectroscopy (AAS). Unlike the colorimetric test strips used in this study, which use an external calibration chart, electrochemical detection with the method of standard additions has no systematic error and significantly reduces the risk of reporting false negative results.

This study demonstrates a promising proof-of-concept for low-cost electrochemistry using open-source potentiostats. This technique has the potential to improve arsenic quantitation both in resource-limited laboratories and in field studies where portable instrumentation is needed. Future work should develop this low-cost arsenic detection technique for the non-expert user, *e*.*g*. by moving to neutral pH, and by improving the user-friendliness of the homemade software for data analysis. Such low-cost monitoring devices may find applications in the monitoring of other trace metals (e.g. Cu) and may also be well suited for educational purposes, with the possibility of raising awareness of arsenic and metal environmental contamination through an experimental approach.

## Supporting information

S1 File Supplementary figures and tables to compliment the main text. (PDF)

S2 File Home-made software developed for this paper, electrode cleaning and total arsenic detection procedure files, peak analysis and standard addition Microsoft Excel spreadsheet templates, and user guides. (ZIP)

S1 Data Data sets for publication. (XLSX)
