# Supplementary material for: Low-cost electrochemical detection of arsenic in the groundwater of Guanajuato state, central Mexico using an open-source potentiostat
Source: PLoS One. 2022 Jan 19;17(1):e0262124. doi: 10.1371/journal.pone.0262124 (PMC8769315; doi:10.1371/journal.pone.0262124)
Supplement: S1 File — (PDF) [file pone.0262124.s001.pdf]

Low-cost electrochemical detection of arsenic in the groundwater of Guanajuato State, Central Mexico using an open-source potentiostat

Jay C. Bullen[a]*, Lawrence N. Dworsky[b], Martijn Eikelboom[b,c], Matthieu Carriere[b], Alexandra Alvarez[b], Pascal Salaün[c]*

[a] Department of Earth Science and Engineering, Faculty of Engineering, Imperial College London, London, UK, SW7 2BX

[b] Caminos de Agua, José María Correa 23A, Colonia Santa Cecilia, 37727 San Miguel de Allende, Guanajuato, Mexico

[c] Department of Earth, Ocean and Ecological Sciences, School of Environmental Sciences, University of Liverpool, Liverpool, UK, L69 3BX

* Corresponding author:

Email address: j.bullen16@imperial.ac.uk (Jay Bullen), pascal.salaun@liverpool.ac.uk (Pascal Salaün)

Table of Contents

1. Equipment list for electrochemical arsenic detection 2
2. Software 3
3. Comparing the stability of screen-printed and microwire electrodes using cyclic voltammetry in 0.5 M H_2SO_4 4
4. The importance of stirring and/or vibration 7
5. Quality of voltammograms and noise in the detection of total As 9
6. PalmSens 2-microwire electrode system 11
7. Interference during the detection of total As using microwire electrodes with long deposition times and repeat scans 12
8. Arsenic peak stability 13
9. Equal sensitivity to As(III) and As(V) 14
10. Measuring arsenic using the method of standard additions 15
11. Characterisation of natural water samples 16
12. Electrode sensitivity in natural matrices 18
References 20

1. Equipment list for electrochemical arsenic detection

The following table highlights the essential parts necessary to perform total As detection under acidic conditions using the Rodeostat as per this study. The user can choose between the screen-printed electrodes (parts given in red) or the microwire system (parts given in blue). Auxiliary and reference electrodes have been listed as hardware, as no replacements were needed during this experimental study. The tables assumes that the user has access to typical equipment such as typical chemistry glassware, namely measuring cylinders, and clean opaque plastic bottles for storing arsenic solutions and samples (ideally refrigerated prior to analysis).

*Table S1: List of equipment needed for total As detection under acidic conditions using the Rodeostat, as performed in this study. Suppliers, prices and references are based on those used in this study, with commercial alternatives provided in some instances, and references given to papers outlining a procedure to fabricate microwire electrodes by hand, using gold microwire, conducting silver epoxy and pipette tips. Prices are converted to USD, however most items were purchased in the UK or in Mexico. *Alternate pipette brands exist, costing as little as one fifth of the price listed here, however we have not tested their quality.*

Part	Description	Price (USD)	Supplier and model	Reference
		Hardware		
Potentiostat	To set the potential and record the current	$250	IO Rodeo Rodeostat version 0.2	https://iorodeo.com/products/potentiostat-shield?variant=39282821595251
Shielded ethernet cable	To connect the electrodes to the potentiostat with low electrical noise from the mains electricity	$3	-	www.amazon.com
Magnetic stirrer plate and stirrer bar	To mix the solution during deposition	$89 or $33	IKA Topolino or INTLLAB	www.amazon.com
		and/or		
Encapsulated vibration motor	To mix the solution during deposition	$3	JinLong Machinery Z6DL2H0125215	https://www.vibration-motor.com/products/cylindrical-vibrator-motors/encapsulated-vibration-motors
Homemade iridium wire auxiliary electrode	Auxiliary electrode	$165 for commercial product or $303 (wire to make ~10 electrodes)	ItalSens IS-PT.W.CE.1 or Fisher Scientific 11349418	https://www.palmsens.com/product/counter-electrode-made-platinum-wire/ or https://www.fishersci.co.uk/shop/products/platinum-wire-0-5mm-0-02-in-dia-annealed-99-95-metals-basis/11349418
Ag/AgCl/KCl (3M) reference electrode	To provide a reference potential	$47	PalmSens IS-AG/AGCL.AQ.RE.1	https://www.palmsens.com/product/reference-electrode-with-agagcl-in-aqueous-kcl/
		Consumables (electrodes)		
Screen-printed electrode	Working, auxiliary and reference electrodes all	$2	BVT Technologies AC1.W1.R1	https://www.palmsens.com/product/bvt-electrochemical-sensor/

	integrated on a single chip			
Gold microwire electrode	Working electrode only	$55-70 or $unknown or ~$2	non-commercial or Metrohm scTRACE Gold or Home-made	https://www.ict-ltd.co.uk or https://www.metrohm.com/en-gb/products-overview/61258000 or published procedure [1] [2] [3] [4]
Consumables (reagents)				
As standard solution (1 mg L^{-1})	Calibration standard for the method of standard additions	$37 (250 mL)	Supelco 39436-250ML	mexico@sial.com
HCl (1 M or greater)	Acidification of samples to improve As detection	-	Karal, Mexico	-
H_2SO_4 (0.5 M or greater)	For CV scans to condition electrodes and check performance.	-	Karal, Mexico	-
Distilled or deionised water	To dilute stock solutions	$6 (20 L)	Tecnología y control	-
KCl (3M or greater)	To refill Ag/AgCl/KCl reference electrode	$22 (250 mL)	Supelco 60137-250ML	mexico@sial.com
Other				
Pipette	The pipette will ideally deliver volumes smaller than 100 µL (for the method of standard additions)	$347 *	Eppendorf 3124000075	https://www.coleparmer.co.uk/i/eppendorf-3124000075-single-channel-pipette-yellow-fixed-100-ul/2450525
Pipette tips	To fit the pipette	<$10 for 500	-	-

2. Software

In this work, we used a home-made program built in Python to run the arsenic detection procedure. First, Python must be installed on the user's computer, and the Rodeostat assigned a suitable COM port number (*i.e.* 3). The arsenic test program is initiated (the *.py* file) and different procedure files (*.ast* files) are loaded to run the procedures for electrode cleaning and for arsenic detection. The results can then be saved as a .dat file and opened using our data processing templates in Microsoft Excel. Full details are provided in the documentation. Please see the attached files in the supporting information for:

- Our user guide
- The Python program

- .ast files with programs for electrode conditioning, total As detection (with 1 scan) and total As detection (with 3 repeat scans).
- A Microsoft Excel template for analysing the results and determining the peak height
- A Microsoft Excel template for calculating the original concentration of the analyte within the sample using the method of standard additions.

The .ast files can be edited to change the potentiostat operating parameters (*e.g.* deposition time, deposition potential, scan rate, and to add extra hold or cleaning steps).

We aim to provide updated versions of the open-source software with new features on the Caminos de Agua website (caminosdeagua.org) and we are currently developing a program for automatic peak search, baseline detection, and peak intensity quantification.

3. Comparing the stability of screen-printed and microwire electrodes using cyclic voltammetry in 0.5 M H_2SO_4

A comparison of the typical cyclic voltammograms obtained using screen-printed and microwire electrodes is presented in Figure S1. Voltammograms taken using the Rodeostat often feature significant positive current at the beginning of the anodic scan (left-hand side) and at the beginning of each cathodic scan (Figure S1b). The positive current at the beginning of the anodic scan is explained by the sudden change from the conditioning potential (-2.0 V) to the first potential of the anodic sweep (-0.2 V). The reason for increase in the positive current when switching between anodic and cathodic scans that is often observed is unknown but may be explained by the Rodeostat software erroneously applying a temporary positive potential when initiating the linear sweep.

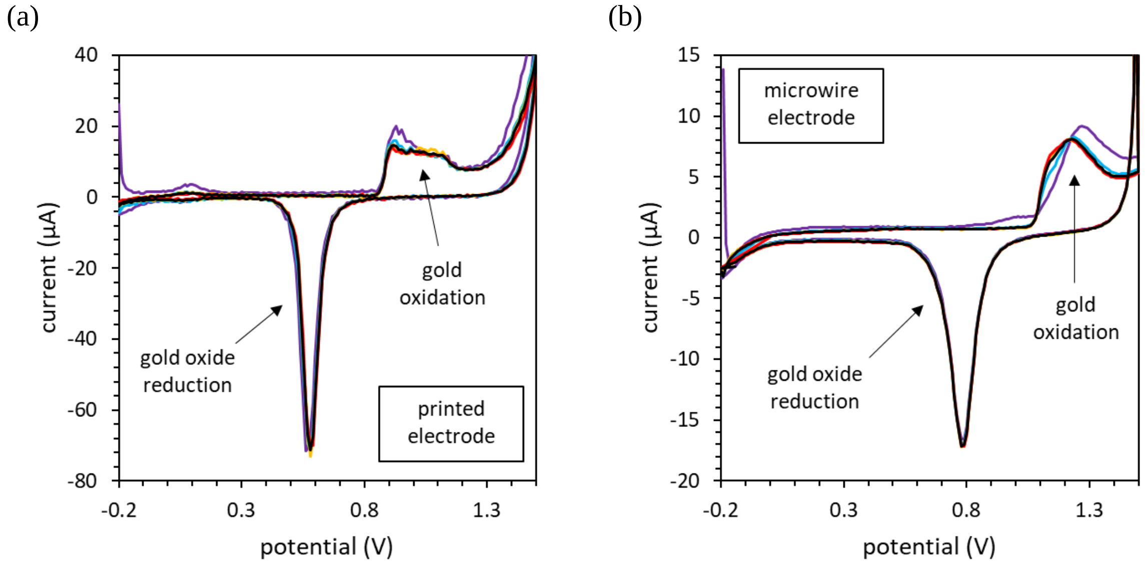

Figure S1: Example cyclic voltammograms. *Voltammograms are presented for the 1st (purple), 2nd (light blue), 3rd (light green), 4th (orange) and 5th (red) repeat scans, plus the average of scans two-to-five (black). Here, the 1st scan is not included in the average, as this scan often deviates from the other due to impurities in the voltammogram. CV scans were recorded in 0.5 M* H_2SO_4 *(40 mL) with deposition at -2.0 V (30 s) followed by 5 repeat CV scans between -0.2 and +1.5 V (10 mV per step, 0.36 V* s^{-1}*, 35.7±0.6 Hz sampling rate).*

The charges of the gold oxidation and reduction peaks in the anodic and cathodic scans respectively were determined from the area of each peak. For simplicity, the charge owing to formation of the gold oxide monolayer was evaluated by integration of the anodic peak from the left-hand side (i.e. ~0.8 and 1.0 V for the screen-printed and microwire electrodes respectively) to the local minimum (i.e. ~1.15

and 1.35 V in Figure S1b) [5]. The charge of the reduction peak of the screen-printed electrodes is typically marginally greater than that the oxidation peak (Figure S2).

The oxidation and reduction peaks are more intense using screen-printed electrodes. This is partially explained by the screen-printed working electrode having a larger surface area than the microwire electrodes (0.79 mm^2 versus 0.57 mm^2). However, the increasing surface roughness of the screen-printed electrodes with continued use likely explains more of the observed differences in CV peak intensities.

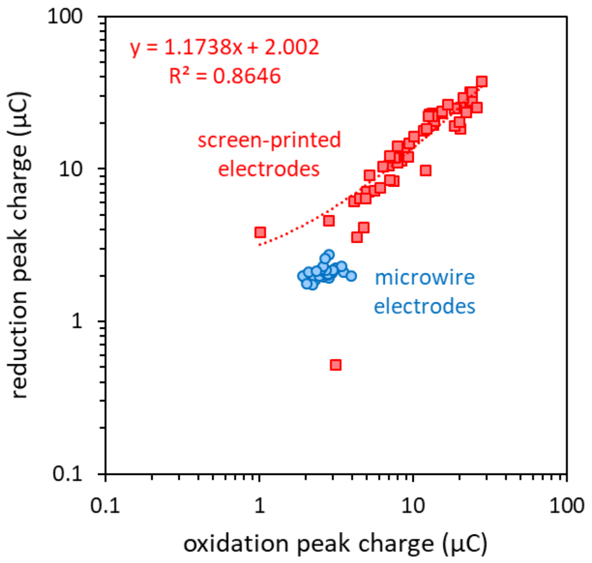

Figure S2: Correlation between the charge of the gold oxidation and reduction peaks in the cyclic voltammograms.

For each screen-printed electrode, the intensity of the gold oxidation and reduction peaks increases as the electrode is used (Figure S3a) and is explained by an increasing surface roughness of the gold working electrode, accompanied by visual observations of a colour change from gold to orange, presumably owing to nanoscale changes to the surface morphology. With excessive reducing current at the working electrode, the auxiliary electrode dissolves and the electrode malfunctions, highlighting that the stability of these electrodes is limited when applied in acidic solutions. Increases in the charge of the oxidation and reduction peaks appear to be also associated with repeated use of the same 0.5 M H_2SO_4 which was not replaced between each measurement, but rather used as a 'wash solution'. In contrast, the charge of the reduction peak obtained using microwire electrodes is relatively stable, indicating long electrode lifetimes and consistency between different electrodes (Figure S3b). In this case of microwire, a platinum (or iridium) auxiliary electrode was used, not a gold auxiliary electrode.

The minimum value of the reduction peak charge found for screen-printed electrodes when new is approximately 70% larger than the average charge of the microwire electrodes (new and used) (~3.5 µC and 2.1±0.2 µC respectively). This is larger than the 39% difference in geometric surface areas of the two electrodes (0.79 and 0.57 mm^2 respectively), indicating that the screen-printed electrodes have greater surface roughness. The smoother surface of the microwire electrode (with the roughness estimated at 1.2-1.3 in a previous study [5]) is likely to improve detection limits, since the mass transfer during the ASV deposition step is proportional to the geometric surface area whilst the charging current is proportional to the actual surface area, including surface roughness [1]. Increases in surface roughness thus decrease the signal-to-noise ratio.

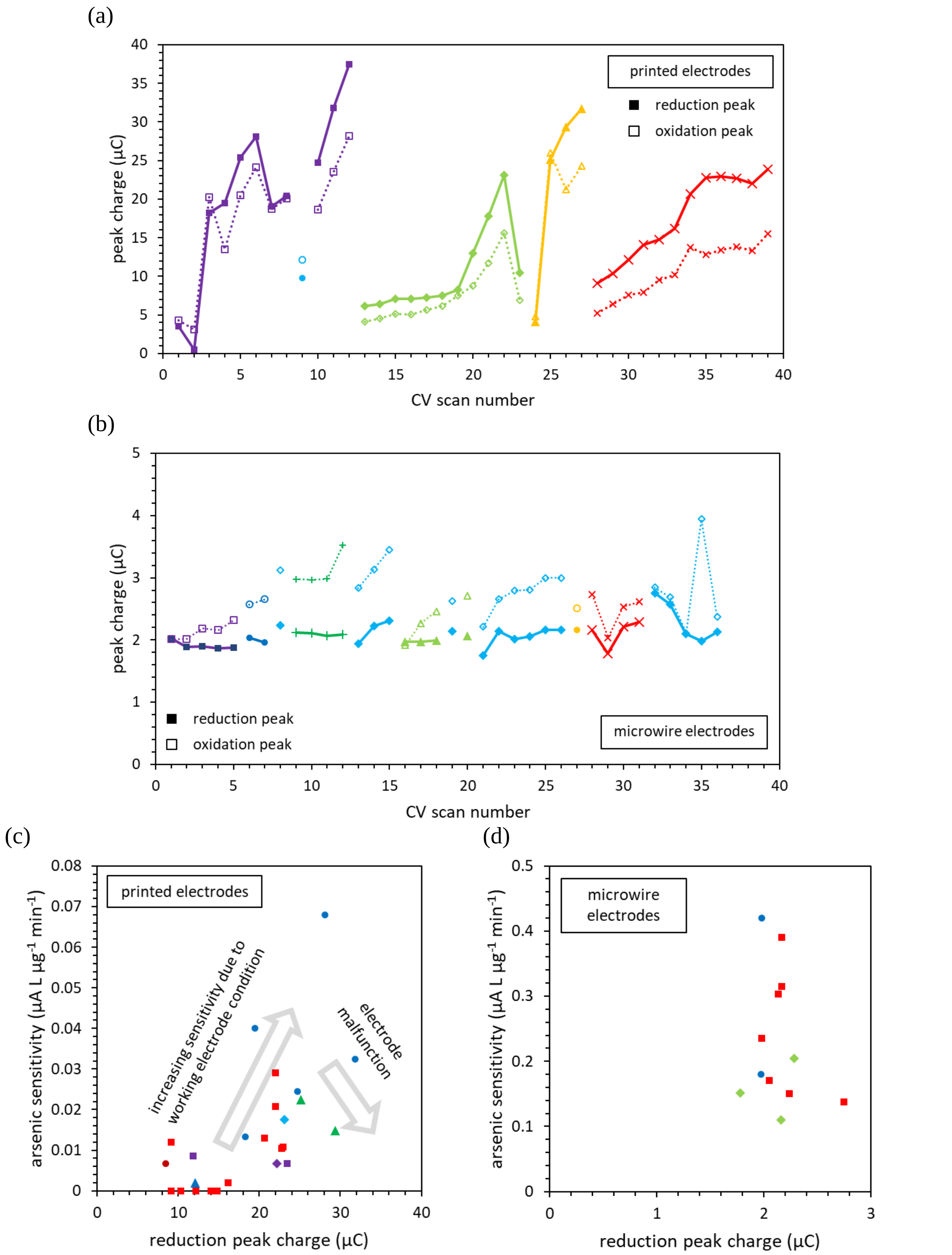

Figure S3: Comparison of electrode stability, as indicated by the magnitude of the reduction peak current, for (a) printed electrodes and (b) microwire electrodes. *Each data point represents the average peak height obtained from the 2nd, 3rd, 4th and 5th repeat scans. Solid shapes and lines indicate results obtained by calculating the charge of the reduction peak, whilst open shapes and dashed lines indicate the charge of the oxidation peak. The experimental conditions were 0.5 M H_2SO_4, 30 seconds cleaning at -2.0 V, 5 repeat CV scans between –0.2 and +1.5 V at a rate of 0.36 V s^{-1} (printed electrodes on the Rodeostat) and 1 V s^{-1} (microwire electrodes on the PalmSens 2). Panels (c) and (d) correlate the charge of the reduction peak with the sensitivity of the electrode towards the detection of total As at pH 1 (0.1 M HCl in deionised water). Sensitivity towards the detection of total As is normalised to the deposition time, since this varied between experiments (60 s or 240 s for the screen-printed electrodes in figure (c) and 20 s for the microwire electrodes in figure (d). There was no significant*

difference between the time-normalised sensitivity of the screen-printed electrodes with 60 s and 240 s deposition. Each symbol and colour combination represent a different electrode.

4. The importance of stirring and/or vibration

Earlier work has shown that vibrating microwire electrodes can achieve very low detection limits with short deposition times [6]. Whilst mechanical vibrators can be purchased at low-cost, mechanical or magnetic stirring remains the more common approach for mixing solutions during deposition. We consequently investigated how the choice of mixing options would influence the intensity and stability of the arsenic stripping peak (Figure S4).

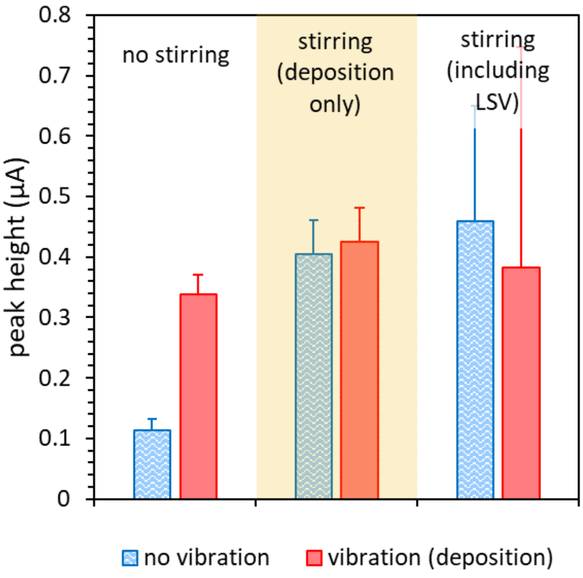

Figure S4: The influence of stirring and vibration on the detection of total As. *The detection of total As was achieved using a procedure of electrode cleaning (+0.7 V, 5 s), deposition (-1.3 V, 20 s), hold 1 (-0.4 V, 5 s), hold 2 (-0.4 V, 2 s), LSV from -0.4 to +0.7 V (1.2 V* s^{-1}*, 60 Hz sampling rate), cleaning (+0.7 V, 5 s), cell off and wait (10 s). A blank scan with a deposition time of 1 s was performed prior to each arsenic scan but was not needed for determining peak height. The bar chart presents the peak height average of 7 repeat scans, with error bars representing the standard deviation between the repeat measurements. The magnetic stirrer was either not used ('no stirring'), switched on manually for the deposition stage and switched off during the first hold potential ('deposition only'), or ran continuously for the entirety of each ASV procedure ('including LSV'). The vibrator was either not used ('no vibration'), or automatically switched on for the deposition step and switched off between the two hold potential steps ('deposition'). Mixing was continued during the first hold step to improve the desorption of matrix species and discontinued during the second hold step to allow the solution to settle. Experiments were performed using the PalmSens 2 potentiostat, which allowed for automated switching of the vibration. The sample was 5 µg* L^{-1} *total As in 0.1 M HCl.*

With neither stirring nor vibration, the stripping peak is small (0.11 µA with 5 µg L^{-1} As(V)). The intensity of the peak is improved both by vibrating or stirring during deposition (0.33 and 0.40 µA respectively). Combining stirring with vibration only has a limited improvement in the peak height (0.43 µA). Without stirring or vibration, the standard deviation in the peak height was 17%. This decreased to 10% with vibration during deposition, or 14% with stirring. This suggests that the arsenic deposition rate is more reproducible with vibration than when stirring, which is consistent with a previous study [7]. Combined stirring and vibration during deposition gave a standard deviation of 13%. When stirring was continued during the stripping step (LSV), the standard deviation of the peak height increased to 42 and 96% with and without vibration respectively. This is due to the superimposition of current waves over the voltammogram, which both constructively and destructively interfere with the arsenic peak.

Based on the As sensitivity, in this analytical procedure (using the Rodeostat with gold microwire electrodes under acidic conditions) the operator has a choice of stirring or vibration. However, generation of hydrogen and chlorine during deposition occasionally created significant interferences in the voltammogram (section 7). These interferences were best overcome through the combined application of both stirring and vibration. Due to the interference produced when mixing the solution during the analytical stripping step, mixing must be switched off prior to stripping. Whilst stirring was operated manually in this study, this creates potential human error. The automated switching of mixing should be automated to improve reproducibility in the analysis. Whilst the Rodeostat does not support the automated control of a stirring plate, a separate Arduino can be used for this purpose. Since the experimental work reported in this study, we have built an Arduino relay and updated our software to achieve automated stirring (see Supporting Information S2).

5. Quality of voltammograms and noise in the detection of total As

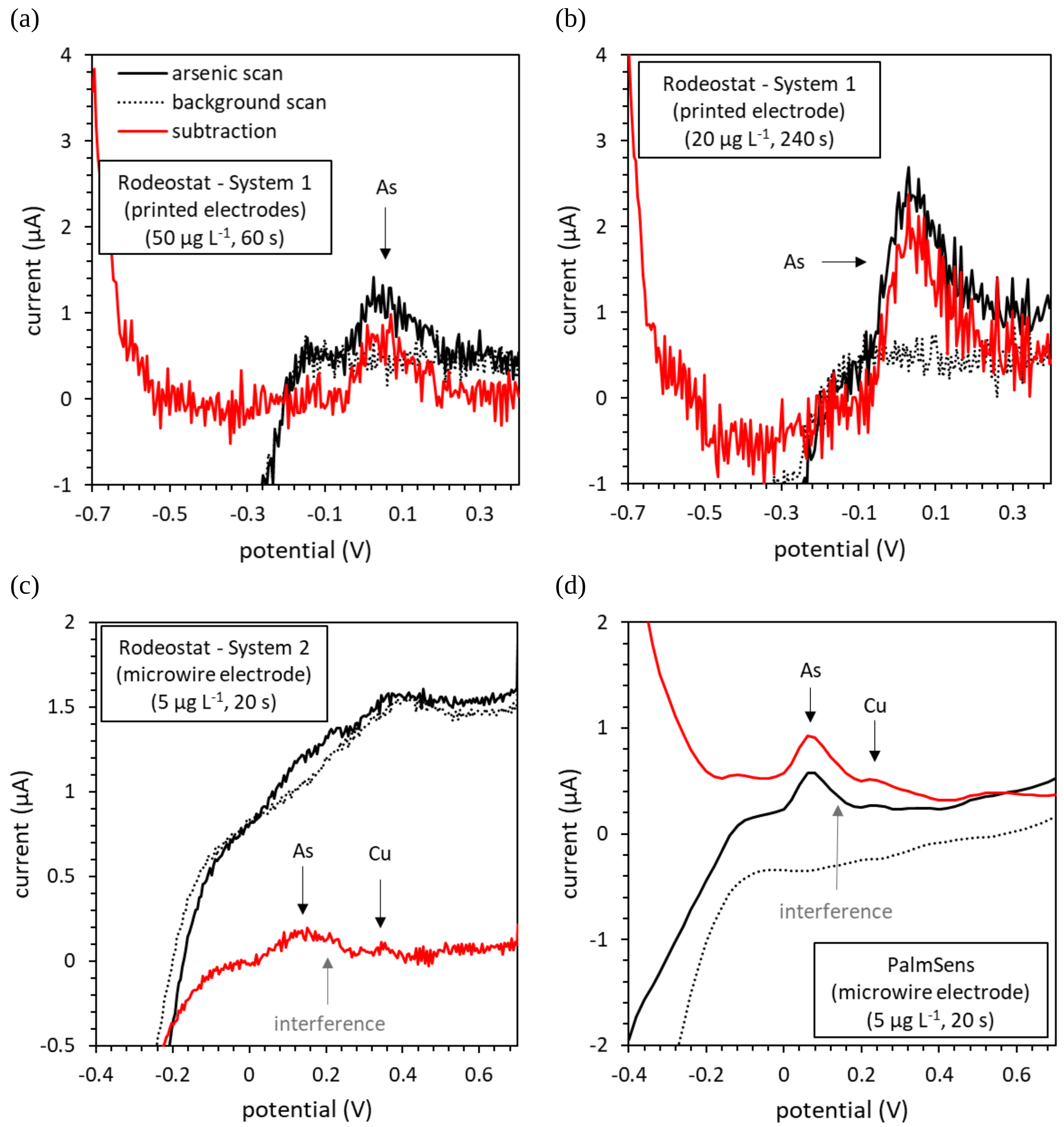

Figure S5: Example voltammograms showing total As detection using anodic stripping voltammetry (ASV) under acidic conditions (pH 1, 0.1 M HCl). *Voltammograms were recorded using the Rodeostat with screen-printed electrodes and a deposition time of (a) 60 s and (b) 240 s, and microwire electrodes with a deposition time of 20 s using (c) the Rodeostat, and (d) the PalmSens 2. The analytical scan for the detection of arsenic has the full deposition time (black solid lines) whilst the background scan has a deposition time of one second only (grey dotted lines). The results of the background subtraction are presented as red solid lines.*

The noise was greater in the printed electrode system than the microwire systems. However, the decrease in noise after changing to the microwire electrode system was not only due to the different configuration of electrodes, but also due to the replacement of an unshielded cable connecting the electrodes to the potentiostat with a shielded cable (an ethernet cable, Figure S6). The effect of the two improvements seemed to be roughly equivalent: using a shielded cable reduced the noise by 64%, whilst replacing the screen-printed electrodes with the microwire electrode arrangement reduced the noise by 66%. The geometric surface area of the screen-printed and microwire electrodes are similar (0.79 mm^2 and 0.57 mm^2 respectively). Assuming a linear relationship between geometric area and noise, differences in the geometric surface area of the two electrode designs account for 28% of the

noise reduction when switching from screen-printed to microwire electrodes. The remaining reduction is noise may be due to the more favourable signal-to-noise ratio, *i.e.* the lower surface roughness of the microwire electrode and the cylindrical diffusion that improves mass transfer (*i.e.* higher signal).

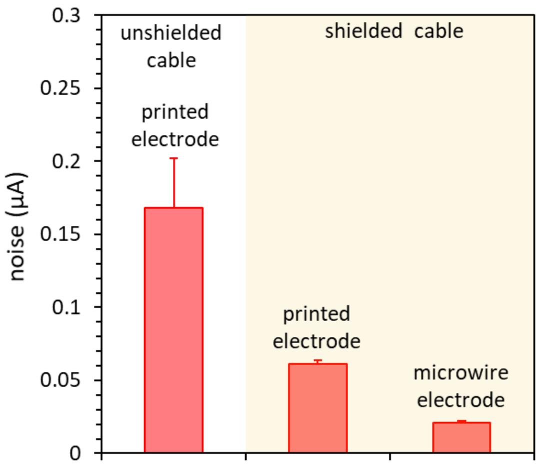

Figure S6: Quantitative comparison of voltammogram noise in three different arrangements of the Rodeostat system. *The noise was calculated by choosing a relatively flat region of the background scan (1 s deposition time) of the blank electrolyte (0.1 M HCl), fitting a linear regression to this region (+0.05 to +0.2 V was selected, with ~40 data points), and calculating the sum of squared residuals between the experimental data and the linear regression. The sum of squared residuals was divided by the number of data points, and the square root was then taken, to provide a measure of the noise typically found for each data point, with the units μA. The noise on the PalmSens system was not calculated for comparison, given that the background scans with 1 s deposition time were not flat in the +0.05 to +0.2 V region, and the large step potential (20 mV) means that there are only seven data points in this region, preventing a statistical analysis. However, once synchronised to 60 Hz (the frequency of mains electricity in Mexico), no noise was detected in the PalmSens system.*

The shoulder to negative currents on the left-hand side of the voltammogram begins at *ca.* -0.2 V when using the printed electrodes, and -0.1 V using the microwire electrodes, in line with the observed difference between the reference potentials (see section 3). The screen-printed electrodes have more negative current at the start of the linear sweep (-20 to -30 μA at -0.7 V) than the microwire electrodes (-10 μA at -0.4 V using the Rodeostat), however at -0.4 V, this negative current has mostly disappeared when using the screen-printed electrodes (-2 μA only). Using the Rodeostat, the arsenic scan (with deposition times >1 s) mostly overlaps with the background scan (with 1 s deposition), irrespective of the type of electrode used or the deposition times. In contrast, the baseline current of the background scan obtained using the PalmSens system is routinely *ca.* 1 μA below the arsenic scan.

6. PalmSens 2-microwire electrode system

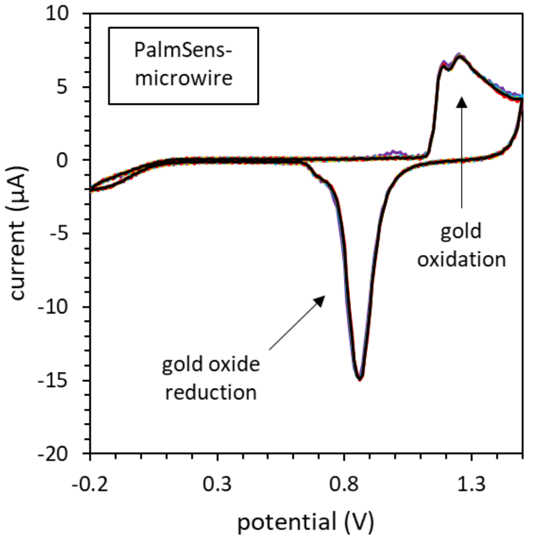

Figure S7: Typical cyclic voltammogram for the gold microwire electrode in 0.5 M H_2SO_4 using the PalmSens 2 potentiostat. *The operating parameters were electrode conditioning at -2.0 V (30 s) followed by 5 repeat CV scans between -0.2 and +1.5 V (10 mV step, 1 V s^{-1}, 100 Hz sampling rate).*

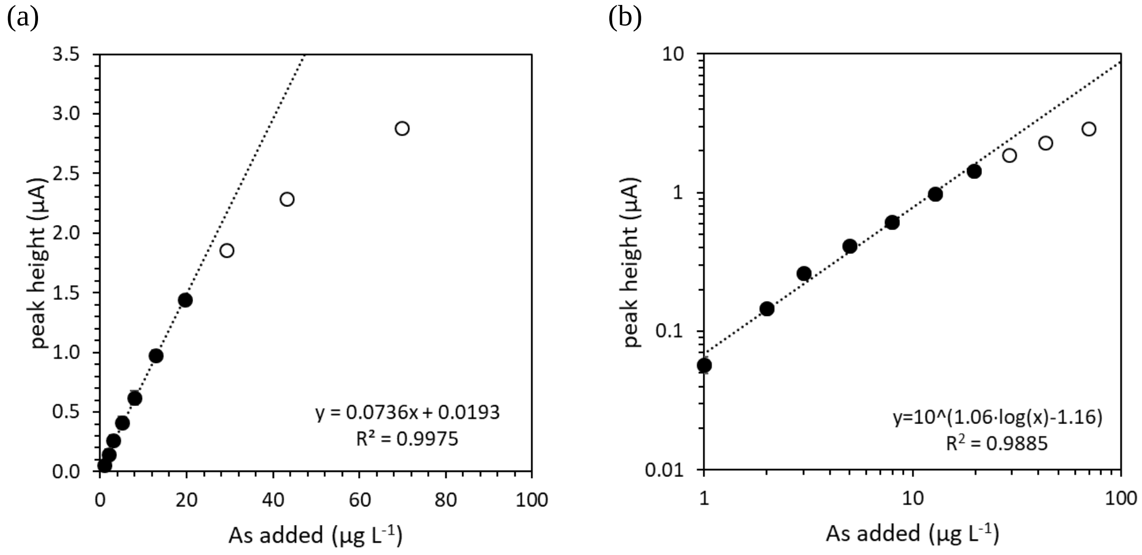

Figure S8: Linear range for total As detection using gold microwire electrodes and the PalmSens 2 potentiostat in (a) linear and (b) log-scale. *The experimental conditions were pH 1 (0.1 M HCl), cleaning at +0.7 V (5 s), deposition with vibration at -1.3 V (20 s in the analytical scan, 1 s only in the background scan), hold at -0.4 V (5 s with vibration plus 2 s without vibration), LSV stripping from -0.4 to +0.7 V at a rate of 1.2 V s^{-1}, sampling frequency of 60 Hz and a 20 mV step size), cleaning at +0.7 V (5 s), and cell off for 5 s to rest.*

7. Interference during the detection of total As using microwire electrodes with long deposition times and repeat scans

Using the microwire electrode configuration, an interference at *ca.* -0.2 V appeared with long deposition times (Figure S9). Increases of this interference were correlated with a loss of the As stripping peak. This interference was also associated with the development of a chlorine smell. The interference was especially prevalent when using a small sample cell volume (10 mL) with limited headspace for ventilation. Whilst unidentified, this interference only appears when producing significant amounts of hydrogen (*i.e.* deposition at low pH, low deposition potentials, and long deposition times). This interference was best overcome by using larger cell volumes with more airspace and by improving mixing during deposition through combining stirring with vibration.

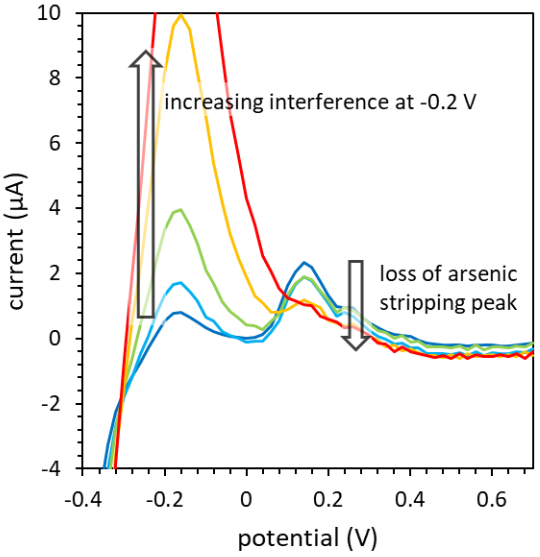

Figure S9: An interference at -0.2 V is associated with long As deposition times. *Total As detection was carried out with a deposition potential of -1.2/-1.3 V (60 s). The sample solution was 5 µg L^{-1} As(V) in 0.1 M HCl.*

8. Arsenic peak stability

With good mixing and deposition times no longer than 20 s, the total As peak using microwire electrodes was stable for at least 30 scans (Figure S10).

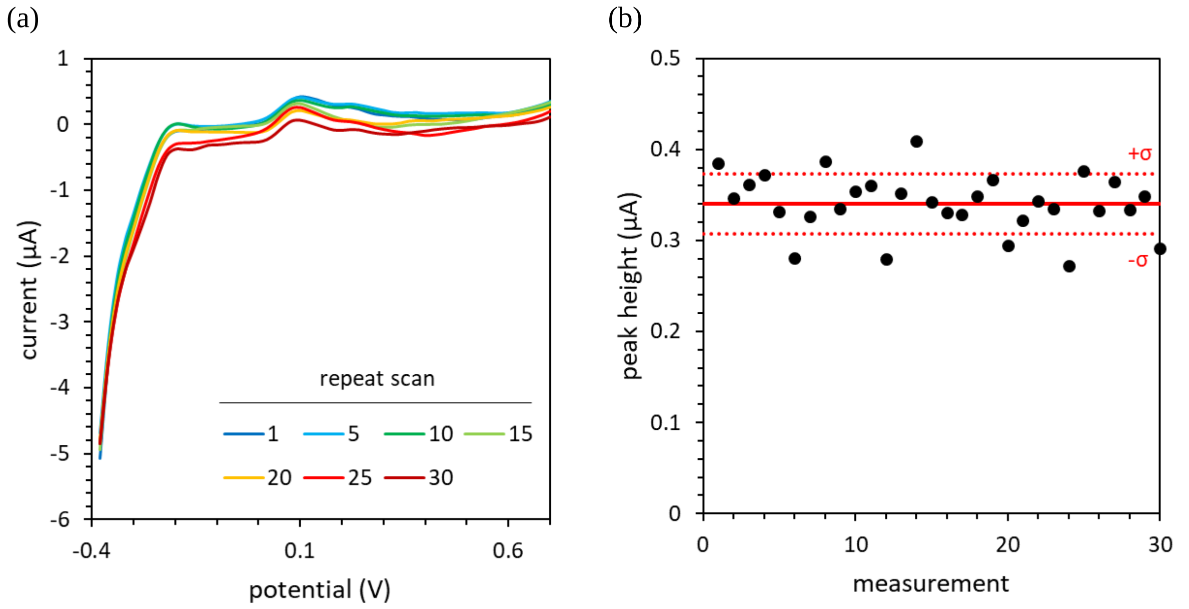

Figure S10: Stability of the arsenic peak during total As detection using a gold microwire electrode at pH 1, with 30 repeat scans. *The sample was 5 µg L^{-1} As(V) in 0.1 M HCl and the deposition time was 20 s. The PalmSens 2 potentiostat was used here, instead of the Rodeostat, since switching of the vibration could be automated, removing human error. The solution was vibrated only during deposition, and not stirred.*

9. Equal sensitivity to As(III) and As(V)

As discussed in the main text, under acidic conditions and with low deposition potentials As(III) is rapidly oxidised to As(V) at the counter electrode during the first few seconds of the deposition step. All arsenic is consequently deposited onto the working electrode from the +5 oxidation state, irrespective of the original speciation. The equal sensitivity of the method towards the detection of inorganic As(III) and As(V) that results from this chemistry was verified using spike recovery experiments. A sample solution was prepared using As(III) or As(V) in 0.1 M HCl and then analysed using the method of standard additions, using a standard solution of arsenic in the other oxidation state (i.e. As(III) samples were analysed using an As(V) standard and vice versa). A 100% spike recovery was achieved using both As(III) samples/As(V) standards and As(V) samples/As(III) standards and the microwire electrodes, indicating that this method is equally sensitive towards both arsenic species (Figure S11).

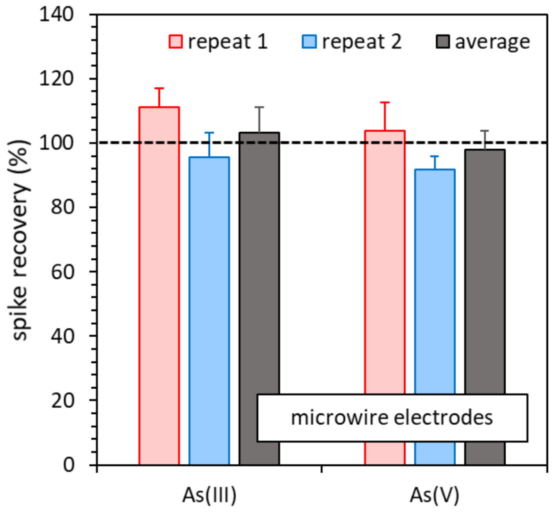

Figure S11: Spike recovery experiments used to evaluate the sensitivity of the total As detection techniques towards As(III) and As(V) using microwire electrodes. *The sample solutions were 5 µg L^{-1} As(III) or As(V), in 0.1 M HCl. The spike recovery was achieved using two standard additions of 2.5 µg L^{-1} As(V) or As(III). Error bars indicate the uncertainty in each result, determined from the error in the linear regression fit to the standard addition calibration curve. Two repeat experiments were performed using the microwire electrodes, and one experiment for the printed electrodes.*

10. Measuring arsenic using the method of standard additions

An example of the standard addition procedure used to determine the concentration of total As in this work is presented in Figure S12. The Rodeostat and microwire electrode configuration was used in this example. Where the concentration of total As in the sample is believed to be >50% of the linear range, the sample is diluted with deionised water. The sample is acidified to pH 1 using 1 M HCl. The sample solution is scanned ideally at least three times to achieve consistent As peaks. A small volume of an arsenic stock solution is added with the aim of increasing the height of the As peak by ~50%. This is the first standard addition, and calibration curves from previous experiments can be used to estimate the quantity of standard needed. After the first standard addition has been analysed, a second addition and potentially a third addition are made. The height of the As peak after the final addition should be approximately twice as large as the height of the As peak in the sample prior to addition of the standard solution (Figure S12a). A linear regression is fitted to the graph of As peak height versus the concentration of standard in the cell (Figure S12b) and this is extrapolated to the x-axis intercept to determine the concentration of arsenic present before addition of the standard. Uncertainties are calculated as explained in the main text. Arsenic concentrations are adjusted to account for any dilution of the sample.

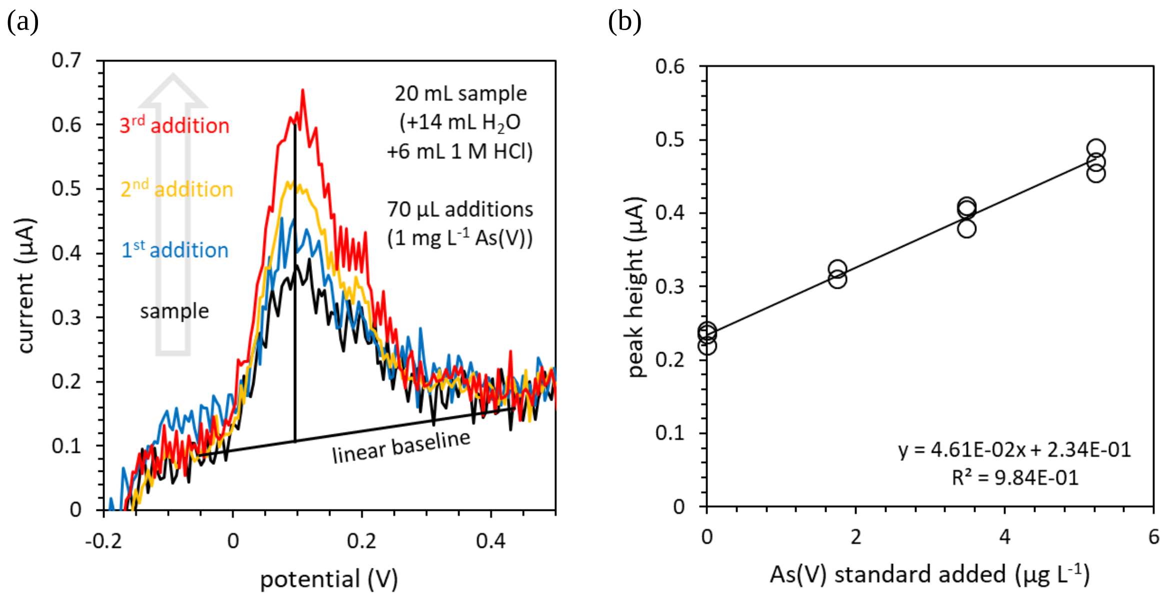

Figure S12: Example of the standard addition procedure used to determine total As in this work. *Whilst three repeat scans were made after each addition of the standard, for clarity only the first scan is shown. A linear baseline is fitted to each voltammogram (though only a single baseline is shown in figure (a) for clarity). In this case, the baseline has been extended to +0.45 V as to bypass the small copper peak at +0.35 V. The concentration of total As in this sample was 10.2±0.4 µg L^{-1} as determined by ASV and 10.0 µg L^{-1} as determined by AAS.*

11. Characterisation of natural water samples

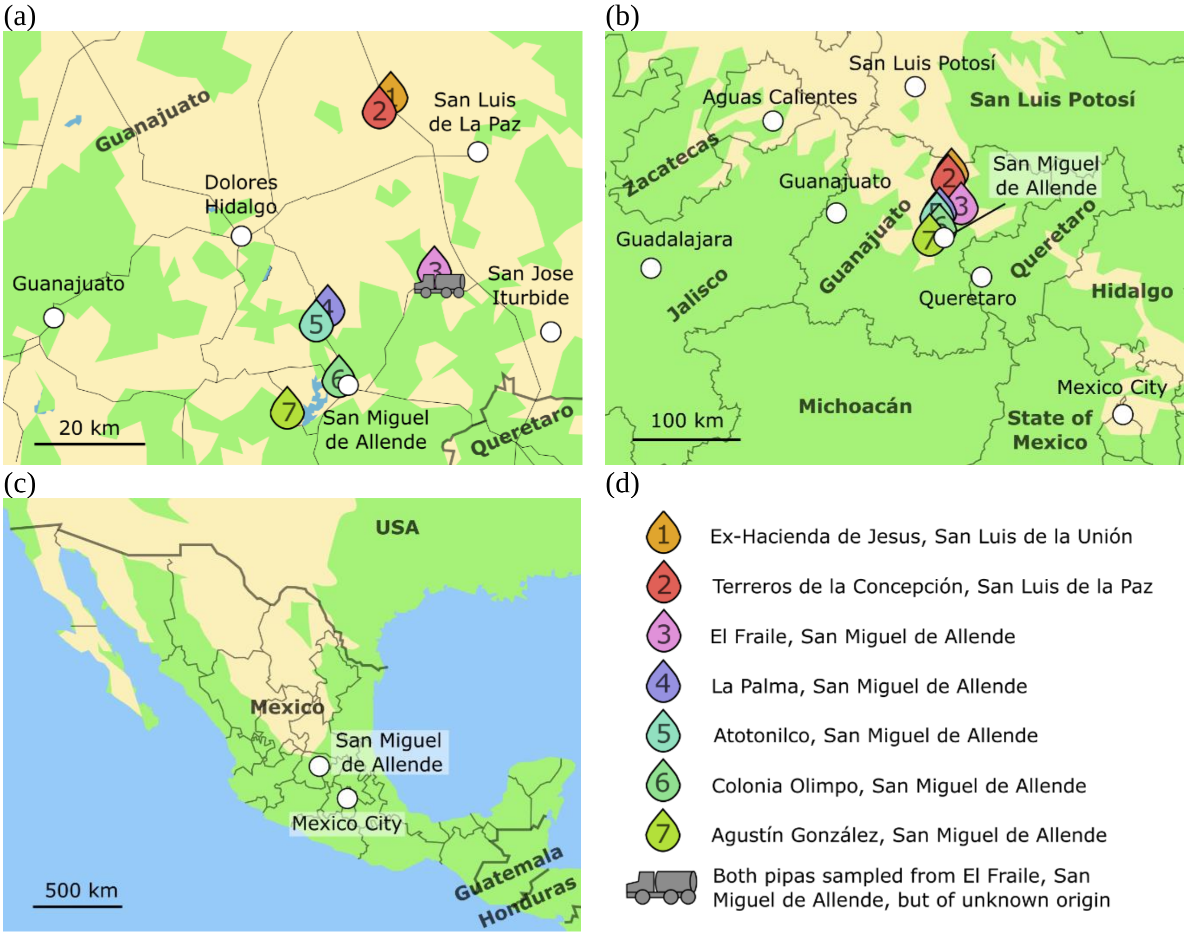

Figure S13: Map of sampling locations. *(a,b,c) Sampling locations in the state of Guanajuato at different distance scales and (d) the accompanying legend. Different sampling locations are numbered in order from northern-most to southern-most, whilst the truck icon represents the pipas (water delivery trucks) that delivered water of unknown origin to El Fraile, San Miguel de Allende. A data table characterising all water samples is presented in the main text.*

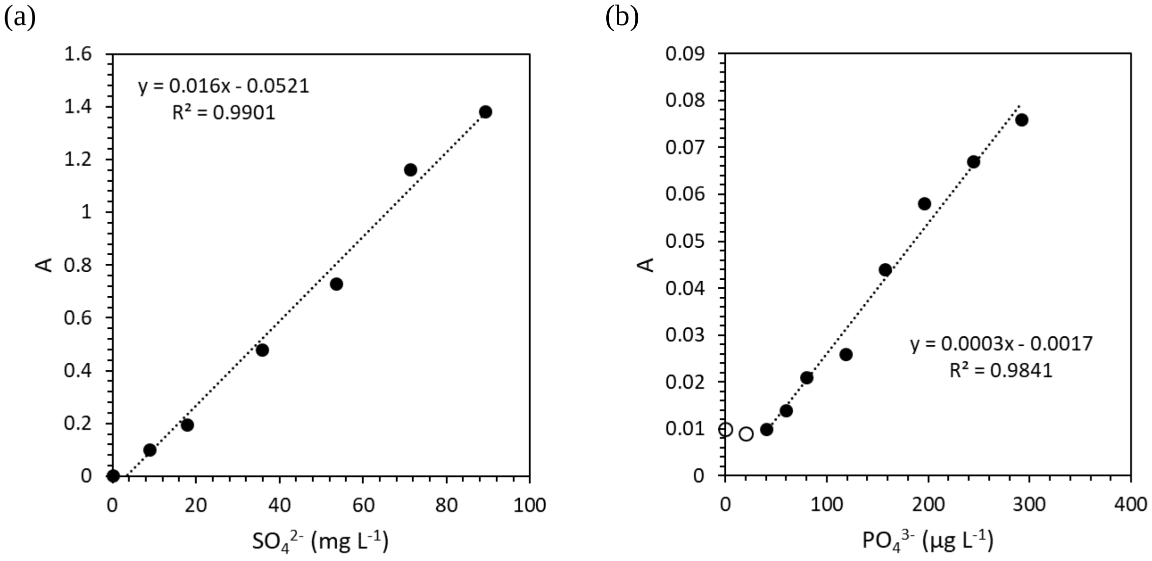

Figure S14: Calibration curves for (a) sulphate and (b) phosphate using the Hach DR900. *The y-axis indicates the measured absorbance (A). Detection limits were calculated from the calibration curve using the formula* $LoD = a + 3 \cdot s_y$ *where a is the y-intercept of the linear regression fit to the calibration curve, and* s_y *is the standard error in the y-intercept (both obtained using the LINEST function in Microsoft Excel). Detection limits were 6.7 mg* L^{-1} *for sulphate and 27 µg* L^{-1} *for phosphate. Fluoride measurements were recorded using the calibration curve already set on the Hach DR900.*

A recent study of approximately 100 wells in the Upper Río Laja Watershed sampled in the present study, found an average F^- concentration of 1.3 mg L^{-1} and a maximum of 15.5 mg L^{-1}. High fluoride concentrations have been identified elsewhere in Mexico, e.g. 3.6 mg L^{-1} in Zacatecas [8], 6 mg L^{-1} in Durango [9] and 17 mg L^{-1} Michoacán [10].

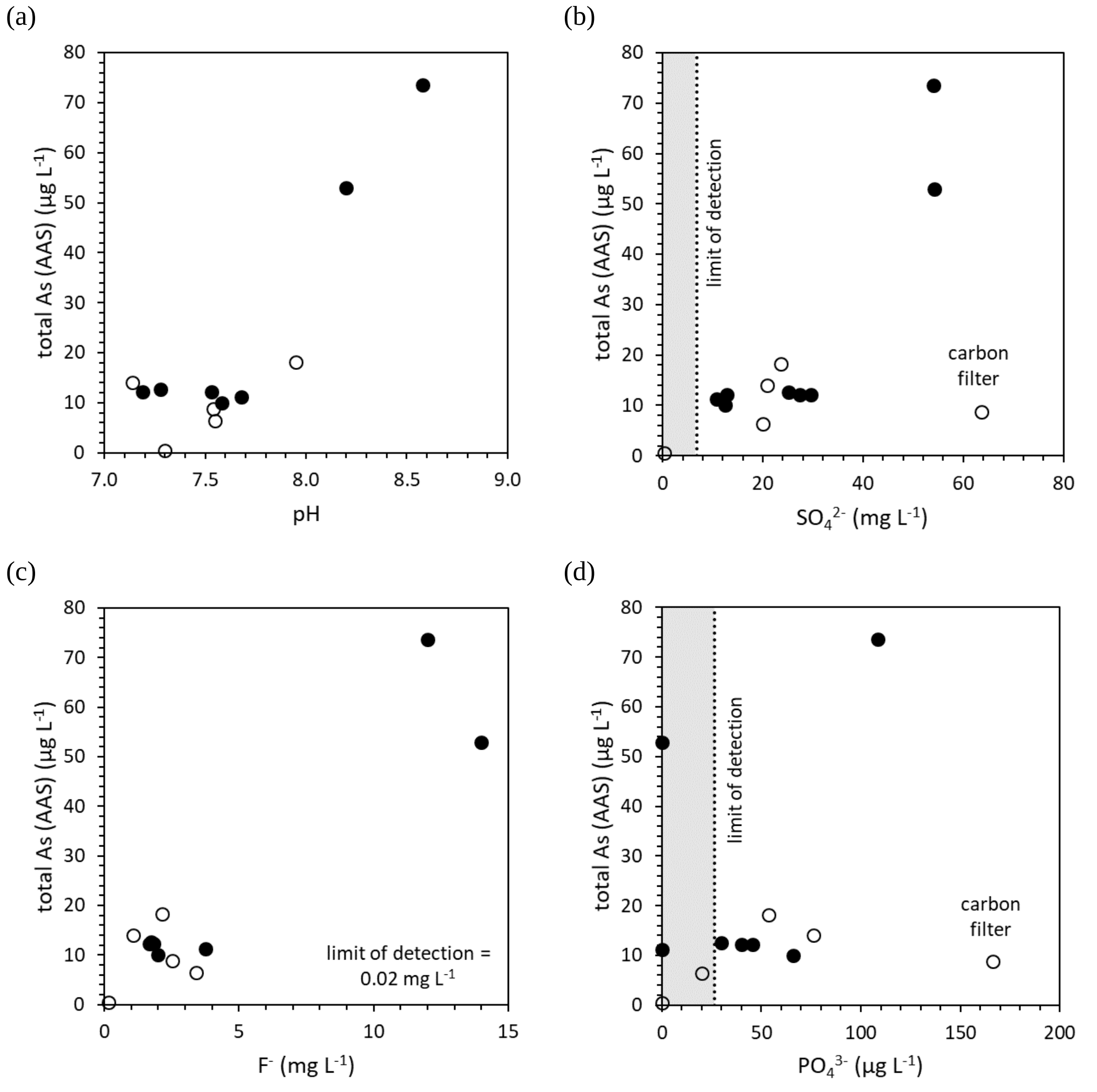

Figure S15: Characterisation of real water samples. *Untreated groundwater samples are represented by filled black circles, and all other samples by open/white circles. The detection limit (LoD) of the AAS method used to determine total As is approximately 1 µg* L^{-1}*.*

12. Electrode sensitivity in natural matrices

The sensitivity of the electrochemical techniques in natural samples were determined from the slope of the linear regression fit to the standard addition calibration curve, and a comparison of the sensitivity of each technique is given in Figure S16. The sensitivity of the screen-printed electrodes towards a given concentration of arsenic increases by a factor of four when the deposition time is increased from 60 seconds to 240 seconds, as expected (from 6.7 nA (μg $L^{-1})^{-1}$ to 29 ± 11 nA (μg $L^{-1})^{-1}$). The sensitivities of the different electrodes on the Rodeostat cannot be directly compared, given that analysis was conducted using a sweep rate of 0.22 V s^{-1} and 45.2±0.9 Hz for the screen-printed electrodes (with 240 s deposition) and 1.2 V s^{-1} and 240 Hz for the microwire electrodes (with 20 s deposition). However, the sensitivity increased from 29 ± 11 nA (μg $L^{-1})^{-1}$ to 44 ± 8 nA (μg $L^{-1})^{-1}$ when changing from System 1 with the screen-printed electrodes to System 2 with the microwire electrodes. Normalising to account for differences in deposition time and voltage sweep rates suggests that the microwire electrodes are ~3.4 times more sensitive than the printed electrodes. The greater sensitivity of the microwire electrodes versus the screen-printed electrodes is despite the microwire electrode having a smaller geometric surface area than the screen-printed electrodes (0.79 mm^2 and 0.57 mm^2 respectively). Much of the increased sensitivity is due to the cylindrical diffusion that enhances mass transport at the microwire in comparison to the linear diffusion that takes place at the screen-printed electrode.

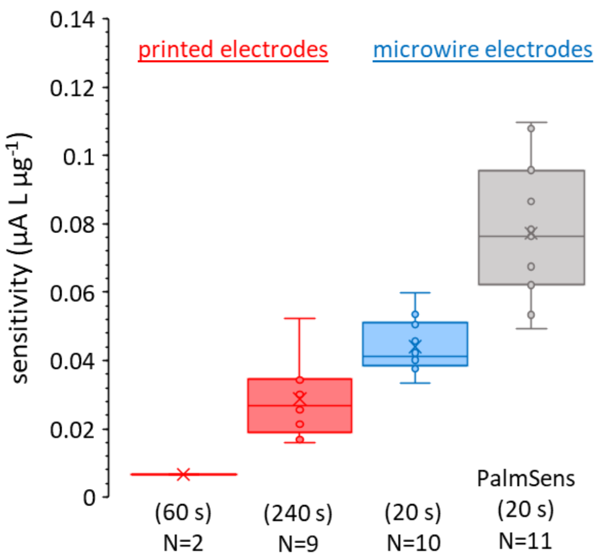

Figure S16: Electrode sensitivity, calculated from the slope of the linear regression in the standard addition calibration curve obtained during the analysis of the natural samples in this study. *The box and whisker plots show the Rodeostat equipped with screen-printed electrodes and a deposition time of 60 seconds (red, far left) and 240 seconds (red, centre left), the Rodeostat equipped with microwire electrodes and a deposition time of 20 seconds (blue, centre right), and the PalmSens equipped with microwire electrodes and a deposition time of 20 seconds (grey, far right). The boxes indicate the 25, 50 and 75% percentiles, whilst the cross indicates the average value. The whiskers represent minima and maxima values, excluding outlier data points which are defined as values either greater than the top of the box plus 1.5 times the interquartile range, or the bottom of the box subtract 1.5 times the interquartile range. Cross symbols indicate the mean value. Results are presented using the Rodeostat and screen-printed electrodes (the two red boxes, left), the Rodeostat and microwire electrodes (blue box, centre right) and the PalmSens and microwire electrodes (grey box, far right). The stripping conditions were 0.19 V* s^{-1} *and 45.2±0.9 Hz for the screen-printed electrodes, 1.2 V* s^{-1} *and 240 Hz for the Rodeostat equipped with microwire electrodes, and 1.2 V* s^{-1} *and 60 Hz for the PalmSens. The kitchen tap water sample was not included in this figure, as the arsenic content in this sample was below the detection limit in most analyses. The groundwater sample that had been passed through a sediment filter and then a carbon block filter was also not included, since this was a severe outlier, as discussed above.*

The average sensitivity increased by a factor of 1.8 when changing from the Rodeostat-microwire system to the PalmSens-microwire system (from to 44 ± 8 nA (µg L^{-1})$^{-1}$ to 77 ± 21 nA (µg L^{-1})$^{-1}$). Both systems used a deposition time of 20 s and a scan rate of 1.2 V s^{-1}. However, the potential step and sampling rates were 5 mV and 240 Hz respectively in the Rodeostat system, and 20 mV and 60 Hz in the PalmSens system. There was greater variation in the sensitivity of the measurements made using microwire electrodes when using the PalmSens, compared to the Rodeostat, however this is most likely explained by microwire electrodes being used at a later life stage on the PalmSens system, where the condition of the electrode surface was less stable (*e.g.* the potential adsorption of organic matter).

One sample was a significant outlier: The groundwater sample that had been passed through a sediment filter and then a carbon block filter significantly supressed the sensitivity of the microwire electrode. Using the Rodeostat, the sensitivity decreased from an average of 44 ± 8 nA (µg L^{-1})$^{-1}$ to just 9.8 nA (µg L^{-1})$^{-1}$ and using the PalmSens, the sensitivity decreased from an average of 77 ± 21 nA (µg L^{-1})$^{-1}$ to just 11 nA (µg L^{-1})$^{-1}$. In contrast, the sensitivity of the screen-printed electrodes was unchanged in this sample matrix (27 nA (µg L^{-1})$^{-1}$ is within the average sensitivity of 29 ± 11 nA (µg L^{-1})$^{-1}$ using a 240 s deposition time).

This sample is markedly different from all others. The pH is typical of this sample set (pH 7.54 compared with a median of 7.55 ± 0.45 for all other samples) and the arsenic concentration was low (8.8 µg L^{-1}). However, this sample has high total dissolved solids (TDS), at 422 mg L^{-1} compared with a maximum of just 228 mg L^{-1} for all other samples. This sample has the highest PO_4^{3-} concentration (166 µg L^{-1} versus a median value of 35 µg L^{-1} and a maximum of 108 µg L^{-1} for all other samples. Here, high PO_4^{3-} concentrations are normally associated with high As concentrations, not mirrored in this sample). The sample also has the highest SO_4^{2-} concentration (64 mg L^{-1} versus a median of 22 mg L^{-1} and a maximum of 54 mg L^{-1} for all other samples. Again, in the other samples, high SO_4^{2-} concentrations are associated with high As concentrations). This sample is also high in F^- (2.5 mg L^{-1} versus a median of 1.9 mg L^{-1} for all other samples).

References

(1) Salaün, P.; van den Berg, C. M. G. Voltammetric Detection of Mercury and Copper in Seawater Using a Gold Microwire Electrode. *Anal. Chem.* **2006**, *78* (14), 5052–5060. https://doi.org/10.1021/ac060231+.

(2) Gun, J.; Salaün, P.; van den Berg, C. M. G. Advantages of Using a Mercury Coated, Micro-Wire, Electrode in Adsorptive Cathodic Stripping Voltammetry. *Anal. Chim. Acta* **2006**, *571* (1), 86–92. https://doi.org/10.1016/j.aca.2006.04.043.

(3) Salaün, P.; Planer-Friedrich, B.; van den Berg, C. M. G. Inorganic Arsenic Speciation in Water and Seawater by Anodic Stripping Voltammetry with a Gold Microelectrode. *Anal. Chim. Acta* **2007**, *585* (2), 312–322. https://doi.org/10.1016/j.aca.2006.12.048.

(4) Gibbon-Walsh, K.; Salaün, P.; van den Berg, C. M. G. Determination of Arsenate in Natural PH Seawater Using a Manganese-Coated Gold Microwire Electrode. *Anal. Chim. Acta* **2012**, *710*, 50–57. https://doi.org/10.1016/j.aca.2011.10.041.

(5) Oesch, U.; Janata, J. Electrochemical Study of Gold Electrodes with Anodic Oxide Films-I. Formation and Reduction Behaviour of Anodic Oxides on Gold. *Electrochim. Acta* **1983**, *28* (9), 1237–1246. https://doi.org/10.1016/0013-4686(83)85011-7.

(6) Gibbon-Walsh, K.; Salaün, P.; van den Berg, C. M. G. Arsenic Speciation in Natural Waters by Cathodic Stripping Voltammetry. *Anal. Chim. Acta* **2010**, *662* (1), 1–8. https://doi.org/10.1016/j.aca.2009.12.038.

(7) Salaün, P.; Gibbon-Walsh, K. B.; Alves, G. M. S.; Soares, H. M. V. M.; van den Berg, C. M. G. Determination of Arsenic and Antimony in Seawater by Voltammetric and Chronopotentiometric Stripping Using a Vibrated Gold Microwire Electrode. *Anal. Chim. Acta* **2012**, *746*, 53–62. https://doi.org/10.1016/j.aca.2012.08.013.

(8) Navarro, O.; González, J.; Júnez-Ferreira, H. E.; Bautista, C. F.; Cardona, A. Correlation of Arsenic and Fluoride in the Groundwater for Human Consumption in a Semiarid Region of Mexico. *Procedia Eng.* **2017**, *186*, 333–340. https://doi.org/10.1016/j.proeng.2017.03.259.

(9) Martínez-Cruz, D. A.; Alarcón-Herrera, M. T.; Reynoso-Cuevas, L.; Torres-Castanõ´n, L. A. Space-Time Variation of Arsenic and Fluoride in Groundwater in the City of Durango, Mexico. *Tecnol. y Ciencias del Agua* **2020**, *11* (2), 309–340. https://doi.org/10.24850/j-tyca-2020-02-09.

(10) Armienta, M. A.; Segovia, N. Arsenic and Fluoride in the Groundwater of Mexico. *Environ. Geochem. Health* **2008**, *30* (4), 345–353. https://doi.org/10.1007/s10653-008-9167-8.
