# Supplementary material for: Low-cost electrochemical detection of arsenic in the groundwater of Guanajuato state, central Mexico using an open-source potentiostat
Source: PLoS One. 2022 Jan 19;17(1):e0262124. doi: 10.1371/journal.pone.0262124 (PMC8769315; doi:10.1371/journal.pone.0262124)
Supplement: S2 File — (ZIP) [file pone.0262124.s002.zip › Software version 1 (as used in paper)/Setting up the software (early guide).docx]

28-August-2019

This document is being prepared as the “master” software documentation for the Rodeostat potentiostat. It will absorb and replace the existing “Rodeostat Home Software Notes” document.

Contents:

I. Document and Software revision log

II Introduction and overview

III Installing software on a Windows PC

IV Command set and structure of data files

V Command Line operation from Python.

VI GUI operation

VII Source codes (Python and GUI)

# I. Document Revision Log

Document Version 30-Nov-2019. Updated README file contains new program download and installation instructions.

Document Version 15-Sept-2019. Software Versions 15-Sept-2019. This a first draft to the document. Also, software updates are in progress and will be listed here. All comments and suggestions are welcome ([larry@caminosdeagua.org](mailto:larry@caminosdeagua.org) or Slack, or wherever).

# II Introduction and Overview

Introduction

The Rodeostat potentiostat is entirely software driven by a computer through its USB port. There are no knobs, switches, etc., of any sort.

At the present time, software for the Rodeostat is available in two forms:

1. Rodeostat offers interactive online software. This is fully documented at <https://blog.iorodeo.com/rodeostat-software/> and will not be discussed further in this document.

The Rodeostat online software has a graphical interface, many choices of driving waveforms with user-settable parameters. It provides good graphical and data file outputs.

The disadvantage of the Rodeostat software is that the user cannot “stack” several waveform and parameters into a savable user “program.” Also, it is not extendable – the available waveforms are all you can get.

2. Rodeostat offers a PC driver “hook”, written in Python, for commanding the Rodeostat (parameters and voltage) vs time and collecting sensed data (current vs time). This package makes it possible to put together whatever is desired terms of an entire test and data collection program, with the user interface defined by the programmer.

The design, installation and operation of this latter software is the subject of this document.

Overview

Rodeostat provides a Python module for addressing data to and retrieving data from the Rodeostat potentiostat. (<http://stuff.iorodeo.com/docs/potentiostat/> )

Rodeostat also provides several demonstration examples of the use of this “hook” to run the Rodeostat. The documentation and demonstration examples make writing user software in Python straightforward, and that is what was done.

The Python program “ArsenicTest.py” runs as a standalone program. Since Python is an interpreted language, this program must be run within Python – this is detailed in section V. This program reads data from a data file, sends the correct information (voltage vs time) to the potentiostat, collects the returning current vs time information, graphs the voltage and current vs time, and offers the user the option to save the output data file. Each line in the data file (after 2 configuration lines) describes a predefined voltage waveform and its parameters. A data file can therefore describe an arbitrarily involved process consisting of many different voltage waveforms.

The program looks for a Rodeostat connected to USB port 3 when it begins. If it does not find a Rodeostat, it automatically defaults (after presenting an information message) to a “simulation” mode in which an internal subroutine “pretends” it’s a 100,000 ohm resistor connected to the Rodeostat. This lets the user run a data file without actually setting up the Rodeostat and electrodes and chemistry. Since the program is simulating the Rodeostat, however, this runs in real time – a 15 minute long process, for example, takes 15 minutes to run.

This operation, as described, allows the user to do whatever they want to do. Also, the Python program may be upgraded at any time to include to waveform descriptions. Operating the program, however, is somewhat tedious – the data file must be prepared and modified in a text editor and can only be checked to see if it’s correct in real time.

A GUI interface to the Python program was written using the XOJO programming language. This language is very similar to Visual Basic, but is much “friendlier” than Visual Basic in terms of moving a program between machines and, optionally, compiling a program into a stand-alone executable version.

The executable GUI program “Arsenic App.exe” allows the user to create and edit a data file, showing the full data file in a dedicated window. It takes care of the (comma separated variable) formatting of the data file. It also has a graphics window which displays the process run described by the data file instantaneously (as opposed to real time). The graphics window can zoom in on pieces of a complicated waveform for examination. The GUI program has a command to run the Python program, and will pass the data file to the Python program directly.

# III Installing software on a Windows PC

The software installation is a simple, albeit annoying process. Fortunately, except of course for upgrades, this all only has to be done once. The system requirement is a 64 bit Windows PC, running (at the time of writing) Windows 10. Older versions of Windows may or may not work. Newer versions – we’ll have to wait and see.

To help keep the software uncomplicated, the software installation directory is fixed. Data files and output files may be kept anywhere, organized as desired by the user. User skills such as creating directories and navigating the file system are assumed.

□ Create the folder “c:\users\Larry\Arsenic”

□ Copy all files from the DropBox folder:

“Caminos Tech – Project – Arsenic quant/Program Updates and Revisions/Files to be Downloaded”

to the newly created folder.

Online, go to [www.python.org](http://www.python.org/). Download the latest version of Python 3 (3.8 as of 12/1/2019). Make sure to download the 64 bit version. Also, during installation check the box to allow Python to update the Path file on the computer.

Open a command window. This is done by typing cmd in the “type here to search” box on the lower left of the Windows desktop, and selecting “command prompt.” You will be using the “pip” command which Python has installed for you. End each line with a carriage return. If Pip suggests that you update to the latest version, this is recommended – just follow the instructions shown on the screen.

Type the following lines

pip install iorodeo-potentiostat

pip install matplotlib

pip install easygui

pip install scipy

At this point you are ready to go!

From the windows start command, go to “all programs → Python 3.8 → Idle. Note that your installation might be 3.9, or 3.10, or ….

In a few seconds the Idle window will open. This is the PIP GUI. Go to File → Open. Navigate to c: larry\users\Arsenic\ArsenicTest.py. This will open the Python rodeostat program in a new window. Note that you are now in full command of this program; you can run it, inspect it, and/or modify it. Be careful.

In the ArsenicTest.py window, click on “Run → Run Module. Choose temp.ast. This is a demo test program that should run properly. If no rodeostat potentiometer is connected to your computer, the program should print a message “going to simulation mode.”

The file should load, the program should produce some information about the data file, and ultimately produce graphical output


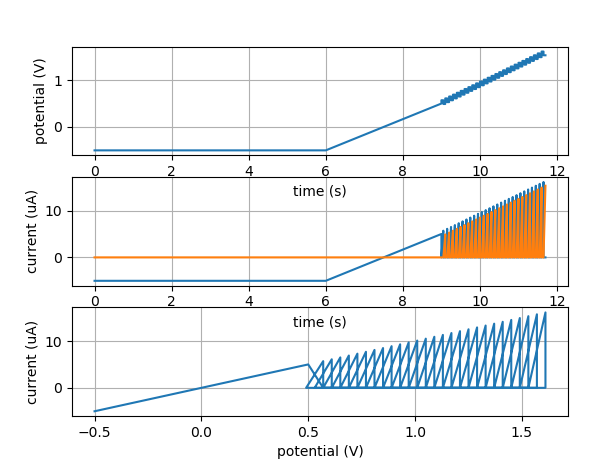


When the graphic window is closed, a dialog box appears asking whether or not to save the data. At this point request can be rejected, the program will close, or the data file can be saved and inspected in a spreadsheet or text editor.

The meaning of this data, the structure of the data file, etc. will be discussed in section V.

To test the gui program operation, double click on “Arsenic App.exe” (in c:\users\larry\arsenic) and the gui should appear. Click on “Open Existing file”. Navigate to the correct folder and then open “temp.ast.” The voltage profile specified by “temp.ast” should appear. Click on “Run Rodeostat w/ Active File” and the Python run should begin, ending with the same graphs as above.

# IV Command set and structure of data files

Note: The data files for the Python and the GUI programs are identical and interchangeable. They are standard ASCII text, Comma Separated Variable files. When being created or modified in a text editor, e.g. Notepad, the user must manually take care of the line formatting. When working in the GUI program, the program takes care of these details. Also, the GUI program offers a drop-down list of “known” commands, in a text editor the user is responsible for entering commands correctly. The command entries are case-sensitive.

Do not create or edit data files in a word processor. It’s not impossible, but it is very difficult.

Data File creation. Data files can have any legal Windows file name, with the extension .ast This can be changed if necessary, but “As Test” seemed a good file extension to use.

When creating a file manually in a text editor, the entire file must be created (every line). When using the GUI, the “create a new file” instruction automatically creates comment lines with the file name and creation date, and the first 2 data lines with typical values inserted. Any or all of this can be changed.

**#** Comment Line:

Ignoring comment lines for the moment,

Line 1 is mandatory: **dt, num1**. For example “dt, 0.05”. This is the time increment, in seconds, for how often the Rodeostat sends / acquires information. All timing information in subsequent commands refer to multiples of dt. Too large a value of dt will cause a loss of resolution. Too small a number can cause the creation of very large output data files and ultimately exceed the Rodeostat capabilities; the Rodeostat will handle .02 seconds, for use of smaller values check with Rodeostat.

Line 2 is mandatory: **PotParams, num1, num2**. For example, “PotParams, 2, 3” The Rodeostat sets itself up in different ranges, a tradeoff between dynamic range and resolution. Num1 is the maximum voltage magnitude that subsequent commands will be accepted from – if you try to send larger, the voltage will be clipped at num1. Num2 is a current range. Allowed entries are

n1 = 1 or 2 or 3 or 4 -> 1, 2, 5, 10 volts

n2 = 1 or 2 or 3 or 4 -> 1, 10, 100, 1000 microamps

The following commands are not mandatory; you can choose which ones you want, in which order, and as few or as many as you want. They will be executed in order. The “time” counter in the software runs continuously – i.e. it does not restart at each command. The commands now are just a starting set and the parameters are a first pass.


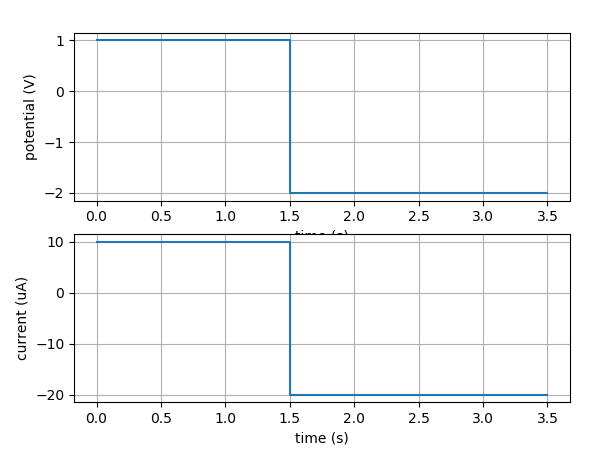
**ConstVolts, num1, num2**: A constant voltage of num1 volts is applied for a duration num2*dt seconds

Example: testFile1.ast

dt, 0.25
PotParams, 2.0, 3
ConstVolts, 1., 6
ConstVolts, -2, 8

Since ConstVolts is often used for a long electroplating step, a timer showing progress is written to the console while it’s running. This is for user convenience only, it doesn’t interact with anything.

**Ramp, num1, num2, num3**: A voltage ramp from num1 volts to num2 volts, over num3*dt seconds


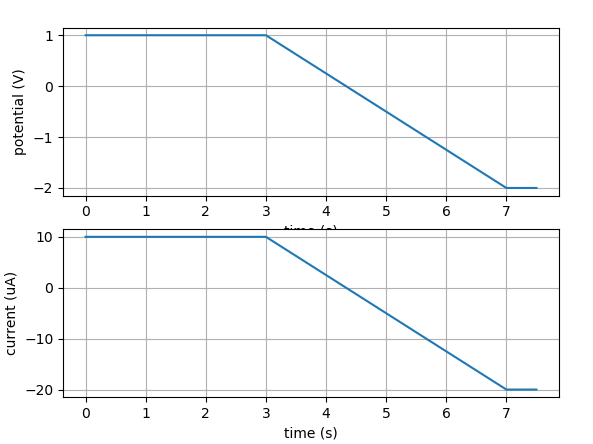
Example: testfile2.ast

dt, 0.5
PotParams, 2.0, 3
ConstVolts, 1., 6
Ramp, 1, -2, 8
ConstVolts, -2, 1

**SquareWave, num1, num2, num3, num4**: A Square wave. High voltage = num1, low voltage = num2. Num3*dt = Full cycle period in seconds; num4 = number of full cycles


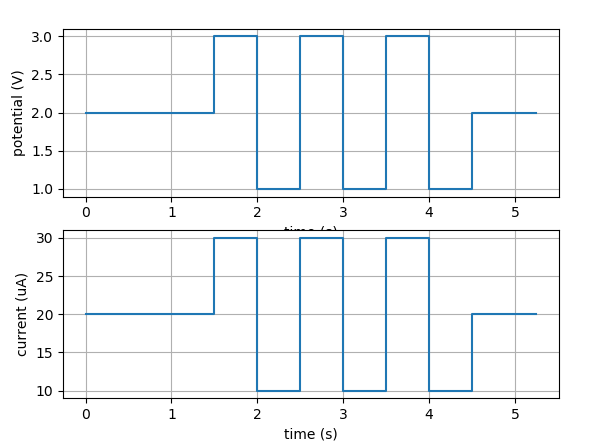


Example: testfile3.ast

dt, 0.25
PotParams, 2.0, 3
ConstVolts, 2., 6
SquareWave, 3, 1, 4, 3
ConstVolts, 2, 3

**SawTooth, num1, num2, num3, num4**. A sawtooth with an average value of num1 volts, + and – peaks num2 volts about the average. Num3*dt = duration of the sawtooth. Num4 = number of total repeats.


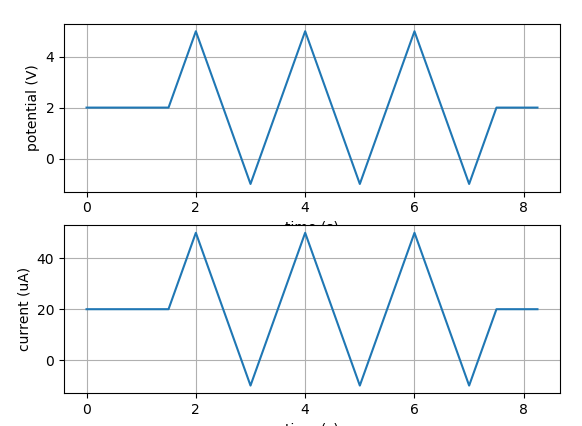


Example: testfile 4.ast

dt, 0.25
PotParams, 2.0, 3
ConstVolts, 2., 6
SawTooth, 2, 3, 8, 3
ConstVolts, 2, 3

**Alert, num 1**

The word “Alert” followed by num1 is printed when the program reaches this command’s location in the data file. This is useful, for example, for noting the end of a plating step.

Notes: **Alert, 2** or **Alert, 3** will produce properly numbered alert messages. This means that several alert messages may be placed in a data file. The only restriction is that an Alert message cannot appear sooner than line 3 in the data file. The Alert command does not affect Rodeostat operation in any way; it’s a message to the user that the program has reached the line of the Alert command and is proceeding.

Example data file: (Comment lines are allowed, none are shown here)

dt, 0.05

PotParams, 2.0, 3

ConstVolts, -1.6, 20

Alert, 1

ConstVolts, -1, 20

**Swas, -.7, .7, .05, .008Swas, num1, num2, num3, num4**. A stepped square-wave profile.

num1 = v_start stripping voltage at start of sweep

num2 = v_stop stripping voltage at end of sweep

num3 = v_amp amplitude of square wave

num4 = v_lad size of ladder voltage step


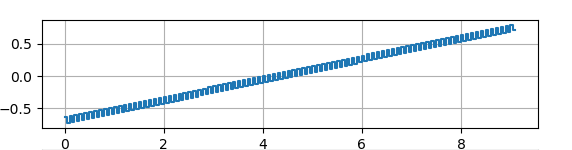
Example: swastest.ast

dt, 0.05

PotParams, 2.0, 3

Swas, -.7, .7, .05, .008

Example: AresenicTest1.ast

dt, 0.05

PotParams, 2.0, 3

ConstVolts, -1.6, 20

ConstVolts, -1, 10

Swas, -.7, .7, .05, .008


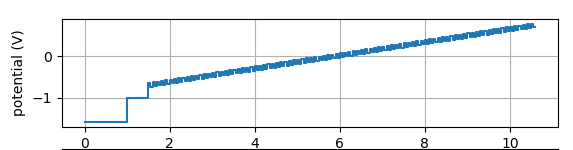


# V. Command Line Operation From Python

Starting the software : Double click the Idle icon on the desktop. Idle is a GUI for the Python interpreter. Go to File – Open. Navigate to the correct folder and choose ArsenicTest.py. If this has been run before, Recent Files is convenient. Note the folder that this program is in – this is where you’ll want to put your data files and where you’ll find any saved output data. The source code will open in a second window. There are two operating modes:

1. Press F5: If the software does not find a connected Rodeostat, it goes to the “emulation” mode. There will be a message that this is happening. This is useful for software development and also for checking what your data file actually creates. Remember that the potentiostat sends voltage data to the electrodes and then measures the resulting current. In the emulation mode, the software emulates a simple resistor, so the current shown carries no useful information. The voltage profile is what would actually be sent to the Rodeostat.
2. For a real run, first connect the Rodeostat to the computer (USB). The software will control the Rodeostat which in turn will send current information back to the software. Press F5. Note that the SerialPort application is only necessary for running the Rodeostat online software. It is not necessary here.

In either case, a standard Windows dialog box will open and you can choose your data file. The run then begins automatically.

When the run is complete, the run results will be shown in on=screen graphics. You can print these graphics if you want to. Close the graphics window to continue. A message box will open asking if you want to save the data. If you do, a standard Windows message box will open, allowing you to pick a file name and storage location. When the run is complete, the Rodeostat output is reset to 0 volts automatically and the program quits.


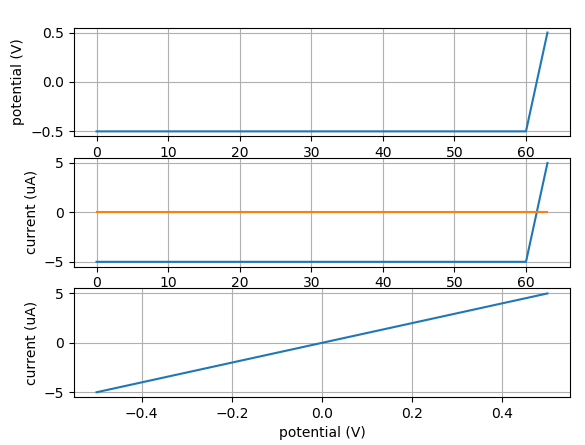
The on-screen graphics is 3 graphs:

1. voltage (volts) vs time (secs). This is the voltage profile described by your data set and sent to the Rodeostat.

2. current (microamps) vs time (secs). This is the current measured ny the Rodeostat.

3. current vs volts. This is useful for a “quick” look at electrode conditioning response. In other situations it is correct nut of no particular use.

When the graphics window is closed, a dialog box appears offering a choice of saving the data file or not. If the choice is made to save the data file, a standard Windows dialog box appears.

The data file is saved as ASCII text, Comma Separated Variable format. It may be opened in Excel, etc. Below is an excerpt from a saved data file

NOTE: The formatting shown below is erratic. This will be corrected.

The variables in each line are time, voltage current and a second current. This second current is only used for square wave voltammetry and will be discussed below.

54.0 , -0.5 , -5.0 ,0

55.0 , -0.5 , -5.0 ,0

56.0 , -0.5 , -5.0 ,0

57.0 , -0.5 , -5.0 ,0

58.0 , -0.5 , -5.0 ,0

59.0 , -0.5 , -5.0 ,0

60.0 , -0.5 , -5.0 ,0

60.0 , -0.5 , -5.0 ,0

60.05 , -0.48333333333333334 , -4.833333333333333 ,0

60.099999999999994 , -0.4666666666666667 , -4.666666666666667 ,0

60.14999999999999 , -0.45 , -4.5 ,0

60.19999999999999 , -0.43333333333333335 , -4.333333333333333 ,0

60.249999999999986 , -0.4166666666666667 , -4.166666666666667 ,0

The following information applies only to data files containing a square wave voltammetry step. In this case, when the program is run and the file is saved, the program will automatically generate a second saved file. This file will have the same name as the chosen saved file name, with **SQ** appended to the end of the file name. This file will is set up for easy Excel graphing. Preparing this file required “assigning” identical time positions to a square wave pulse’s voltage and (2) current numbers, even though they occur at slightly different times. The figure below shows how this is done:


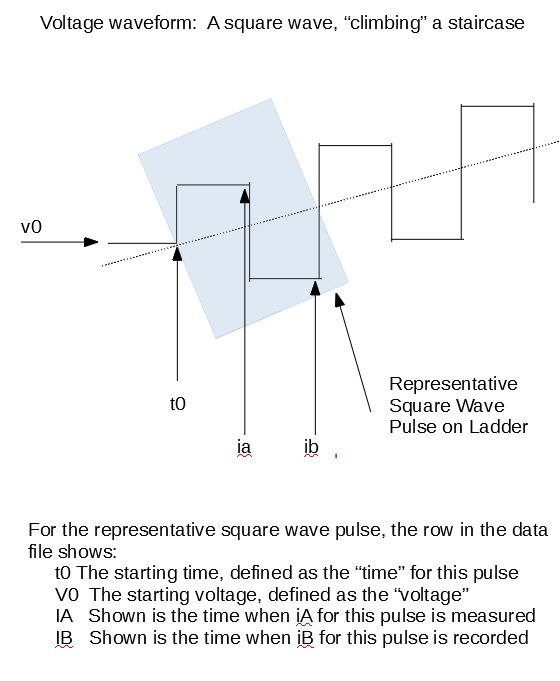


# VI GUI Operation

The GUI is a file creator, editor and viewer for the .ast files described earlier. The UTPUT buttons to run the Rodeostat invoke the Python program and pass the data file to it. All Rodeostat output is as described in the previous section.

The GUI program is started by double clicking on Arsenic App.exe. If desired, a shortcut icon can be created and placed on the desktop.

When invoked, this screen should appear:


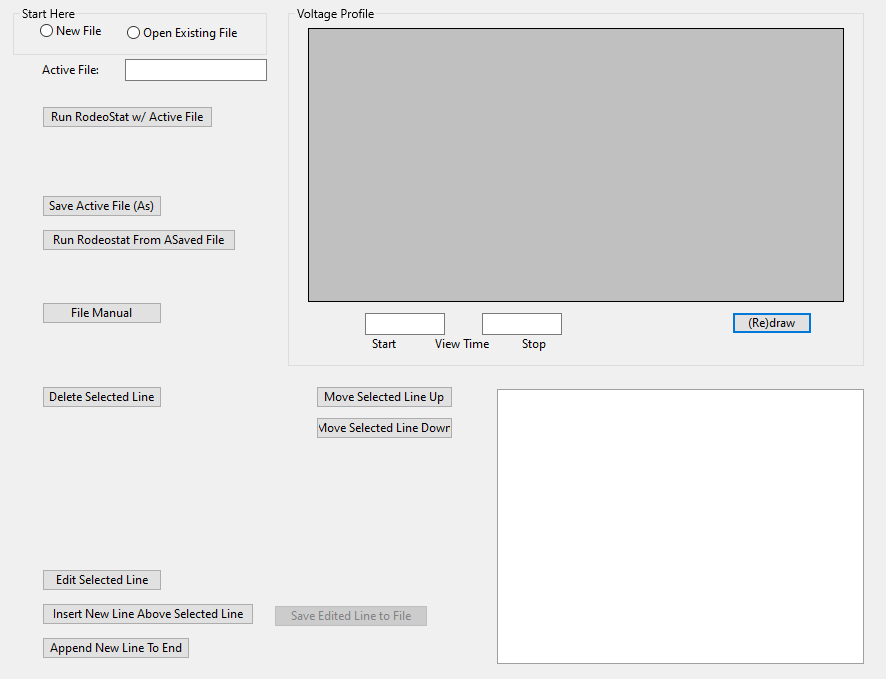


The first choice is to open an existing file or create a


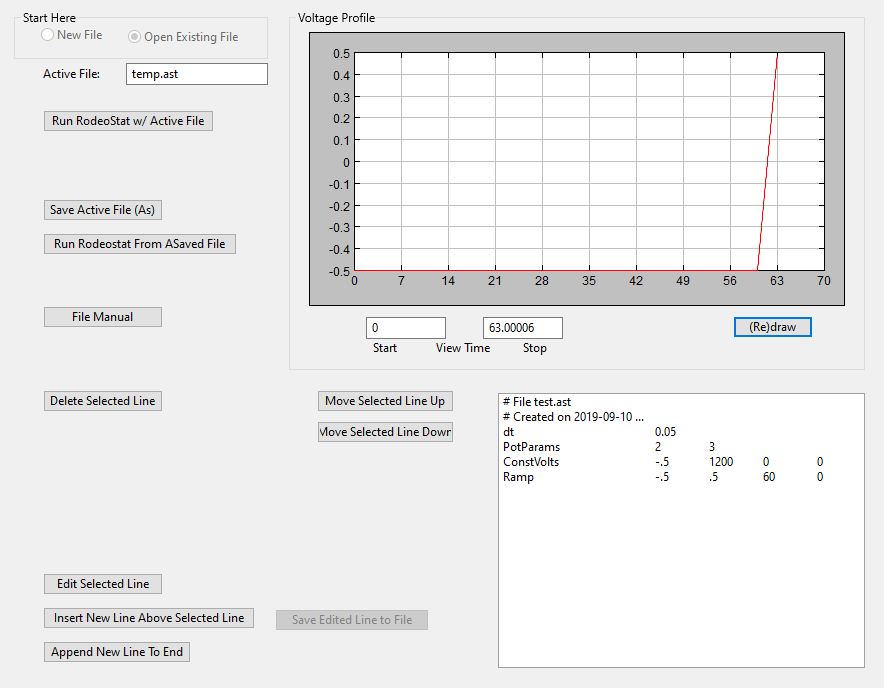


new one. If you open an existing file, it is displayed in the lower right window. The voltage waveform described in the graphics window. It will automaticallly update with changes in the file.. A portion of the waveform may be zoomed into by adjusting the Start and Stop times and clicking on “Redraw”.

If you choose to start a new file, the standard windows dialog opens and you must choose a file name and storage location. A “starter” file is automatically created with typical values. These may of course be changed if necessary.


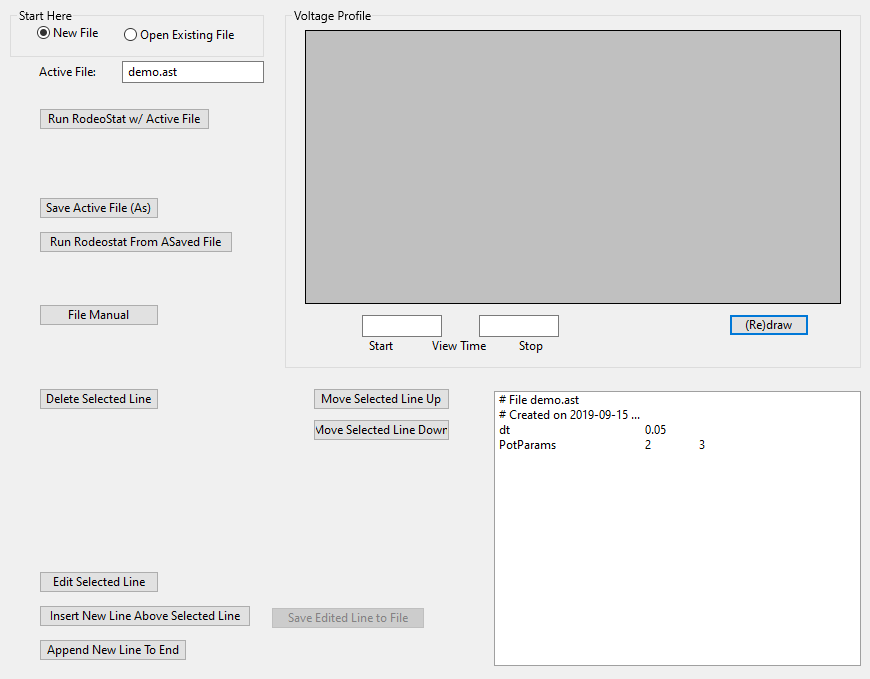


The loaded or newly created file is now the Active File. Note that any editing of this file just changes the contents of this Active File and is not saved to disk until Save Active File is clicked.

An existing file line may be selected by clicking on it,


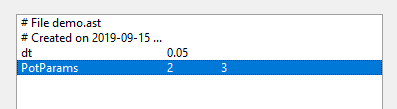


and then clicking on Edit Selected Line. The chosen line is copied to the Edit Data boxes that appear:


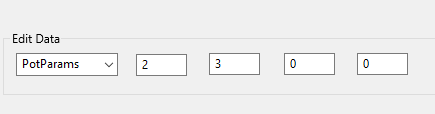


The leftmost Edit Data window has a drop-down list arrow. This list contains all the available command choices. The remaining boxes are nr1, nr2, nr3, nr4 in the parameter sets for each choice.

The File Manual box brings up a window with details of each choice. Remember that different commands require different numbers of parameters. Leave the unneeded boxes blank. Also, don’t worry about the CSV file format, this editor handles it automatically in the background.

Save Edited Line to File will move the edited line back to the file list. Remember that only the Active File has been updated, not the file saved on disk.

The remaining editing choices should be self-explanatory.

When all editing choices have been completed, Run Rodeostat w/ Active File will pass the file to the Python Rodeostat program and the run will proceed as described in the previous section.

# VII. Source Codes

If you choose to edit/update any of the programs, wonderful. However, please rename your revised program(s) and document your changes somewhere.

There are 3 source code files, all in the Files to be Downloaded Folder:

1. Arsenic Test.py. The command Line Python program described in section V.

2. Arsenic TestAutoVersion.py. This is functionally the same as above except there is no prompt for a data file. The data file name is preset and this program communicates with the GUI program.

3. RodeostatFileGensV2.0.xojo_binary_project. This is the Xojo source code for the GUI Arsenic App.exe. This may be viewed, edited and run from the Xojo app (xojo.com). The Xojo app is a free download.
