# Supplementary material for: Low-cost electrochemical detection of arsenic in the groundwater of Guanajuato state, central Mexico using an open-source potentiostat
Source: PLoS One. 2022 Jan 19;17(1):e0262124. doi: 10.1371/journal.pone.0262124 (PMC8769315; doi:10.1371/journal.pone.0262124)
Supplement: S2 File — (ZIP) [file pone.0262124.s002.zip › README.rtf]

In the main folder:
·	The User Manual includes a standard procedure using an older version of the software, given in the folder 'Software version 1'.
·	The data output from the Rodeostat can be visualised and processed to determine the peak height using the Rodeostat_data_processing_template file.
·	Analyte concentrations can be determined using the method of standard additions and the Standard_addition_template file.

The sub-folder 'Software version 1' contains the .py program and the .ast files that were used to perform arsenic measurements using the Rodeostat as per the journal article.
·	The guide to setting up the software has not been updated since originally prepared for internal use.
·	An early GUI for visualising .AST files is also provided.

The sub-folder 'Software version 2' contains updated .py and .ast files, with a significant improvement being incorporation of a relay operation to automatically turn a magnetic stirrer plate and a vibrating motor on and off, during and after deposition.
·	An updated guide to setting up the software is provided.

These files were prepared by Larry Dworsky and Jay Bullen. Future updates to the software and documentation will be provided on the Caminos de Agua website (https://caminosdeagua.org/). Please contact the authors with any questions or comments.
