# Supplementary material for: Low-cost electrochemical detection of arsenic in the groundwater of Guanajuato state, central Mexico using an open-source potentiostat
Source: PLoS One. 2022 Jan 19;17(1):e0262124. doi: 10.1371/journal.pone.0262124 (PMC8769315; doi:10.1371/journal.pone.0262124)
Supplement: S2 File — (ZIP) [file pone.0262124.s002.zip › User Manual - Arsenic detection using the Rodeostat and CdA software.docx]

User manual for electrochemical determination of total As using Rodeostat and a gold
microwire electrode

Jay Bullen at Caminos de Agua, 28/10/2020

# Preparation

## Equipment and instrumentation

- Potentiostat with a potential range of at least +/-2 V (in this case, a Rodeostat, purchased in 2019)
  - Equipped with a gold microwire (e.g. 30 µm diameter) working electrode, a longer and wider diameter iridium or platinum auxiliary electrode (counter electrode) and a Ag/AgCl/3M KCl reference electrode.
  - A vibrating motor attached to the gold microwire electrode*.
  - A controller for the vibrator (we are currently using the Switchbox connected to the PalmSens2, however in the future we would ideally connect the vibrator and stirrer to an Arduino for automated operation).
- Magnetic stirrer plate and magnetic stirrer bar*.
- Mass balance (accurate to at least 1 mg).
- 2x 100 mL volumetric flasks.
- Adjustable pipettes: with maximum volumes of 1 mL and 100 μL.
- pH meter or pH test strips (to validate that samples have been adjusted to the desired pH).
  - pH buffer solutions to calibrate the pH meter where used.
- Designated arsenic waste container (for high concentration stock solutions).
- 4x plastic containers (100 mL volume) for stock solutions and acids. All solutions containing As(III) should be stored in opaque containers to prevent the photooxidation of As(III).
- Squirty bottle with deionised water for rinsing electrodes and cleaning sample cells.
- Plastic funnel.
- Measuring cylinders.

**best results are achieved using both stirring and vibration during deposition, however As detection can be made with only one of these options.*

## Chemical reagents

- Either: As_2_O_3_ or Na_2_HAsO_4_·7H_2_O
  - As_2_O_3_ as a source of As(III) for the detection of As(III) or total As
  - Na_2_HAsO_4_·7H_2_O as a source of As(V) for the detection of total As only
- Deionised water
- 1 M HCl
  - For the acidification of the sample solution
- concentrated H_2_SO_4_
  - For electrode conditioning (cyclic voltammetry)
- *1 M NaOH*
  - *If As_2_O_3_ is to be used to prepare a stock solution of As(III), then NaOH is needed to dissolve the As_2_O_3_*
- *For the detection of As(III):*
  - *Either: hydrazine (e.g. liquid hydrazine, hydrazine hydrate or hydrazinium sulphate salt) or ascorbic acid*

## Preparation of stock solutions

### Arsenic stock solutions

- First prepare 1000 mg L^-1^ stock solutions (100 mL volume).
  - For As(V), add 0.4165 g Na_2_HAsO_4_·7H_2_O to a 100 mL volumetric flask. Add a small volume of 1 M HCl to achieve neutral or acidic pH (0.16 mL will give a pH of approximately 7.8) and make up to 100 mL with deionised water.
  - For As(III), add 0.1320 g As_2_O_3_ powder to a 100 mL volumetric flask. Add a small volume of 1 M NaOH to dissolve the As_2_O_3_. Make up half way with deionised water. Add a small volume of 1 M HCl to adjust to neutral or acidic pH. Make up to 100 mL with deionised water.
- Use these solutions to prepare 1 mg L^-1^ working solutions (100 mL volume).
  - Add 100 μL of the 1000 mg L^-1^ stock solution to a 100 mL volumetric flask and make up to 100 mL with deionised water. (Use a new, clean volumetric flask to avoid cross-contamination).
- Store As stock solutions in the fridge.

### Other solutions

- 0.5 M H_2_SO_4_
  - For electrode conditioning.
  - 98% concentrated H_2_SO_4_ is approximately 18.4 M. So, add 2.72 mL of H_2_SO_4_ and make up to 100 mL using a measuring cylinder (the precise concentration is not so important).
- 1 M HCl
  - For acidification of the samples
  - For a 36% solution of concentrated HCl, add 8.06 mL (8.1 mL using a 10 mL pipette) to 92 mL deionised water for 100 mL total volume.
  - (Concentrated HCl is more typically 32%, in which case 9.8 mL of HCl would be made up to 100 mL using deionised water).
- For the detection of As(III)
  - Prepare either a solution of 10 mM hydrazine to stabilise As(III).
    - Add 56 µL hydrazine monohydrate to 100 mL deionised water.
    - (Alternatively add either 0.130 g hydrazinium sulphate or 31.4 μL of liquid hydrazine to 100 mL deionised water.)
    - Store the hydrazine solution in the fridge to prevent degradation.
  - Or prepare a 10 mM solution of ascorbic acid:
    - Add 0.176 g to 100 mL deionised water. The final pH will be 3-3.5.
    - Store the ascorbic acid in the fridge.

*Note that we did not test ascorbic acid in this study, however the 10 mM hydrazine solution helped stabilise the As(III) and prevent oxidation.*

## Setting up the voltammeter

- Assemble the vibrating motor with the working electrode (if needed).
  - To couple the working electrode with the vibrator, use the following steps. Firstly, trim two 1 mL pipette tips so that they fit inside one another, and so that they sit within or on-top of the working electrode port (the hole in the sample cell lid). Fit the vibrator inside the central pipette tip, securing in place using tape of parafilm. Slide the working electrode between the two pipette tips, again securing in place using tape of parafilm.
- Assemble the Rodeostat.
  - Connect the Rodeostat to the electrodes.
    - Using the electrically shielded cable, connect each coloured pin to the Rodeostat as shown. Use a small flat head screwdriver (one is supplied with the Rodeostat) to tighten and loosen the screws.


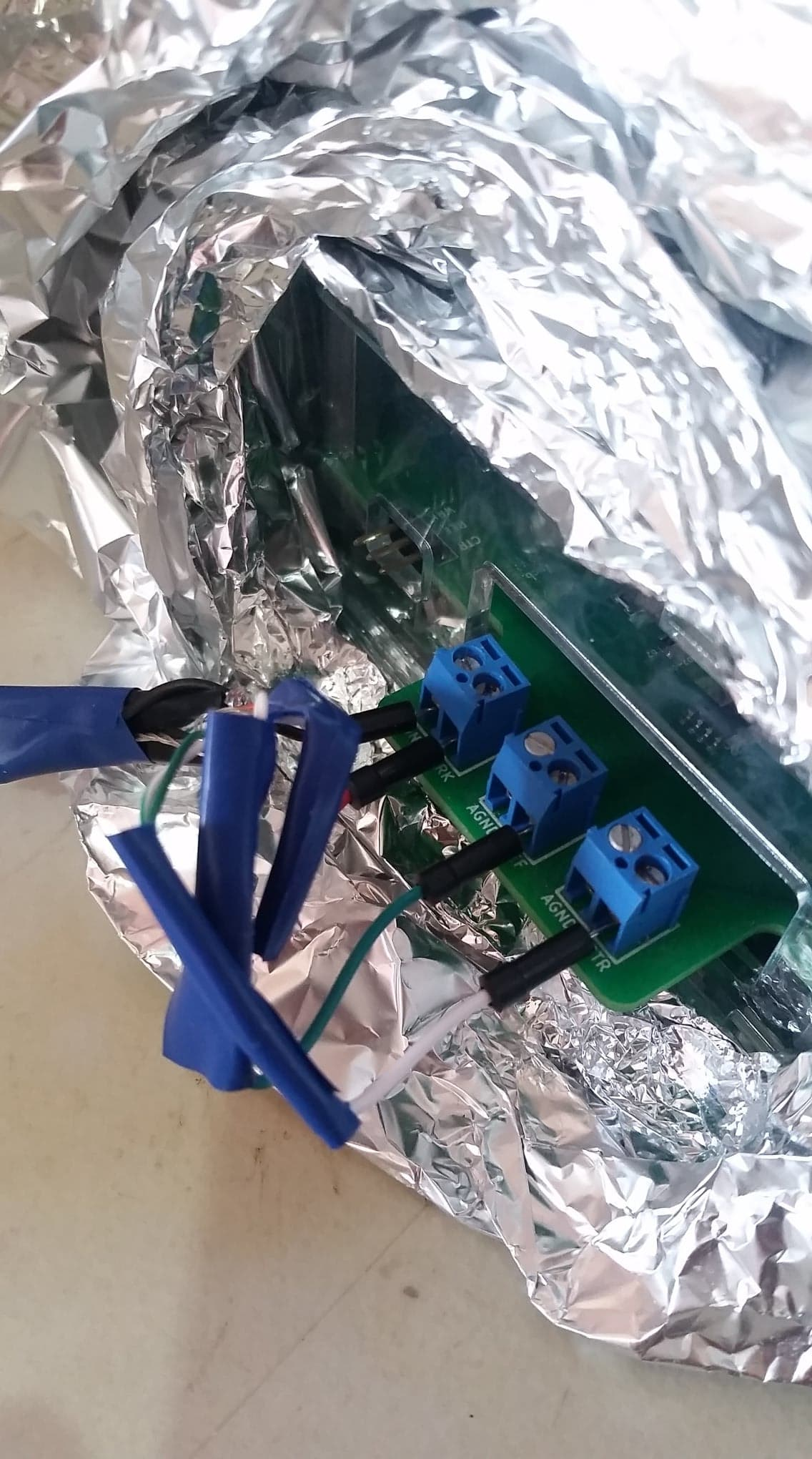


counter/auxiliary

reference

working

ground

- - - The grounding wire can be connected to any electric shielding foil or Faraday cage. This should be connected to one of the ports labelled *AGND* on the Rodeostat. We chose to connect it to the grounding port adjacent to the working electrode. (This will theoretically reduce electrical noise, but we have not measured the effect).
      - Our current shielded cable (the modified ethernet cable) has all the grounding wires connected together. Elsewhere, each electrode may have its own grounding wire and all three may be connected to separate grounding ports on the Rodeostat, using the remaining grounding ports.
    - Using the colour coding to identify which cable to use, connect the working, auxiliary and reference electrodes as shown.
    -
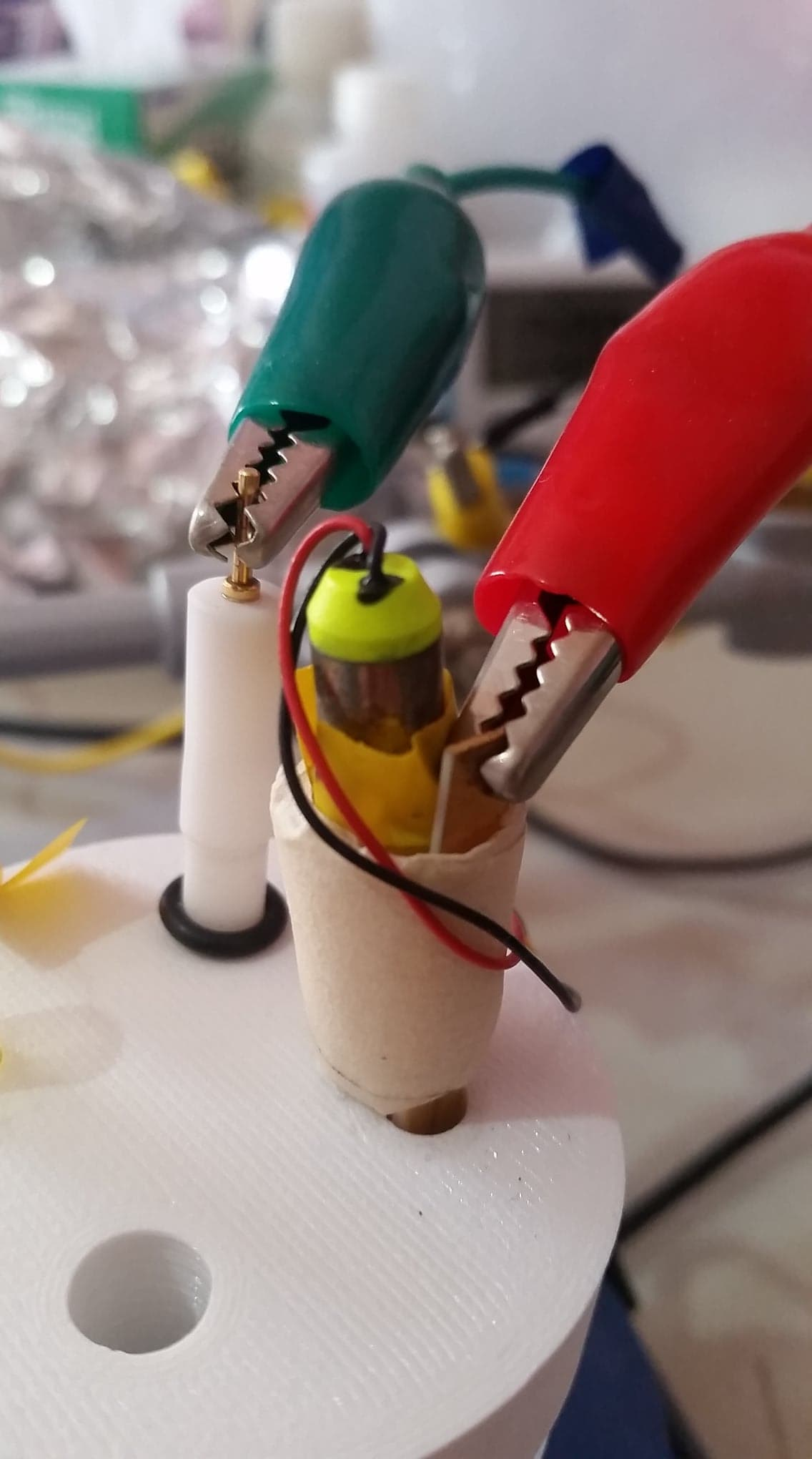


connected to *WRK* port on Rodeostat

working electrode sandwiched between two cut pipette tips

vibrator wedged inside central pipette tip

reference electrode

connected to *REF* port on Rodeostat

- - -
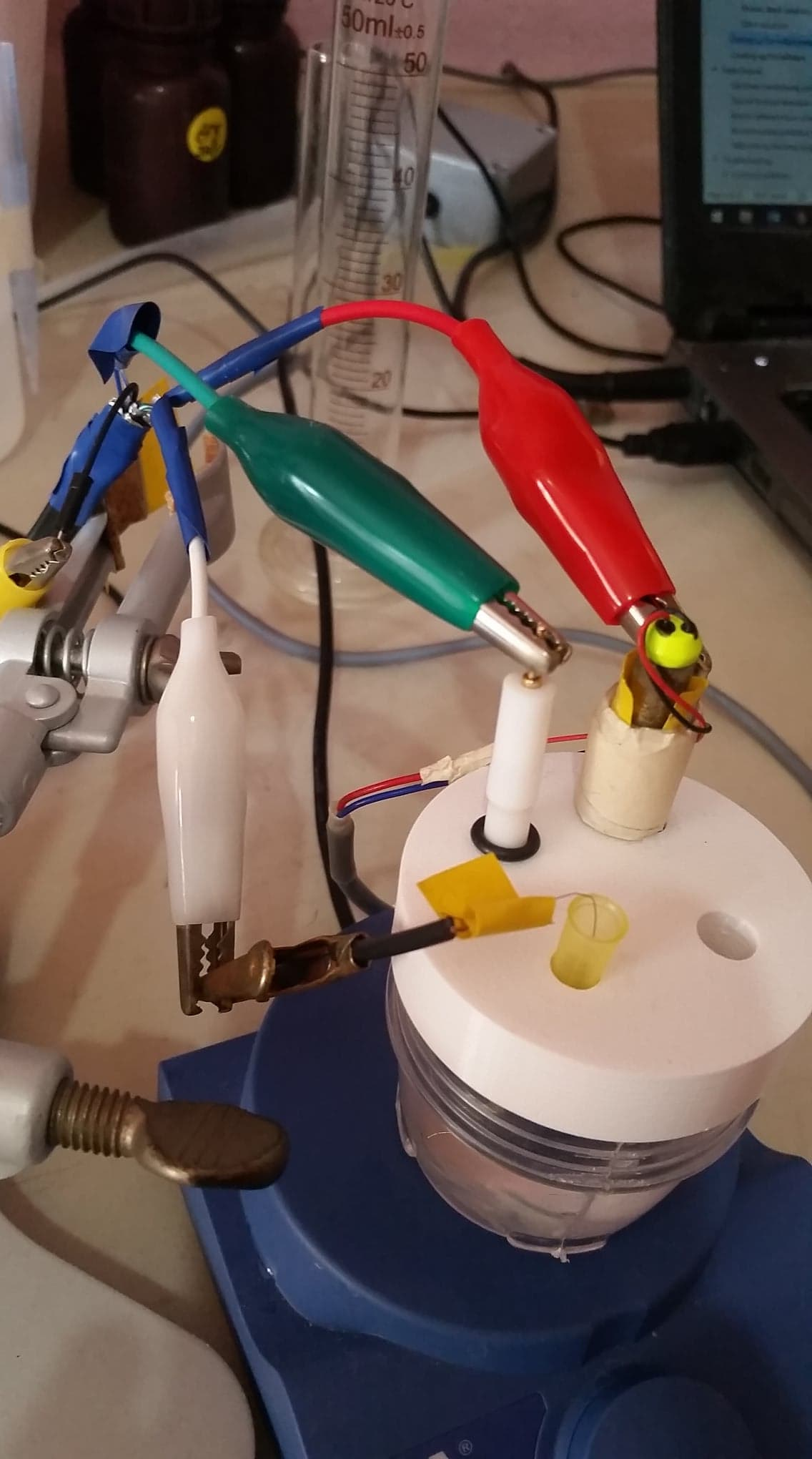


wrapping the single wire of the auxiliary electrode with copper cabling

connected to *CTR* port on Rodeostat

iridium wire auxiliary electrode

- - - - We connected the auxiliary electrode to some copper cabling terminating in a crocodile clip, as the single wire of the auxiliary electrode is too thin to be directly secured using a crocodile clip (potentially causing an inconsistent connection when the vibration is switched on during deposition). The auxiliary electrode should last a long time, so a better solution will be to solder the auxiliary electrode to the copper cabling, instead of using electrical insulating tape.
    - Connect the grounding cable to any extra shielding, such as the aluminium foil wrapped around the Rodeostat as shown. (This will theoretically reduce electrical noise, but we have not measured the effect – it is likely more important to shield the wires that connect the electrodes to the Rodeostat than the Rodeostat itself).
    -
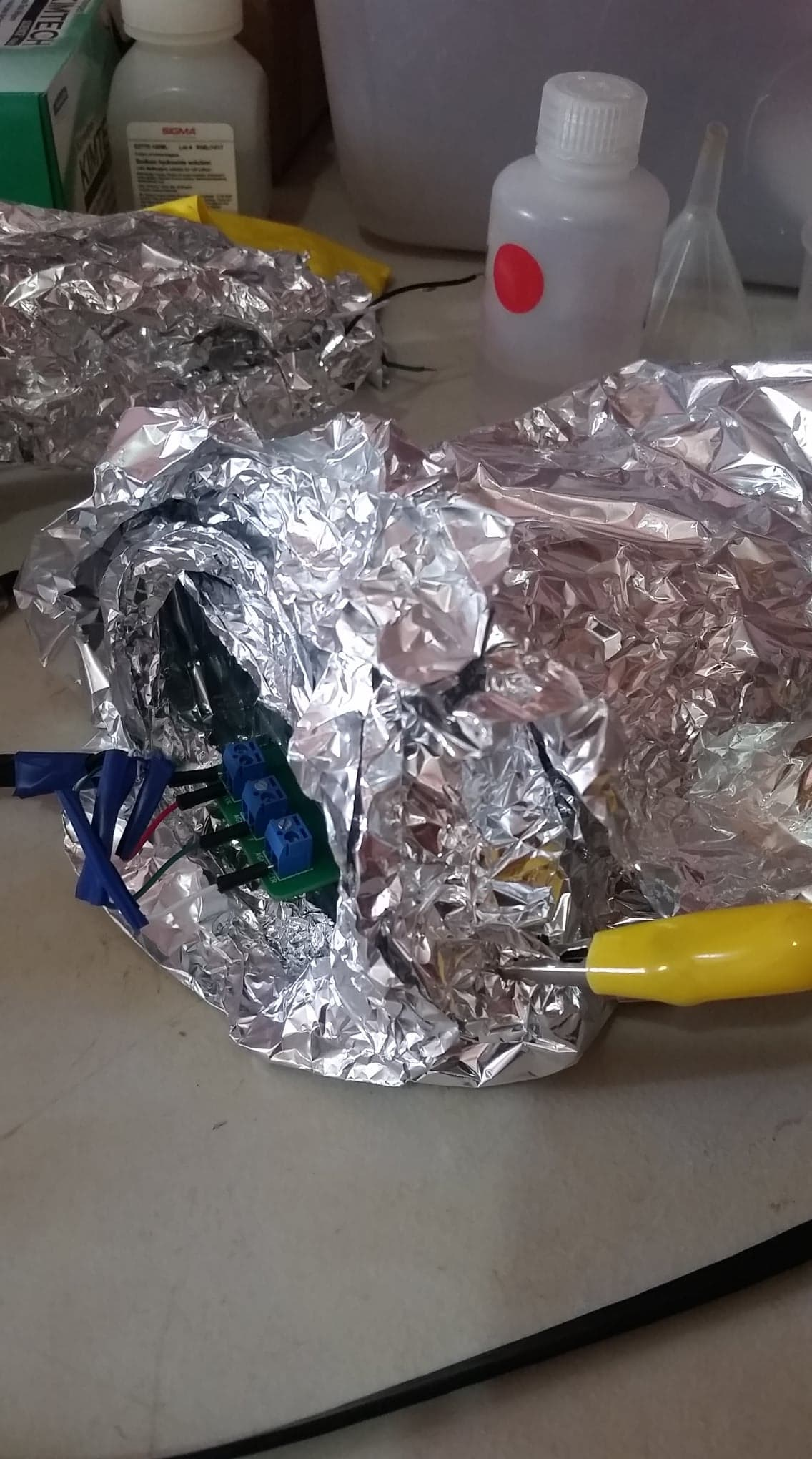


aluminium foil for shielding

connected to ground

- - Connect the PalmSens to your computer via the USB cable.

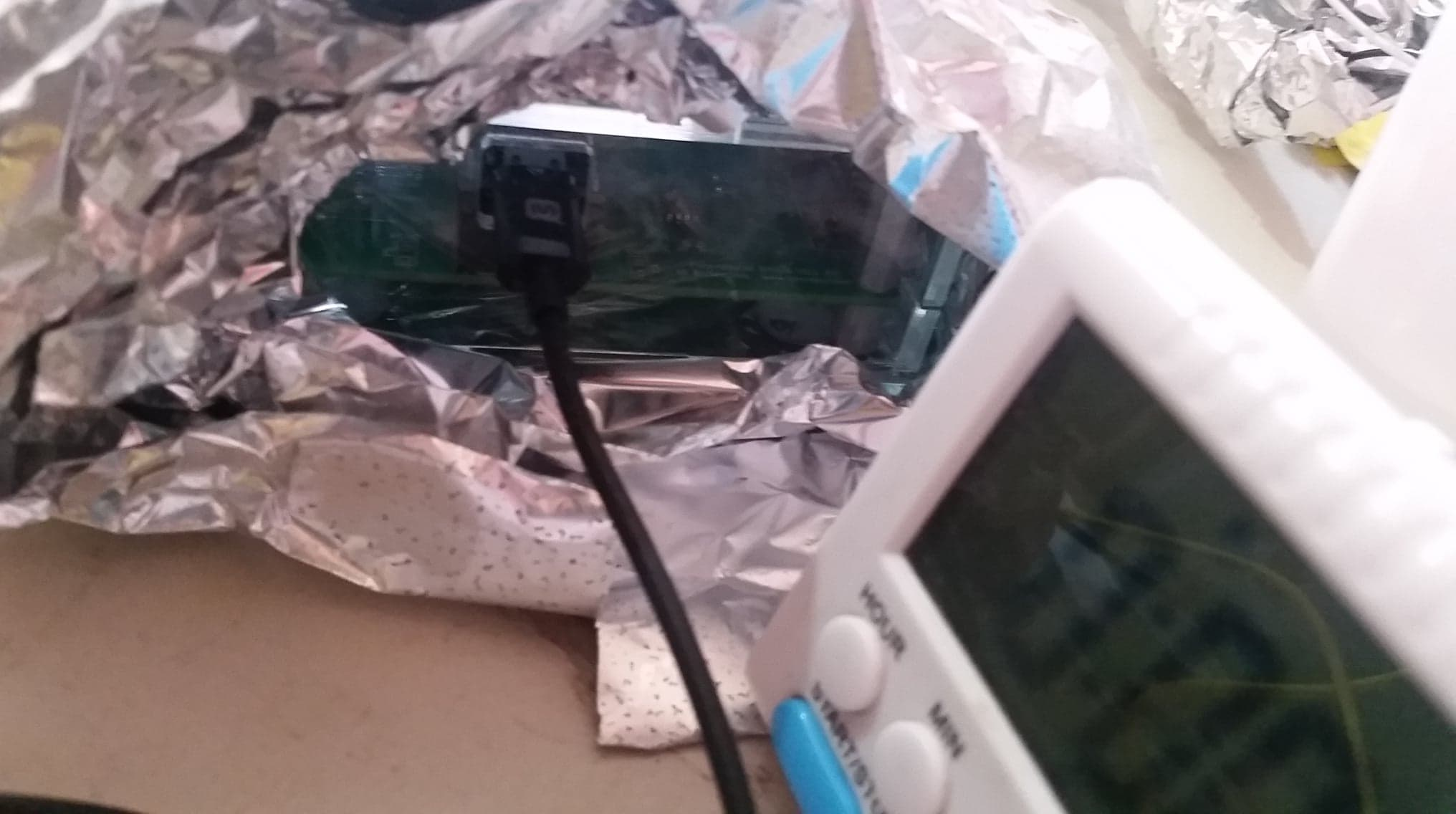


USB port

- - When performing your measurement, all electrodes should be submerged in solution. This includes the entirety of the gold working electrode (i.e. all of the gold microwire).
  - The following figure shows the three electrodes, from left to right: iridium wire auxiliary electrode, Ag/AgCl reference electrode, gold microwire working electrode.
    -
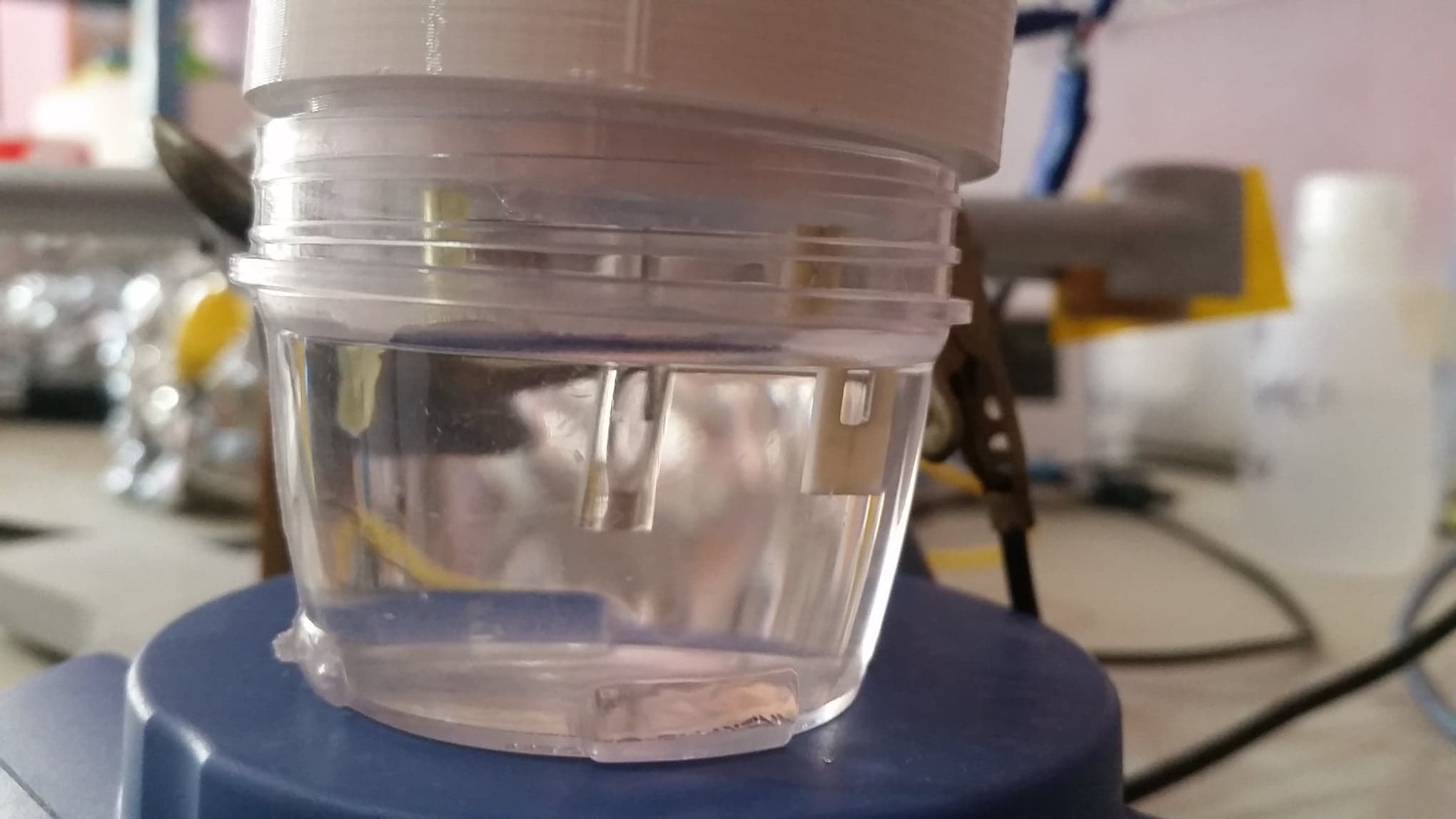

    -
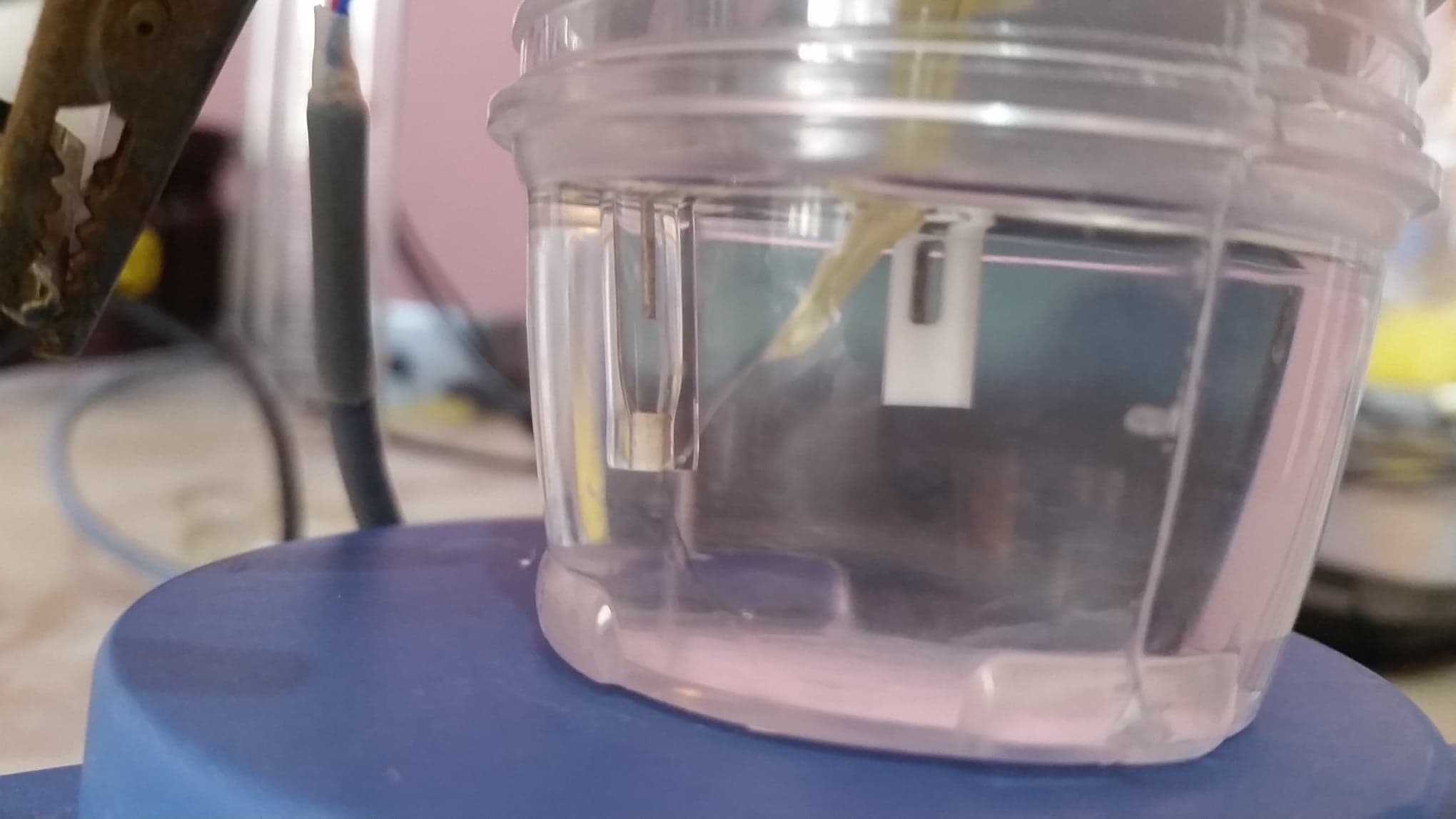

  - When stirring magnetically, take care to ensure that the stirrer does not collide with the electrodes. You can raise the electrodes above the stirrer, or position them to the side of the sample cell.

## Setting up the software

- Please note that this user guide uses the software provided in the folder ‘Software Version 1’. An updated version of the program allows for automated control of stirring and vibration. For more complete instructions on setting up the software, please see the documentation.
- Download and install the latest version of Python (<https://www.python.org/downloads/>)
  - Open the command prompt in windows (search for “cmd” in the start bar)
  - Enter the following lines, pressing the return key (enter) after each:
    - pip install iorodeo-potentiostat
    - pip install matplotlib
    - pip install easygui
    - pip install scipy
- You can run our homemade software by simply double clicking on the program file.
- However, you can also run the software from IDLE, which is Python’s “integrated development environment”. This allows us to keep all of the print outs to review later.
- To do this, open IDLE (found in the Python folder of the start menu).

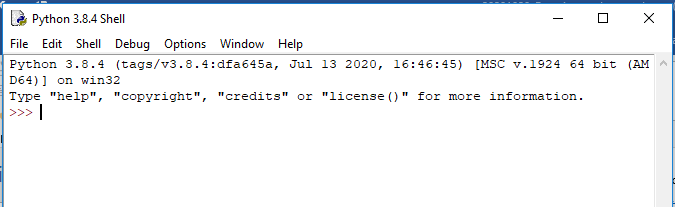

- Then click File 🡪 Open, browse to the correct folder and open the most recent version of our homemade software (the filename will be different, but it will be a .PY file).

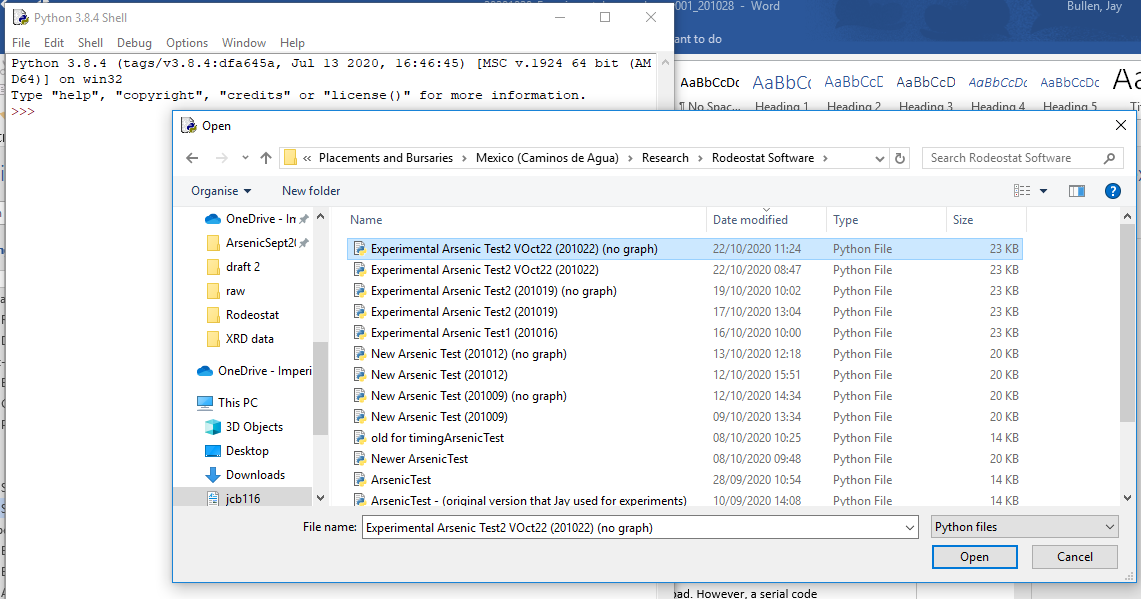

- A second window will open, displaying the program’s code.

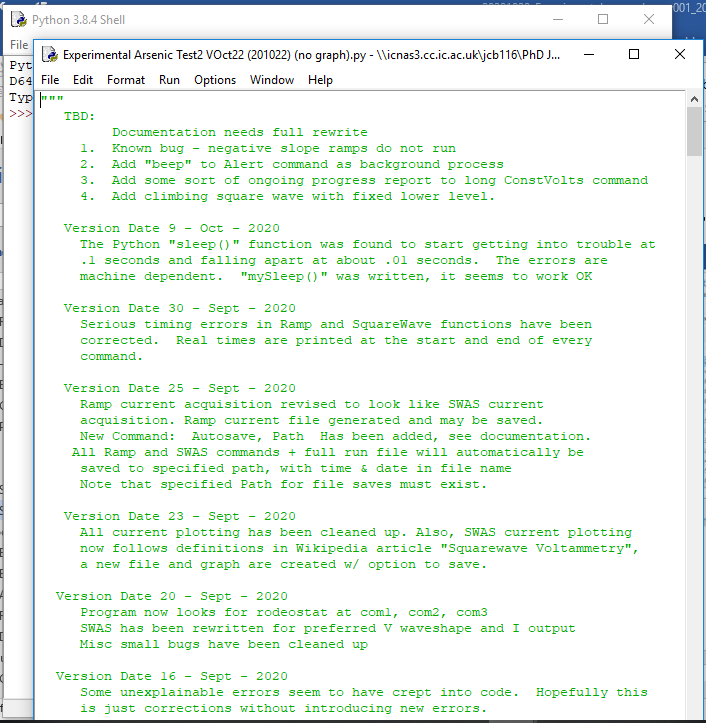

- To run an experiment, we open a *.AST* file. To do this, click on Run 🡪 Run Module

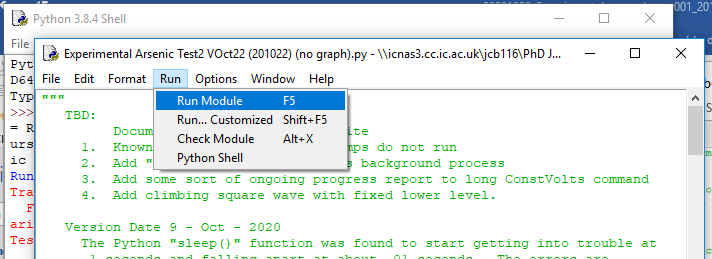

- You will be prompted to open a .AST file. Open the .AST file corresponding to the measurement or experiment that you wish to perform.
- If the Rodeostat is not connected to your computer via USB, then you will see the message “Com port not found – going to emulation mode”.
-
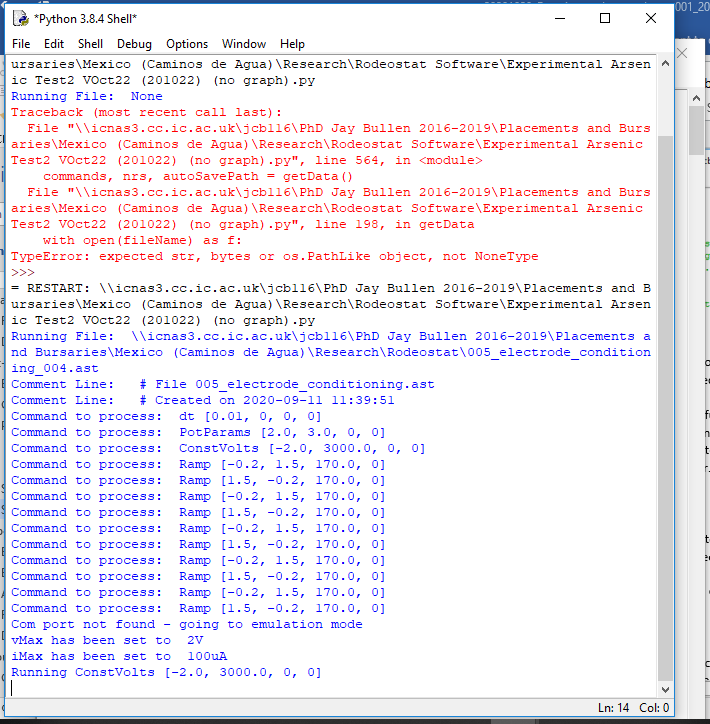

- If you see this message, then try reconnecting the USB cable and running the .AST file again.
- If the Rodeostat is still not identified by the program, then go into the Device Manager (type “Device Manager” in the start menu). You should see the device listed as “USB Serial Device” in the “Ports (COM & LPT)” sub-menu, and the USB Serial Device should be assigned a COMPORT number (in this case, COM3).
-
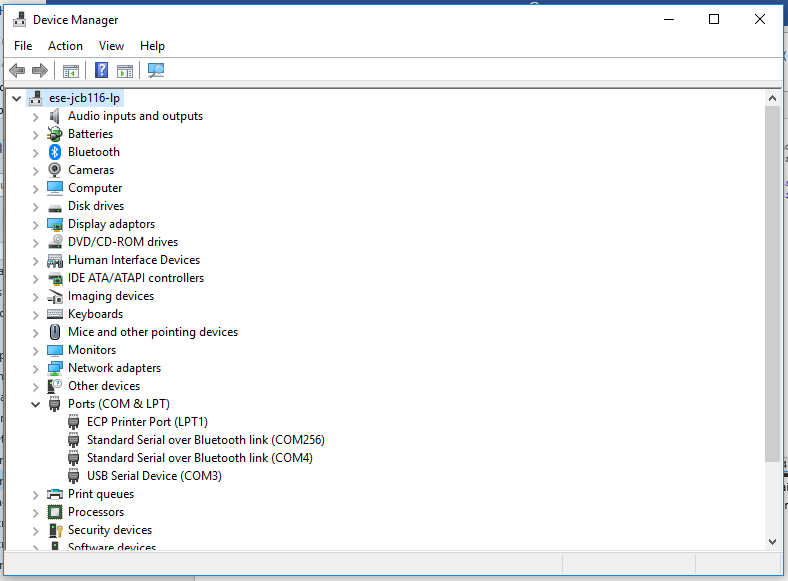

- The current version of the program searches for a Rodeostat connected to the computer via USB with a COMPORT number between 1 and 8 (we believe). However, older versions of the software would only look for a Rodeostat connected to COMPORT 3. If you are having problems, then you may need to change the COMPORT number of the USB device.
- You can do this by right clicking on the USB device, and going to Properties 🡪 Port Settings 🡪 Advanced. Change the COMPORT number from the drop-down menu and click *Okay*.
-
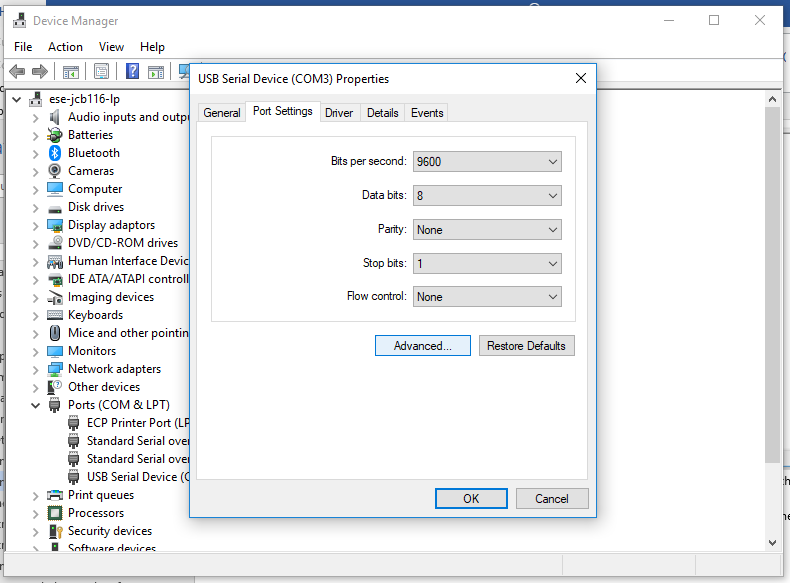

- If the program detects the Rodeostat, then you will see the message “Found pstat at ComX” where X is the number of the com port, when you run the .AST file.


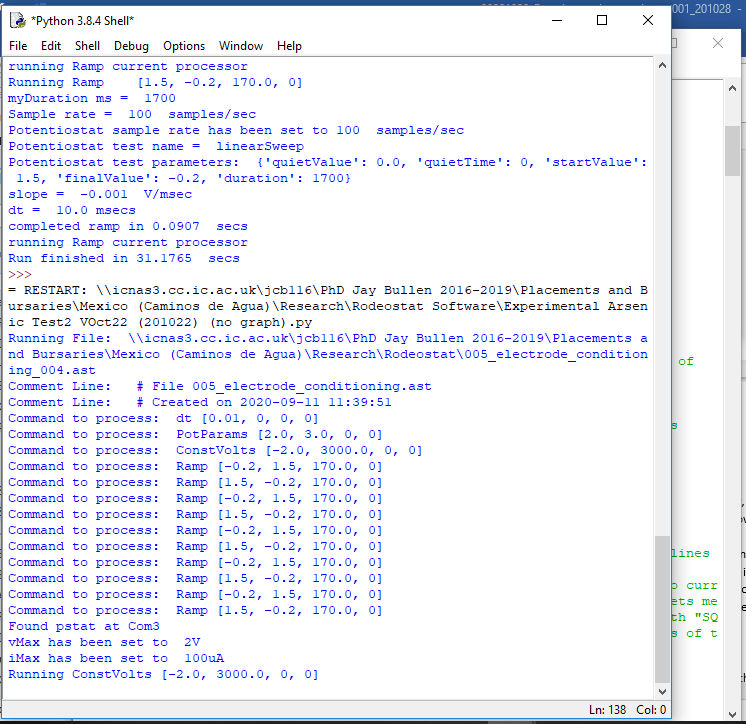


- The print outs before this line repeat the input commands requested in the .AST file.
- Following this, the software will repeat each command as it is run. It is important to keep an eye on the print outs, if you need to perform any manual operations during the measurement, such as switching the stirrer and vibrator on and off, or lifting printed electrodes out of solution to dissipate any bubbles.
- After all commands in the .AST file have been completed, the results will be displayed as a figure.


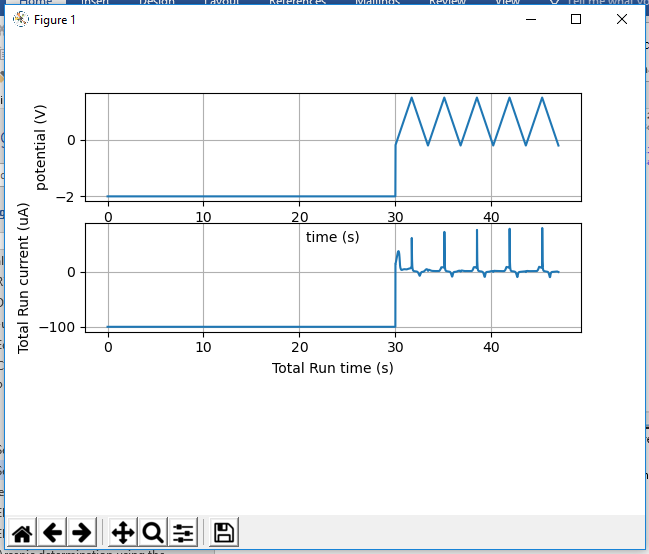


- When you close the figure, you will be prompted to save the data. Click *Continue* to save the data and enter a file name. We save our data files with .dat as the file extension.
- We provide a Rodeostat Data Processing Template spreadsheet in Microsoft Excel which you can use to visualise your results, perform blank subtractions, and measure the peak height.
- When you copy and paste the data file into the Microsoft Excel spreadsheet template, you will have to select Data (from the toolbar) 🡪 Text to columns 🡪 Delimited 🡪 Comma, so that the data cells are pasted into separate columns.
- We also provide a standard addition template spreadsheet, which will allow you to calculate the concentration

# Experimental

## Electrode conditioning using cyclic voltammetry

We use cyclic voltammetry (CV) in 0.5 M H_2_SO_4_ to condition the electrode. This is used (i) to check that the system is set up correctly, (ii) to check that the working electrode is still in good condition, (iii) to clean the electrode of any adsorbed impurities or residual As(0) and (iv) to ensure a consistent surface prior to arsenic measurement, to improve the reproducibility of our results. We recommend performing a CV scan (a) at the start of each day, or at least each time you start performing measurements again after a period of inactivity, (b) when changing electrodes, (b) when you are seeing unexpected results in the data.

- Submerge the electrodes in a sample cell filled with approximately 40 mL of 0.5 M H_2_SO_4_.
  - We reuse the same 0.5 M H_2_SO_4_ solution for multiple experiments, since the primary aim is for electrode cleaning. We will replace the solution only when the CV becomes dirty with peaks corresponding to impurities.
- Turn on the magnetic stirrer, to improve mass transport and prevent bubbles from adhering to the surfaces of the electrodes. The lowest speed on the stirrer plate is normally sufficient.
  - Turn on the vibrator also, if you have access to the vibrator.
- Open the electrode conditioning .AST file using Notepad. It should look something like the following:
  - - *dt, 0.01*
    - *PotParams, 2, 3*
    - *ConstVolts, -2, 3000*
    - *Ramp, -0.2, 1.5, 170*
    - *Ramp, 1.5, -0.2, 170*
    - *Ramp, -0.2, 1.5, 170*
    - *Ramp, 1.5, -0.2, 170*
    - *Ramp, -0.2, 1.5, 170*
    - *Ramp, 1.5, -0.2, 170*
    - *Ramp, -0.2, 1.5, 170*
    - *Ramp, 1.5, -0.2, 170*
    - *Ramp, -0.2, 1.5, 170*
    - *Ramp, 1.5, -0.2, 170*
  - *dt* indicates the sleep time in seconds, determining how quickly the software sends instructions to the Rodeostat. 0.01 indicates that the software will sleep for integer multiples of 0.01 seconds, or 10 mliliseconds.
  - *PotParams* indicates which voltage range and which current range we want the Rodeostat to run at. You only need to change these if you need to use potentials outside the range of -2 to +2 V, and if the current is outside your measurement range (only likely if the electrodes are malfunctioning).
  - *ConstVolts, -2, 3000­­* instructs the Rodeostat to apply a potential of -2 V at the working electrode for a duration of 3000*dt. Since dt is 0.01, this equals 30 seconds.
  - *Ramp, -0.2, 1.5, 170* runs a linear sweep (really a staircase sweep) between -0.2 and +1.5 V, with the potential increased and the current sampled every dt seconds (0.01 seconds), with 170 data points to be collected.
- Consequently, this .AST file will perform (1) electrode conditioning/deposition at -2 V for 30 seconds, then (2) 5 repeat cyclic voltammetry sweeps between -0.2 and +1.5 V, with a sample rate of 100 Hz and a scan rate of 1 V s^-1^. The sample rate is simply one divided by dt.
- Open the program using Python IDLE.
- Run the electrode conditioning .AST file
- Check that the Rodeostat has been found by the software by looking for the line “Found pstat at ComX”.
- Wait for 30 seconds as the working electrode is held at -2 V. When the CV scans start, you will see a print out instructing you that the Ramp is starting, and you will likely see a progress bar indicating the progress of each ramp.
  - **Turn off the stirrer and vibrator when the ramp starts**, so that they do not influence the measurement. (It is okay to turn them off during the first of the 5 repeat CV scans, since we normally just take an average of CV scans 2-5, due to the common presence of impurities in the first of the five scans.


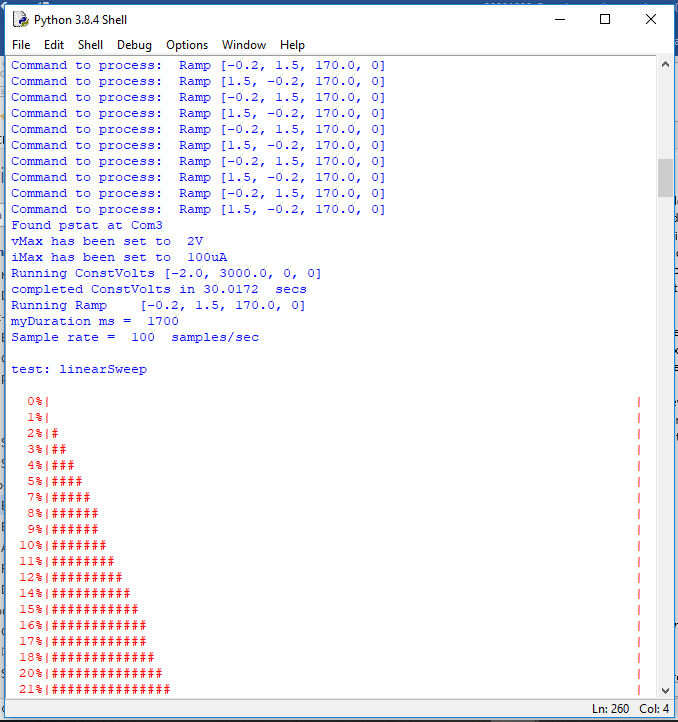


- The software should only display a graph after all 5 CV scans (10 individual Ramps) have been completed. If the software displays a graph after every individual ramp, then either add an autosave command to the start of the .AST file, or run the “(no graph)” version of the software instead.
- After all 5 CV scans are completed, a final graph will be displayed. Close the graph and click *continue* to save the data as a .dat file.
- Open the Rodeostat data processing template file and save it with a new name. Copy the CV spreadsheet template as a new worksheet and give it a new name.
- Open the .dat file using WordPad. You see three or four columns of data. The first column is time (seconds), the second column is the potential of the working electrode (V) and the third column is current (μA). If there is a fourth column of data, this is only there for more advanced measurements such as square wave anodic stripping voltammetry (SWASV).
- Copy and paste the data into Microsoft Excel in column B, where instructed to. Use Data 🡪 text to columns 🡪 delimited 🡪 comma to separate the data cells into discrete columns.
- The two graphs should update with the new data.
- It is common for the 1^st^ scan to be different from the next four scans

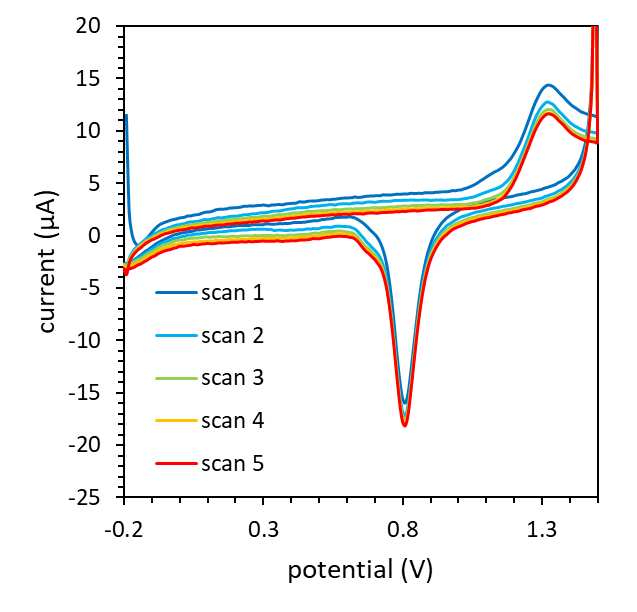

- Consequently, we normally like to just take an average of scans 2-5 and record this data

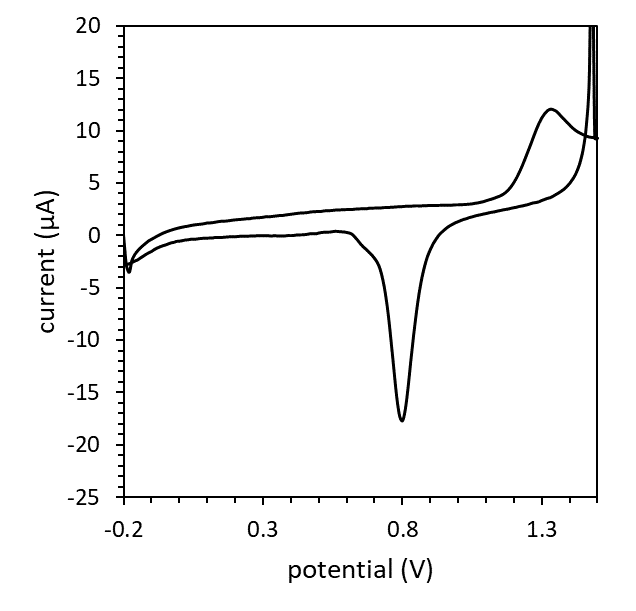

- The ideal CV scan will have two peaks, a positive current at ~+1.3 V in the oxidation scan, and a negative current at ~+0.8 V in the reduction scan. These peaks correspond to the oxidation and reduction of gold at the surface of our microwire working electrode. Above +1.5 V we will see an exponential increase in current corresponding to the oxidation of water, and below -0.2 V we will see an exponential decrease in current corresponding to the reduction of water.
- The spreadsheet will try to determine the position and peak height of the gold oxidation and reduction peaks. It’s a good idea to write these down in your lab book.
  - If the peak positions are more than 0.1 V from their normal position, then you have a problem. Perhaps another metal component is in contact with the solution or perhaps you connected the electrodes the wrong way around.
  - If the peak heights/peak intensities are much larger than normal, then perhaps another metal is in contact with solution.
  - If the peak heights/peak intensities are much smaller than normal, then perhaps your electrodes will be less sensitive and require cleaning/conditioning/replacing.
  - If the CV shape is very wide in the middle, this can indicate a malfunctioning electrode (e.g. with too much crud stuck to the surface).
- You can try replacing the working electrode if you have very unexpected results.

## Electrochemical detection of total arsenic (tutorial)

- We will first measure the blank solution (to check that there is no arsenic contamination), then add 5 µg L^-1^ As(V) and look for the new peak.
- Prepare a blank solution of 0.1 M HCl to give pH pH 1 (total volume 40 mL).
  - Add 1 M HCl (4 mL using a pipette) to deionised water (36 mL using a measuring cylinder).
  - Stir magnetically for 10-20 seconds to mix.
- Submerge the electrodes in the sample solution.
- Check the total As detection .AST file. You should see something similar to:
  - - *dt, 0.01*
    - *PotParams, 2, 3*
    - *ConstVolts, 0.7, 500*
    - *ConstVolts, -1.3, 2000*
    - *ConstVolts, -0.4, 1000*
    - *Ramp, -0.4, 0.7, -917, 240*
    - *ConstVolts, 0.7, 500*
    - *ConstVolts, 0.7, 500*
    - *ConstVolts, -1.3, 100*
    - *ConstVolts, -0.4, 1000*
    - *Ramp, -0.4, 0.7, -917, 240*
    - *ConstVolts, 0.7, 500*
- The procedure is:
  - Cleaning at +0.7 V (5 seconds)
  - Deposition at -1.3 V (20 seconds)
  - Hold at -0.4 V (10 seconds)
  - Linear sweep from -0.4 to +0.7 V (1.2 V s^-1^ sweep rate and 240 Hz sampling frequency)
  - Cleaning at +0.7 V (5s)
  - The procedure is then repeated, albeit with 1 second deposition only, for the background scan.
- -917 means that the sweep will be performed in 0.917 seconds (equivalent to a 1.2 V s^-1^ sweep rate). 240 Hz was chosen as it achieves high potential resolution (x-axis resolution) and should minimise noise from the 60 Hz mains frequency in Mexico. (You can adjust your sampling frequency if the frequency of the mains electricity in your country is not 60 Hz).
- Turn the stirrer and vibrator on.
- Run the total As detection .AST file.
- Watch the print outs and be sure to turn off the stirrer and vibrator during the hold step at -0.4 V (after the deposition step and before the LSV ramp).
- When the ramp is completed, turn the stirrer and vibrator back on.
- Repeat this for both the 20 second analytical scan and the 1 second background scan.
- When the measurement is complete and the results are displayed as a graph, close the graph and save the data as a .dat file.
- Copy the As detection template worksheet and give it a new name.
- Paste the .dat file in the blank electrolyte column, starting at column B.
  - You’ll see that column F subtracts the 1 second scan from the 20 second scan. This is repeated in the columns used for sample analysis.
- Check the graph of the blank electrolyte scans. We want to make sure that the current at -0.4 V isn’t too negative (when using the microwire electrode). Our system seems to converge with ~-5 μA at -0.4 V, when stable. However, when the negative current is ~-20 μA at -0.4 V, As analysis is more challenging. Also check that repeat scan of the electrolyte are stable.

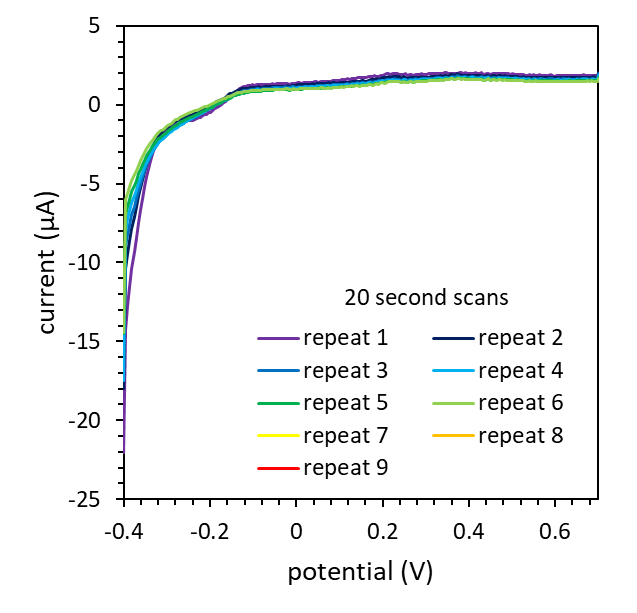

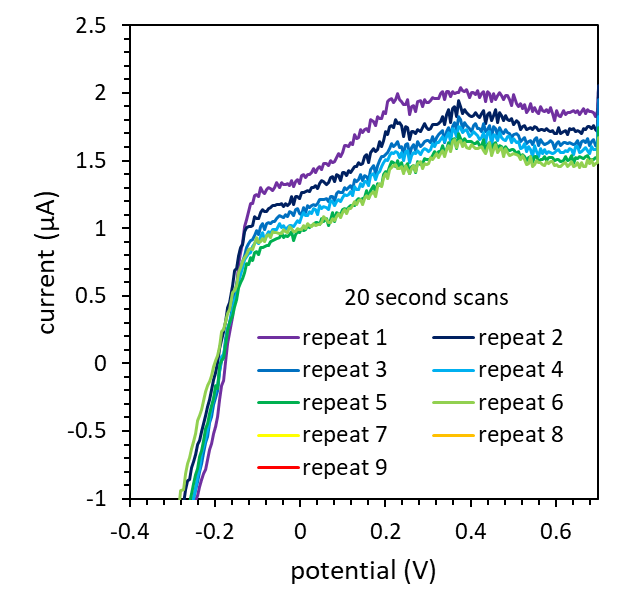

- If you are not happy with the blank electrolyte, then try performing repeat scans to see if the system converges once “warmed up”.
  - Simply paste the data from repeat scans immediately below the last data. The spreadsheet is set up for up to 9 repeat measurements.
- You can also check the blank for impurities. For instance, here we can see the interference at ~+0.22 V, related to the condition of the electrode. Here, the interference has a peak height of ~0.1 μA. The interference is providing a few 10 nA of current at +0.1 V, where we anticipate the As peak to be. This can help us gain an idea of how much As we need to ensure that the +0.22 V interference isn’t a problem in our analysis, e.g. we might want to aim for 0.2 μA for the As peak.
  - We can also see the copper peak at +0.35 V. This peak is too far away and too small to be an interference.
-
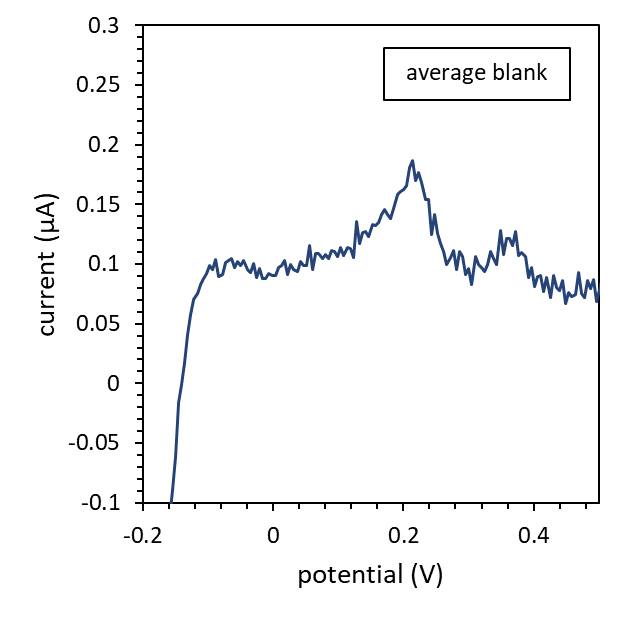

- If you have blank data, then this average blank voltammogram will be subtracted from all sample analyses, to try and remove some of the background current and the impurity peaks from our results. If you don’t have blank data (i.e. the original sample was very low in As concentration, so you have to use a large volume of the sample), then the data will simply be processed without this blank subtraction.
  - Although you could use data from a blank electrolyte collected in a separate experiment.
- Add 5 μg L^-1^ As(V) to the sample cell. This will be 200 μL of the 1000 μg L^-1^ As(V) standard solution if you have a total volume of 40 mL.
- Run the .AST file with 3 repeat measurements.
- Paste the results into the columns provided for the sample.
- You should see an As peak at ~0.1 V. This already has the 1 second background scan subtracted (as well as any blank subtraction).

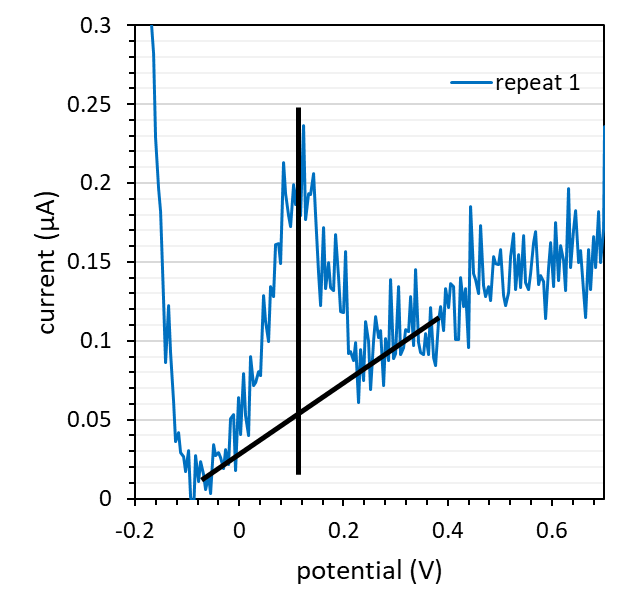

  - Adjust the y-axis minimum and maximum so that you can see the peak clearly.
- Use the diagonal ruler to draw a baseline across the bottom of your peak.
- Imagine a Gaussian bell shaped curve (since we have not yet integrated peak fitting into the template spreadsheet), and use your eye to determine the highest current position achieved by the peak. Try to ignore the contribution of electrical noise by imagining the smoothed-out peak. Write this number down.
  - In this case, we would say that the peak maximum is at ~0.19 μA.
- Determine the peak minimum from where the two rulers intersect. Write this number down.
  - In this case, we would say that the peak minimum is at ~0.055 μA.
- Subtract the peak minimum from the peak maximum and write this number down. This is your peak height.
  - In this case, we would say that the peak height is 0.19-0.055 = 0.135 μA.
- Right click on the graph and left click on *Select data*. Hide repeat 1 and show repeat 2. Calculate the peak height in the same way, and repeat.
- If you are happy that your peak is stable (e.g. <10% variance in the peak height) you can move on and add a small volume of your standard solution. Otherwise, run some repeat scans, paste the data below the previous results, and calculate the peak height again.
- The spreadsheet template has additional spreadsheets and figures for processing the data collected after each standard addition.

## Arsenic determination using the method of standard additions

Now we will quantify the concentration of arsenic in a real sample. We use the method of standard additions, which is a form of internal calibration. We first measure the sample, looking for an arsenic peak. We then add a small volume of a concentrated arsenic stock solution to *spike* the sample, increasing the intensity of the arsenic peak. We add the standard at least two times. We create a graph where the x-axis shows the concentration of the arsenic standard added and the y-axis shows the peak height. We then fit a linear regression (a line of best fit) to the data and extrapolate back to the x-axis intercept to determine how much arsenic was present in the sample before we added the standard solution. The advantages of this method versus external calibration are that as long as we only add small volumes of our standard solution, the concentration of all matrix components (other than arsenic) stay the same. The interference due to matrix effects is thus constant between all our measurements.

- We need to ensure that all our experiments fall within the linear range. For anodic stripping voltammetry (ASV), our working region between the detection limit (LoD) and end of our linear range is small compared with other techniques, e.g. one order of magnitude. Our method optimisation gave a LoD of ~0.7 µg L^-1^ and a linear range up to 10-20 µg L^-1^. The limit of quantification (LoQ) is around three times the LoD. Consequently, we should try to ensure that all our measurements (both the sample and the sample with all standard additions) take place with between 2 and 10 µg L^-1^. We dilute our sample so that it fits inside this concentration window. (You will probably have a different LoD and linear range if using different working electrodes or different experimental conditions).
- It is easier to add more arsenic (whether it is the sample or the standard) than it is to remove the arsenic. So let’s assume that our unknown sample has a lot of arsenic, and we only want to use a small amount of the sample. The highest concentrations we are likely to encounter is 100 µg L^-1^. So how many times do we wish to dilute the sample?
  - If we dilute the sample 30 times, we will have a concentration of 3.3 µg L^-1^, which fits nicely inside our concentration window (bearing in mind that after our standard additions we would aim to double the As concentration to 6-7 µg L^-1^).
- Our solution should be acidified to pH 1 and have a total volume of 40 mL (using the small sample cell). How much sample should we add? How much 1 M HCl and how much deionised water should we add?
  - 40 mL diluted 30 times is approximately 1.3 mL.
  - We need 4 mL of the 1 M HCl to give us 0.1 M HCl for pH 1. The rest of the solution (34.7 mL) should be deionised water (though we can add 35 mL for simplicity, it doesn’t matter much – the only volumes that we need to be precise with are the sample and the standard).
- If we are only using a small volume of sample, we will normally prepare the blank electrolyte first (0.1 M HCl) and measure this to ensure that no As is detected in the electrolyte. Do this.
- Now add your sample, stir magnetically, and perform another measurement.
- Do you see an As peak? We can use data from the linear range experiment to guess how much As we have, and decide on whether or not we need to add more of our sample to achieve a good As peak.
- Using the linear range graph below, how much As is roughly present in the sample cell right now? If we need to add more sample to reach 3 µg L^-1^ total As in the sample cell, then go ahead and do it.
  - If the additional volume of sample required to reach our desired As concentration is too high, then it may be best to get rid of the sample and start again. For instance, if the sample has <5 µg L^-1^, then we wouldn’t add any deionised water, we would use 9 mL of sample and 1 mL of 1 M HCl to acidify the sample.


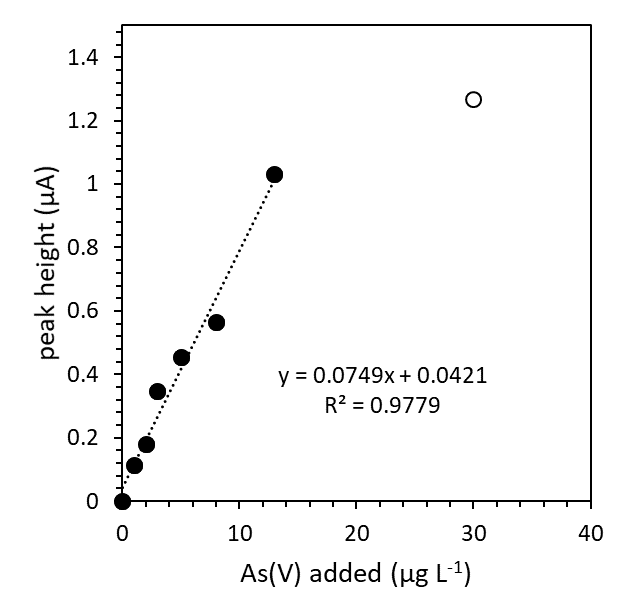


- When you are happy with your As peak, measure it three times. If we peak height varies by >10% or >20%, then measure it more times.
- When you are happy that you have good data for the sample, it is time to make your first standard addition. Using the linear range graph (or your best instincts/prior knowledge of the sample) estimate how much of the 1 mg L^-1^ stock solution you would need to add to increase the peak height by 30-50%. (We aim to increase the peak height by 100% by the end of our experiment, and our experiment should have 2 or 3 standard additions). Bear in mind that it is better to add too little standard rather than too much.
- Make your first standard addition, mix the solution, and measure once.
- If your peak did not increase by as much as you wanted, then add more of the standard and measure again.
- When you are happy with the peak height, measure the sample 3 times. If the results are inconsistent, measure additional times.
- Make your second addition and measure 3 times.
- Make a third addition if needed (i.e. if the earlier data is inconsistent, or if the peak height of the sample has not increased very much with your standard additions).
- Open up the standard addition template in Excel. Duplicate the template spreadsheet and change the name. Add any details of your sample to the spreadsheet (i.e. the name of the sample).
- Enter the volume of the electrolyte added (i.e. deionised water plus HCl, everything that wasn’t the sample or the standard)
- Add the volume of the sample added.


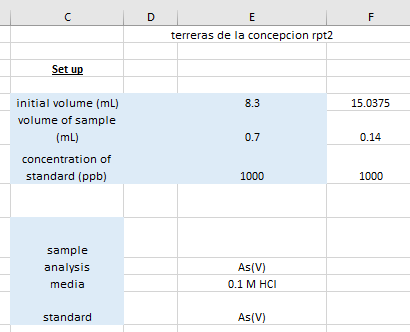


- For each measurement (providing that the data was reasonable), enter the peak height in µA into the data table.
- For each standard addition you made, enter the volume of the standard added (µL).
- The spreadsheet will automatically calculate the concentration of the standard added.
- You may have to adjust the formulae in the columns with headings *average (µA)* and *stdev (µA)* if you made more or less than 3 measurements after any given standard addition.


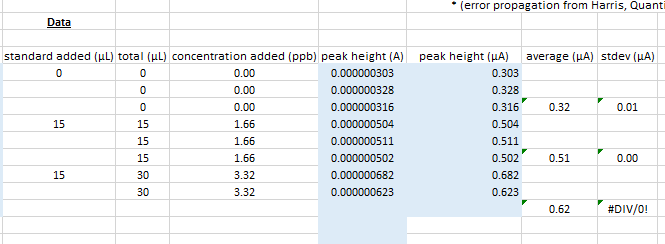


- The spreadsheet uses the LINEST function to fit a linear regression to the individual data points. This linear regression is extrapolated back to the x-axis intercept to determine the initial concentration of As in the sample cell before the standard was added. This is reported as the concentration of the diluted sample, with an uncertainty calculated as recommended by Harris’ Quantitative Chemical Analysis.
- Below this, the original concentration of the undiluted sample and its associated uncertainty is provided.
- The fitting of the linear regression to the data is provided by the graph. The R^2^ value should be >0.95 to provide a good quantitative description. The typical uncertainty in this measurement is ~10%.
- If the linear regression has a poor R^2^ value and the uncertainties are very large, then you can either measure the sample again (checking electrodes are securely connected) or you can try to identify outlier data points. By deleting outliers, you can improve the quality of the linear regression, decreasing your uncertainty and hopefully improving the accuracy of your measurement.


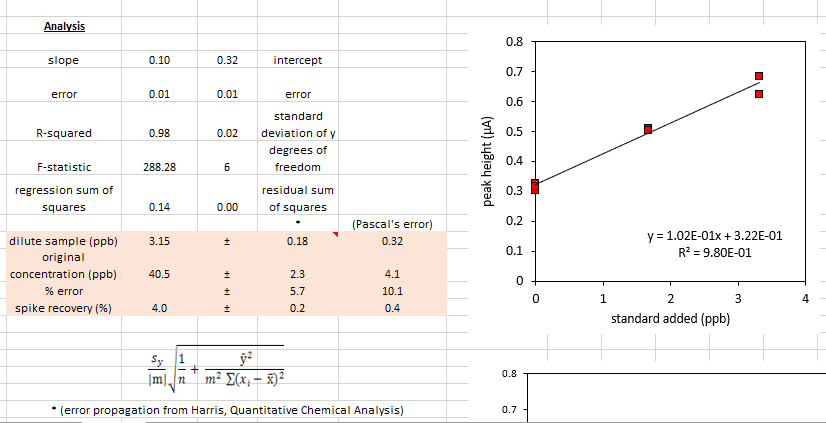


## Recommended procedure for total As determination

- If the sample is likely to be <6 ppb, do not dilute the sample (based on our 0.7 ppb LoD and 10-20 ppb linear range). Otherwise, dilute with deionised water to achieve ~3 ppb. Acidify to pH 1 with 1 M HCl (i.e. add 1 mL to a total volume of 10 mL).
  - If you do not have an initial estimate for the arsenic concentration, assume it is high, and add a small volume of the sample. You can always add more of the sample later on.
- Measure the sample 3 times (20s deposition analytical scan and 1s deposition background scan). If the variation in the As peak height is large, measure a few additional times.
- Make the first standard addition, aiming for a 30-50% increase in the peak height. Be conservative, as you can always add more standard later. When you are happy with that the peak has increased by 30-50%, measure 3 times or potentially more if the data variance is high.
- Make the second standard addition similarly.
- Plot the data. If the data variance is high, or the peak height has not increased by much, make a third standard addition.
- We find that the typical uncertainty of the final arsenic concentration measured using this technical and 6-9 data points (i.e. 2 additions and 2-3 repeat scans each time) is ~10%. If you require a smaller uncertainty for a more precise measurement, try adding more repeat scans so that outlier data points can be removed, or try repeating the entire measurement 1 or 2 times. It is good to perform the repeat measure on a different day, if possible, and perhaps in a different window of the linear range, to minimise any possible systematic errors.

## Determining the linear range and detection limit (LoD)

- The linear range is essential for determining how much arsenic you can add to your sample and still determine the initial concentration using linear regression. To do this, start by measuring your blank electrolyte (0.1 M HCl) once. Add a small volume of arsenic (say 1 μg L^-1^) and take a measurement. Repeat the measurement, multiplying the arsenic concentration by x1.5 each time. For example, you might measure the following cumulative concentrations of arsenic:
  - 0, 1, 2, 3, 4.5, 6, 9, 13, 20, 30, 45, 70 μg L^-1^.
- Stop the experiment when it is obviously that the relationship between the concentration of arsenic added and the peak height is no longer linear.
- The linear range is identified from a graph of As stripping peak height versus the concentration of As added as the region which can be fit with a linear regression (i.e. the region where linear calibration is valid).
- The typical procedure to determine the limit of detection (LoD) is to add the smallest concentration of As that gives a distinct peak (e.g. 2 or 5 μg L^-1^ on the PalmSens2 gold microwire system used in our study) and to measure the sample at least 7 times. The limit of detection is equal to 3.3 times the standard deviation in the peak height, and the limit of quantification (LoQ) is equal to ten times the standard deviation.

# An anecdotal guide to troubleshooting

## Bubbles

### I am seeing bubbles when I shouldn’t

- Most likely the reference potential is wrong. Check that the electrodes are connected in the correct configuration. Check that the reference electrode doesn’t have bubbles. Reconnect as needed. The reference electrode may occasionally need reconditioning – we never had to do this during our study. PalmSens have a procedure for re-plating the silver/silver chloride reference electrode, however it uses chronoamperometry which is a technique that our homemade Rodeostat software cannot yet perform.

### I’m not seeing bubbles when I should

- Again, check the electrode connections and the condition of the reference electrode.
- Try switching off the stirring and vibration during deposition to make it easier to observe bubbles visually.
- Did you add acid to the solution? You will only evolve bubbles under acidic conditions.
- Occasionally we were able to perform successful arsenic analysis despite not seeing bubbles evolved. Additionally, the bubbles formed at the auxiliary electrode are normally more visible than the bubbles at the working electrode.

## Electrode conditioning

### I only see noise

- How big is the noise in absolute terms (i.e. how much current?)
- If the noise is very small, then it is likely that the electrodes aren’t connected and you have an incomplete circuit preventing the current from flowing.
- If the noise is very large, then perhaps one of the electrodes (typically the working electrode) is broken.


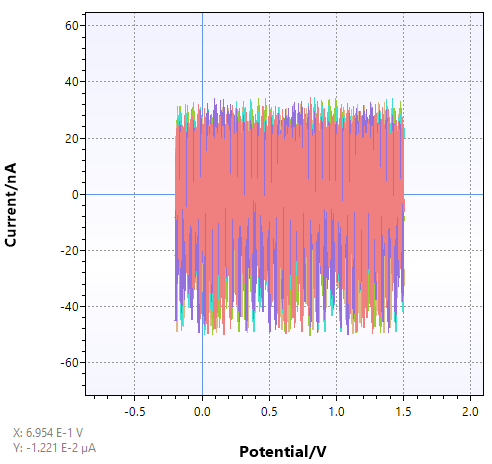


Only noise detected during CV scan when the auxiliary electrode became disconnected from the electrochemical circuit. Note that the current on the y-axis is very small (nano amps).


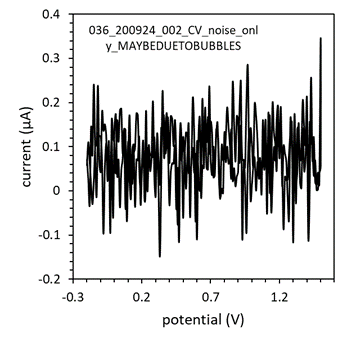


Noise on commercial printed electrodes due to bubbles preventing contact between electrode and solution. No real current measured (<1 uA).

### I don’t see any peaks


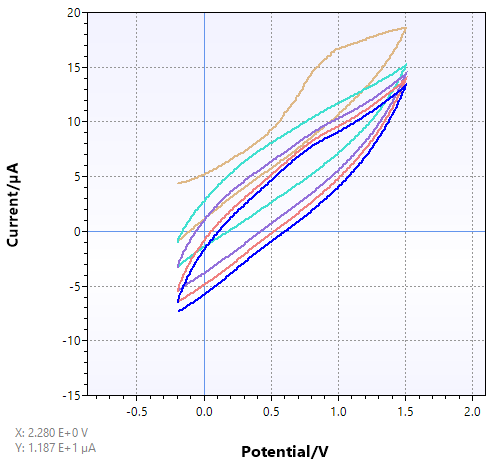


CV scan of working electrode where the gold microwire had either snapped off or dissolved off. This is unusual, we are more used to seeing no signal and only noise when the gold microwire has broken.

### The peaks are in the wrong place

- Maybe you have a pair of distinct oxidation and reduction peaks, but they occur at the wrong potential (e.g. >0.2 V from the expected positions).
- Are the heights of the peaks different to normal? If the peaks are very large and at the wrong potential, then this typically means that another metal is also in contact with the solution, i.e. a component of the electrode with a larger surface area than the gold microwire electrode and thus with greater conductivity. Perhaps copper is in contact with the solution: the gold microwire electrodes are connected to the potentiostat via a copper wire. The older, home-made gold microwire working electrodes we have used previously have a silver adhesive to connect the gold microwire with the copper wire, and if this silver adhesive comes into contact with the solution, you will see the same phenomenon.
  - Try changing the working electrode and see if the problem disappears.
- Are the heights of the peaks as expected? If so, maybe you have a problem with the reference electrode.
  - Check that the reference electrode doesn’t have any bubbles. A small bubble at the top of the electrode is okay, otherwise, gently tap the electrode with your finger to loosen up bubbles.
  - The 3M KCl solution inside the electrode may need replacing.
  - Try a new reference electrode.
  - Occasionally Ag/AgCl reference electrodes need to be re-plated with AgCl. We have never had to do this. Procedures for this can be obtained from PalmSens.

### The peaks are too large (or even off the scale)

- This is normally due to another metal component of the electrodes coming into contact with solution as discussed earlier. Try changing the working electrode.

### The peaks are too small

- Check that the gold microwire electrode is not broken.

### Background current is shifting between repeat scans

- This potentially indicates that the gold microwire has become coated with organic residues from the epoxy resin (used in the fabrication of our particular microwire electrodes). The electrode might still be able to detect arsenic, but the sensitivity of the electrode towards detection of arsenic may be less stable, requiring repeat scans, and the sensitivity may be decreased.


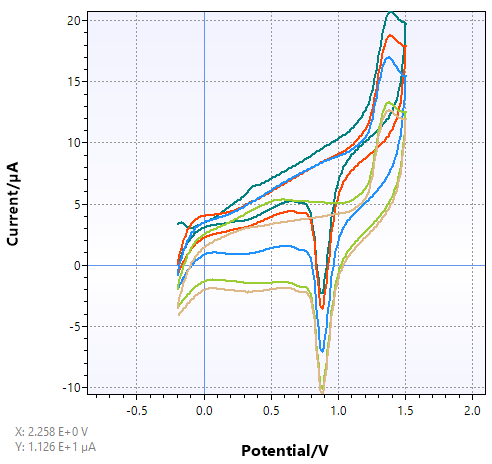


### Correct peaks but very large current

- We are not sure what has caused this, but it was not possible to measure arsenic with this particular electrode. Probably due to the large current leading to large scale evolution of oxidants during the deposition stage, interfering with the detection of arsenic.


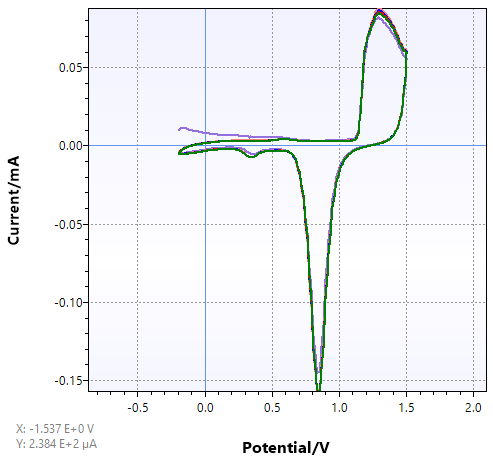


Note that the current of the reduction peak is about -150 μA. This is ten times greater than we normally observe for the 3μ um diameter working electrodes.

## Arsenic detection

### No arsenic peak detected

- What is the shape of the voltammogram? You should have negative current at -0.4 V, approaching zero current at -0.1 V, and roughly flat between -0.1 and +0.7 V.
  - If the voltammogram is flat, with no negative shoulder at low potentials, then perhaps you forgot to acidify the solution.
- If the background shape is correct, but your sample shows no arsenic peak, try adding 10 ppb of your arsenic standard. If you don’t see a peak, then something is wrong. If you see the peak, then perhaps your sample was very low in arsenic.
- If you have a positive peak at -0.2 V, this is related to build up of oxidants or the break-down of the epoxy resin binding the electrode. When this peak is several μA in intensity, the arsenic peak will be supressed. To remove this interference, try increasing the stirring and using vibration. Often the interference disappears when using a new electrolyte.
- When the negative current at -0.4 V is very strong, this also seems to supress the As peak. We are finding that the current at -0.4 V will converge at around -5 μA after the electrode has been scanned many times, and the arsenic peak is stable. If the current at -0.4 V is much more negative than this, then arsenic detection will be more difficult. You may need to improve stirring and vibration, prepare a new solution, or even change the working electrode.

### The arsenic peak is too broad (e.g. it overlaps with other features in the voltammogram)

- Check the pH of your sample solution. You should be measuring total As at pH 1. If the sample is highly buffering (e.g. a lot of carbonate or organic matter) then you may need to add additional HCl to achieve pH 1.
- Consider adding more HCl – ASV peaks are narrower (better resolution) under increasingly acidic conditions.

### The arsenic peak is not ‘bell shaped’

- The arsenic peak may be overlapping with an interference (see following sections)
- If the peak shape changes with repeat scans, then you might have interference from ripples in the solution (if the stirring and vibration is continuing too late – try turning it off sooner!) or it may be electrical noise from a bad connection.

### There is a second peak overlapping with the right-hand side of the As peak

- This interference is due to the condition of the working electrode, and is associated with the negative deposition potentials used under acidic conditions. Sometimes the interference increases, sometimes it decreases. You may find that after extensive use, the interference improves.
- You may need to add more sample so that your arsenic peak dominates this interference.
- You may need to replace the working electrode.

### There is a second peak at around +0.3-0.4 V

- This is normally copper, which can be found in your samples, sometimes from copper dust/trimmings from old bits of electrical cabling, or potentially even in the deionised water supply. The copper peak shouldn’t overlap with your arsenic peak, unless your peaks are too broad (requiring further acidification). Just bear in mind that your baseline has to go underneath/past the copper peak.

### Random variation in arsenic peak height during repeat scans

- The arsenic peak height depends upon the arsenic deposition rate. The arsenic deposition rate must be constant to ensure reproducible peaks. The magnetic stirring is normally constant and reliable. However, the mechanical vibration can be inconsistent. Is the tone and noise of the vibration constant or is it changing? If the vibration is changing, then try removing the vibrator and re-assembling. Try reconnecting the vibrator to the controller, ensuring a good connection. If the vibrator is inconsistent, then try removing or not using the vibrator – it is fine to not use the vibrator as long as you don’t get the build-up of interference at -0.2 V and a negative current at -0.4 V.
- If the electrode connections are loose, then the electrical circuit may be disconnecting during deposition. We found this was the case when using the vibration and a loose auxiliary electrode connection – it looked like the auxiliary electrode was disconnecting from the circuit during vibration, resulting in the circuit breaking, and arsenic not being deposited effectively. This problem was solved by better securing the electrode.
  - Ensure that you don’t nudge the electrodes during your experiment. Try to pipette your sample carefully through the pipette port in the cell lid, without nudging anything.
- Also, ensure that you are turning off the stirring and vibration during the anodic stripping step (e.g. the LSV step). Stirring and vibration cause ripples in the solution, and this motion will cause a current that is measured and super-imposed on your voltammogram. These waves of current will interfere with your arsenic peak.

### Systematic decrease in arsenic peak height during repeat scans

- Check for interference at -0.2 V (positive current) and check for negative current at -0.4 V. If you have a peak at -0.2 V with a peak height of a few uA, or if you have more than -10 uA at -0.4 V, then these interferences are likely supressing arsenic.
- Decreases in the As stripping peak when detecting As(III) only (and not total As) are normally due to oxidation of the As(III). Check that your sacrificial oxidant (i.e. hydrazine) is working. Consider a nitrogen purge to remove oxygen, if available.

### Systematic increase in arsenic peak height during repeat scans

- Could be caused by crud stuck to the working electrode surface, that is cleaned off during repeat scans, increasing sensitivity.

### Arsenic peak height plateaus with increasing standard addition

- Are you still within the linear range? Our microwire electrode with 20 seconds deposition and 1.2 V s^-1^ scan rate using LSV has a linear range up to around 20 ppb.
- If you are within the linear range, and the standard addition curve is non-linear, instead reaching a plateau, then this means that the electrode sensitivity is decreasing.
  - Check for interference at -0.2 V or negative current at -0.4 V: these will sometimes build-up during the experiment, and supress the arsenic peak. Also check for a chlorine smell.
    - Increase stirring rate or use vibration to prevent bubbles and organics from sticking to the electrode surface.
    - If you are unable to prevent this build-up during your repeat scans, then you may have to measure your samples with fewer repeat scans, so that you can conduct a full experiment with fewer total scans.

### All my final results are higher than I expected

- If you are outside the linear range, then you will get a smaller-than-expected increase in the arsenic peak height when you add the standard, resulting in a systematic positive error to your final results.
  - Check you are within the linear range. You can do this by preparing a blank electrolyte and adding sequential additions of your arsenic standard.
- Otherwise, the electrode sensitivity could be decreasing during analysis. Check for the build-up of interference at -0.2 V or negative current at -0.4 V, since these supress arsenic detection. Improve stirring and vibration to supress these interferences.
- If you are too close to the LoD, with a small arsenic peak, then you may be including curvature from the background scan in your analysis. Try adding more sample to develop the As peak further.
- Maybe your standard solution is the wrong concentration. Try making a new standard.

### All my final results are lower than I expected

- Check that you are not too close to the LoD – you may not be identifying all arsenic present, especially if there is curvature in the background scan.
- Check that the electrode sensitivity isn’t increasing during analysis. This can happen due to crud being cleaned from the electrode surface during analysis (for instance with one electrode. At one point, we were seeing interference at +0.35 V decreasing with repeat scans whilst the As peak increases with repeat scans. By scanning the sample multiple times, waiting for the peak height to converge, before adding the standard solution, and ignoring the early data in our calculations, we can ensure that the electrode sensitivity is the same across our experiment. To check that the electrode sensitivity isn’t increasing during analysis, simply perform repeat measurements, checking the As peak height.
- Maybe your standard solution is the wrong concentration. Try making a new standard.

## Steps taken to resolve uncommon problems

- Check that the circuit is set-up correctly (maybe electrodes have become disconnected or perhaps the electrodes were connected in the wrong configuration).
- Check that the electrodes are intact and not broken. You should be able to see the hair-like gold microwire electrode. The reference electrode should not have any air bubbles (a small bubble at the top of the electrode is okay. Flick other bubbles with your finger until they float to the top). The reference electrode should consist of a silver wire with a darker, brown silver chloride coating on the surface. The reference electrode may need conditioning when it is not behaving appropriately. PalmSens have a technique for this, but it uses chronoamperometry and our homemade software does not yet have this functionality.
