# Supplementary material for: Low-cost electrochemical detection of arsenic in the groundwater of Guanajuato state, central Mexico using an open-source potentiostat
Source: PLoS One. 2022 Jan 19;17(1):e0262124. doi: 10.1371/journal.pone.0262124 (PMC8769315; doi:10.1371/journal.pone.0262124)
Supplement: Supplementary file 21 [file pone.0262124.s002.zip › Software version 2 (updated and including relay control)/CaminosTestV19Jun21- Documentation.odt]

Caminos de Agua

Arsenic Test Potentiostat 

Software Installation and Operation

Larry Dworsky

Caminos de Agua, San Miguel de Allende

Version 19-June-2021

@ 2021  Caminos de Agua.  All Rights Reserved

## 1  Introduction

The [Rodeostat](https://iorodeo.com/collections/cheapstat-open-source-potentiostat/products/potentiostat-shield) potentiostat is supplied with a PC driver interface written in Python. This interface enables a user to issue commands to the Rodeostat for configuring the Rodeostat, setting probe voltage and measuring probe current. A user program has been written, in Python, to facilitate this task. This program reads data from a script file which describes a potentiostat “run”, performs the run, graphically shows the results of the run and, optionally, saves the results to a file.

This document describes the installation procedure for the software, structure of the data file and available commands. Several sample files and results are provided.

The Rodeostat PC driver software will only work on a 64 bit Windows PC.

It is assumed that the program installer/user is familiar with the Windows operating system - including specifying file locations, opening a command window, creating folders, etc. Use of a text editor is necessary for creating and modifying (ASCII text) data files. The Notepad editor included with a Windows installation will do the job. A more convenient text editor is the downloadable [Notepad++](https://notepad-plus-plus.org/) in that it allows several files to be open at once[^1]. Any word processor will also do the job, but remember that word processors do not save files as ASCII text files by default.

## 2 Installation

The name of the program is CaminosTestV19June21.py (hereafter referred to in this document as Test.py). This is a Python program. In order to run this program, there must be a Python 3 installation on the computer. I recommend the [Anaconda](https://www.anaconda.com/products/individual) Python Individual Edition. Any other Python should work, but the installation and setup instructions to follow are for the Anaconda distribution. If you are using a different Python distribution, learn how to install necessary packages and install them as requested. You'll have to figure out the required procedures. I have not tried running Test.py on a computer with multiple Python distributions installed - good luck.

### 2.1 Install Python and Required Packages

Download Anaconda Python, 64 bit version. Select all the options offered. The user interface is called Spyder. There is a Spyder tutorial provided. Even if you don't plan to examine or modify the source program, it's a good idea to look at the Spyder tutorial.

Create a folder with your choice of name and location on the disk. This is where you'll put the program and your data files. Create a subfolder in this folder and name it AutosavedFiles; the use of this folder will be explained below. These file names and locations are not necessary choices, but these are the default locations written into the program, so they are the most convenient choices.

Copy Test.py, snapshot.py and any files ending in .ast to the chosen folder. It would be prudent at this time to make a copy of Test.py in case it is accidentally deleted or corrupted. Copy the folder USB8RelayManager to the chosen folder. A copy of this documentation may also be put in this folder, for convenience.

There are two Python packages which are needed that are not included with the Anaconda installation. The standard package installation program, “pip” is included with the Anaconda installation. Open a command window and move to the folder c:\ArsenicSept2020. Type the following lines, finishing each line with the Enter key. Wait for each installation to finish before going on.

- pip install iorodeo-potentiostat

- pip install easygui

### 2.2 Set up the computer for relay board control

Test.py can control four relays for stirrer control and/or other operations. The relay board used is a Sainsmart USB 4 Channel Relay #B543240528. A relay board is not necessary for using Test.py to control the Rodeostat Potentiostat. Rather than having two separate versions of the software, however, it is easiest to set all installations up consistently as if the relay board were present.

1.  Plug the Rodeostat USB connector and the Relay board USB connector to the computer.  Note that Relay board has 2 cables with USB connectors attached. These are labeled USB and Power. Only plug the USB connector to the computer at this time. Do not connect the Power cable to anything.  The computer might present a window “Setting up new USB ….”  If so, do not continue until this is finished.

2.  On the computer, open up the Device Manager (Control Panel → device manager). Expand “Ports.”  You should see two entries:  USB Serial Device and USB Serial Port.  These are the connected Rodeostat and Relay board, respectively.  Double Click on Serial Device. Go to Port Settings → Advanced.  From the dropdown menu, set the port to COM200.  Hit OK until you are back at the device manager.  Double Click on Serial Port and repeat the same procedure, setting this port to COM220.  Note that these ports will reset to their default values when the computer reboots.  Close the device manager.

4.  Plug the AC power cord from the relay board into AC power and the Power USB connector from the relay board into a USB power supply.  There should now be two LEDs on the relay board turned on – one indicating USB connection, one indication AC 5 volts power (from the USB power supply.

5. Open the USB8RelayManager folder. Double click on the program USB 8 Relay Manager v.1.4.exe.  Click on Open Relay Board, then Manual Mode.  You should now be able to click on Relays 1 – 4 On and Off.  You should here the relays clicking and see their LED lights turn on.

### 2.3 Test the Installation

There are two ways to run the program. It doesn't matter which way is chosen. The first way, however, will produce more useful error information is something goes wrong.

Note that it is NOT necessary to connect the Rodeostat to the computer at this time. Test.py has an internal emulation mode that it defaults to when it cannot find a Rodeostat connection. Test.py will print a notice that it could not find a Rodeostat and is defaulting to the emulation mode. This mode was written for software debugging, it can usually be ignored by a user.

1. From inside Spyder, go to file, open. Navigate to the folder where you put Test.py and select it. The full source program will open. You can inspect and/or modify this program if you wish. Be careful to avoid unintentional modifications. Click the solid green right-pointing triangle which is under the word “source' on top of the screen; this runs the program.

The program will open a dialog box so that you can specify a data file[^2]. Navigate to the same folder as in step (1) and select RodeostatTestFile1.ast

Select the “plots” option in the upper right Spyder window. The program will immediately produce a “snapshot” of the voltage profile specified in the data file. This snapshot is for the user’s convenience – the user can visually verify that the data file has been constructed correctly (or not). The program will then present a choice to continue or to terminate the run.

Assuming that the choice to continue has been selected, the program will echo the input data file to the screen and then proceed to emulate the Rodeostat. If the AutoSave option has not been chosen in the data file, a dialog box will open asking to save the output file. If this option is chosen, the program will then prompt for a file name and a folder name. See below for AutoSave option specifics.

At the end of the run, the Sypder Plots window will show the voltage profile (as in the preview) along with the resulting probe current response.

2. Using Windows Explorer, navigate to the folder containing Test.py, and double click on Test.py[^3]. A program output window will open, appropriate dialog boxes will appear (and then disappear) as needed. When the data file is selected, the voltage preview will be shown in a new window. When running this way, the prompt to continue will not appear until the plot window is closed.

Other than in appearance, it doesn't matter which way you open the program. Spyder has the advantage that, if a problem does occur, a (hopefully useful) error message will appear. If the program is started from Windows Explorer and gets in trouble, it will simply close and all the opened windows will disappear.

The Rodeostat should now be connected to the computer using at a USB port. Windows typically plays a small jingle when a USB device is connected or disconnected, you should hear this jingle.

6. Test the relay board control.  The included file relay_test.ast may be used for testing if desired.  Watch the program output carefully – if a “Com port not found” message comes up:  Kill the program (or let it finish in simulation mode if you wish), disconnect the USB cable to the Rodeostat and reconnect it.  This will happen occasionally the first time the program is run after setup, if the program is run first by double clicking on the Test.py entry in the file directory and then running the next time from inside Python, or vice-versa.

## 3 Data File Preparation

Data files for Test.py have the file type ....ast (AS Test). They are standard Comma Separated Variable (CSV) ASCII text files. They may be created, read and edited in any ASCII text editor. Be careful if using a word processor rather than an ASCII text editor. Most word processors can save files as ASCII text files, but this is not their default file format.

Note: In some of the commands listed below, there are optional parameters. These are shown in [ ] brackets below, (don't use these brackets in the actual file). If the option is not desired, simply ignore it as shown in the examples.

### 3.1 Autosave:  Autosave, Folder, [Echo]

Example: Autosave, c:\ArsenicSept2020$\AutoSavedFiles

Example: Autosave, c:\ArsenicSept2020$\AutoSavedFiles, Echo

If this line not included in the data file, the user is prompted as to whether or not to save the output files. If the user chooses to save them, there is a prompt for the file names and locations. If this (Autosave) line is chosen, then there is no file selection prompt - all files are automatically saved to the specified location. Autosaved file names specify "main" or "ramp" and have a date and time to included in the file name, the file extension ...dat is appended to each file. These are CSV files.

Notes:

- There is a main file which saves everything.

Example: Main 2020-11-23-11-45-32.dat

- If "ramp" commands are part of the data file, there are also a separate "ramp" files generated.

Example: Ramp 2020-11-23-11-45-32.dat

If the optional Echo is included, the input data file is echoed to the top of the Autosaved main file.

In general, Autosave with the Echo option are recommended choices. If you don't save a file you might regret the loss; if you save all files then at worst you'll want to delete some unwanted files. If you include the Echo option, then there will never be any confusion as to what parameters were used to generate this data.

### 3.2  NoPlots

If Autosave is NOT chosen, the command NoPlots may be chosen (if Autsave is chosen, NoPlots will be ignored).  When Autosave is not chosen, the program presents voltage and current graphs after each Ramp command and pause the program until these graphs are closed.

If NoPlots is chosen, these plots and the associated pauses will not occur.  Note that the final plots and option to save the data file are not affected by NoPlots.

### 3.3  Mandatory Lines

Each data file has two mandatory lines either at the top of the file or just after an Autosave command line. These lines set parameters for the run, as will be explained below. Any number of lines may be added below these two mandatory command. Lines.

dt, num1

Example: dt, 0.01

num1 is the time increment, in seconds, of how often the Rodeostat sends / acquires information. All timing information in subsequent commands refer to multiples of dt. Too large a value of dt will cause a loss of resolution. Too small a number can cause the creation of very large output data files and will ultimately exceed the Rodeostat speed capabilities. The Rodeostat can handle .005 seconds; values down to .001 seconds are accepted but timing errors may occur.

PotParams, num1, num2

Example: PotParams, 2, 3

The Rodeostat sets itself up in different ranges, tradeoffs between dynamic range and resolution. Num1 is the maximum voltage magnitude allowed, num2 is the current measurement range.

- num1 = 1 or 2 or 3 or 4 -> 1, 2, 5, 10 volts

- num2 = 1 or 2 or 3 or 4 -> 1, 10, 100, 1000 microamps

The following commands are not mandatory; you can choose which ones you want, in which order, and as few or as many as you want. They will be executed in order. The time counter in the software runs continuously –  it does not restart at each command.

### 3.4  Comment Line:    # arbitrary text

Example: # The following command is a very long constant voltage

A comment line is echoed to the output but otherwise ignored by the program, as is done by many computer language compilers. Comments are for the convenience of the user and serve no other purpose. Note that when experimenting with different data files, it is often convenient to turn an actual command line into a comment by putting a # in the first column of the command line; then it is not necessary to delete and then retype the line if/when you want to put it back as an actual command.

Sample Data File

An example of a correct complete data file, which does nothing, is:

# Speed test file

# Oct 22, 2020

AutoSave, c:\ArsenicSept2020\AutoSavedFiles, Echo

dt, 0.001

PotParams, 2, 3

### 3.5   Constant Voltage:  ConstVolts, num1, num2

Example: ConstVolts, 1., 100

A constant voltage of num1 volts for a duration of dt*num2 seconds. The current is measured at the beginning and the end of the time period. Since ConstVolts durations of several minutes are common, a progress bar is displayed.

Sample Data File

dt, 0.25

PotParams, 2, 3

ConstVolts, 1., 6

ConstVolts, -2., 8

### 3.6  Ramp:  Ramp, num1, num2, num3 [, num4]

Examples:  Ramp, 1., -.5, 20.

		Ramp, Ramp, -.5, 0., -1000.

		Ramp, Ramp, -.5, 0., -1000., 10

A linear ramp from num1 volts to num2 volts. The linear ramp is approximated, automatically, by many small stepped constant voltages.

If num3 is positive, the duration of the ramp is dt*num3.  If num3 is negative, then |num3| is the duration, in msec. If num3 is negative and num4 = 0, the current is sampled every 1/dt seconds. If num4 is > 0, then the current is sampled every num4 msec.

Sample Data File

# Nov 30, 2020

# Test files for documentation

AutoSave, c:\ArsenicSept2020\AutoSavedFiles, Echo

dt,  0.10

PotParams, 1, 3

ConstVolts, 1., 10

Ramp, 1., -.5, 20.

ConstVolts, -0.5, 5

Ramp, -.5, 0., -1000., 10

### 3.7  SquareWave, num1, num2, num3, num4

Example:  SquareWave, .75, -.5, 3., 3

A Square wave. High voltage = num1, low voltage = num2. Num3*dt = Full cycle period in seconds;  num4 = number of full cycles.

Sample Data File

# Nov 30, 2020

# Test files for documentation

AutoSave, c:\ArsenicSept2020\AutoSavedFiles, Echo

dt,  0.10

PotParams, 1.0, 3

ConstVolts, 1., 5

SquareWave, .75, -.75, 2., 3

Ramp, -.5, 0., -1000., 10

### 3.8  SawTooth, num1, num2, num3, num4

Example: SawTooth, .25, .5, 3, 2

A sawtooth with an average value of num1 volts, + and – peaks num2 volts about the average.  Num3*dt = full cycle sawtooth period in seconds.  Num4 = number of total repeats.

Sample Data File

# Sawtooth test data file

dt,  0.25

PotParams, 2, 3

ConstVolts, -.5, 8

SawTooth, 0.5, 1.,  8, 3

ConstVolts, -.5, 4

### 3.9  Alert, num1, [num2, [num3]]

Example:  Alert, 1, 500, 3000

The Alert command has no affect on Rodeostat operation.  It provides program status information to the user.  When an Alert message, e.g. “Alert, 2” is reached in the run, the message

***********

Alert nr: 2

***********

will appear on the screen. Note that the Alert numbers to not have to be consecutive. This is an arbitrary number for the user’s information.

If num2 is provided, e.g. “Alert, 2, 500”, then a 500 Hz note will be played for one second. If num2 and num3 are provided, e.g. “Alert, 2, 400, 3000” then a 400 Hz note will be played for 3,000 msec (3 seconds). These notes are played as a background program operation so that program timings are not affected by them. Also, if a new note is requested before an old note has completed, then the duration of the old note is truncated and the new note supersedes it.

### 3.10  Relay, num1, num2

	Example: Relay, 1, 1         Turn Relay 1 on
	Example: Relay, 3, 0	Turn Relay 3 off

Relay board control for four relays. The original intended use is to control stirrer motors. The fourrelays may be turned on or off independently. At the end of the run, all relays are turned off.

### 3.11  Swas, num1, num2, num3, num4

Square Wave Anodic Stripping.  In practice, the original version was identical to Ramp because the square wave steps were taken as quickly as possible which is identical to how the Ramp commands simulates a linear ramp.  The Swas command has been disabled while the desired result is reconsidered. Note that the Swas command is presented in the literature as a way to improve the Ramp response by canceling some capacitive charging current from the response.

[^1]: All programs described in this document are open source, i.e. are free downloads
[^2]: This dialog box might be hidden behind the Spyder window
[^3]: If the file association for this file has been changed it must be reset, or, instead of double clicking-click and choose Open With → Python.
